# Stepwise polymerization enables tailored perovskite ink for scalable flexible perovskite solar cells

**DOI:** 10.1038/s41467-026-77703-w

**Published:** 2026-09-15

**Authors:** Weifu Zhang, Zhaojin Wang, Hengyu Zhou, Wei Song, Chenfan Xing, Jiahan Xie, Jintao Zhu, Jiaming Huang, Xiaowei Xu, Mengjin Yang, Yang Bai, Ziyi Ge

**Affiliations:** 1 Zhejiang Provincial Engineering Research Center of Energy Optoelectronic Materials and Devices, Ningbo Institute of Materials Technology & Engineering, Chinese Academy of Sciences, Ningbo, China; 2 CAS Key Laboratory of Green Printing, Institute of Chemistry, Chinese Academy of Sciences, Beijing, China; 3 Center of Materials Science and Optoelectronics Engineering, University of Chinese Academy of Sciences, Beijing, China; 4 Faculty of Materials Science and Energy Engineering, Shenzhen University of Advanced Technology, Shenzhen, China; 5 Department of Chemical and Environment Engineering, University of Nottingham Ningbo China, Ningbo, China; 6 Department of Electrical and Electronic Engineering, The Hong Kong Polytechnic University, Kowloon, China

**Keywords:** Solar cells, Energy

## Abstract

Commercializing flexible perovskite solar cells (f-PSCs) is hindered by poor interfacial contact and suboptimal crystallization, particularly in large-area perovskite solar modules (PSMs). Here, we propose an in situ stepwise polymerization strategy utilizing an epoxy-terminated monomer (BFDGE) and a curing agent (isophorone diamine, IPDA). A room-temperature pre-reaction enables precise tailoring of perovskite ink, ensuring reliable fabrication of perovskite films over large-area substrates. Subsequently, thermal annealing synchronizes the polymerization with perovskite crystallization, dynamically modulating the crystallization process. Consequently, rigid and flexible devices yield outstanding efficiencies of 27.16% (certified 26.87%) and 25.13%, respectively. The resultant cross-linked scaffold ensures superior durability, with devices retaining 93.3% of their initial efficiency following 1000 h of maximum power point (MPP) tracking and 96% after 630 h ultraviolet irradiation. Furthermore, minimodules (10.24 cm^2^) achieve high efficiencies of 23.25% (rigid) and 20.59% (flexible), with 95.1% retention after 200 thermal cycles (−40 to 85 °C). Notably, a submodule achieves an impressive efficiency of 22.60% (655.2 cm^2^, certified 21.07%), establishing a new benchmark for large-area inverted PSMs.

## Abstract (web summary)

Zhang et al. developed a stepwise polymerization strategy to tailor perovskite ink. This resolves the poor wetting bottlenecks and optimizes crystallization across large-area substrates (655 cm^2^ reaching 21.07% efficiency), providing a scalable pathway for perovskite photovoltaics.

## Introduction

Perovskite solar cells (PSCs) have emerged as leading candidates in the photovoltaics field due to their exceptional power conversion efficiencies (PCEs), compositional tunability, low-cost, large-area solution printing, and potential for flexible applications. To date, rigid laboratory-scale PSCs have achieved efficiencies exceeding 27%, whereas flexible PSCs (f-PSCs) on plastic substrates still lag behind^1–4^. This performance gap is principally attributed to the rough surface and limited thermal endurance of plastic substrates, which lead to excessive nucleation and yield low-quality crystalline perovskite films with small grains. In addition, the drastic increase in grain boundaries makes the perovskite film brittle and highly susceptible to mechanical failure under stress^5,6^.

These microstructure-induced deficiencies become markedly exacerbated when scaling the technology to larger-area modules. This challenge is acutely apparent when extending high efficiency f-PSCs from laboratory cells to minimodules (10-200 cm^2^) and submodules (200–800 cm^2^)^7–9^. One of the most critical bottlenecks is the poor wettability of perovskite precursor inks on hydrophobic self-assembling molecules (SAMs)-based hole transport layers (HTLs)^10,11^. This interfacial mismatch leads to incomplete film coverage, inhomogeneous distribution, pinhole formation, and compositional segregation during crystallization^12,13^. Such morphological defects not only depress the PCEs of modules relative to small-area devices but also undermine the uniformity and reproducibility essential for commercial-scale perovskite solar modules (PSMs)^14,15^. Beyond the efficiency losses, the operational stability of large-scale PSCs is severely compromised under real-world conditions. The ionic nature of perovskite crystals renders them vulnerable to degradation upon exposure to temperature fluctuations, humidity, oxygen, and continuous illumination^16,17^. And the plastic substrates exacerbate these stability issues due to their higher water and oxygen transmission^18^. Although remarkable progress has been made in boosting the efficiency and stability of small-area f-PSCs by various cross-linkable monomers, their scalability is fundamentally hampered by the difficulty in controlling film homogeneity over larger areas^19–21^. This heterogeneity, manifesting as non-uniform larger-area perovskite films, exhibits localized distortions in electric fields and accelerated ion migration. Consequently, large-area PSM frequently display pronounced performance decay and insufficient long-term reliability^22^. These challenges underscore the urgent need for integrated strategies that simultaneously optimize scalable perovskite crystallization and environmental resilience to realize commercially viable perovskite photovoltaics.

Herein, we introduce a pioneering concept of stepwise polymerization for the precise control of perovskite ink, enabling reliable fabrication of PSMs. We employ an epoxy-terminated cross-linkable monomer, bisphenol F diglycidyl ether (BFDGE), in combination with isophorone diamine (IPDA) as the curing agent. The ring-opening reaction between IPDA and the epoxy groups proceeds in a controlled, stepwise manner under mild conditions. During ink preparation, a room-temperature pre-reaction generates hydroxyl-functionalized oligomers that overcome the wettability issue of perovskite ink on HTLs to ensure uniform large-area coating. Subsequently, thermal annealing synchronizes the further polymerization with perovskite crystallization, dynamically modulating the crystal growth. Ultimately, the resultant cross-linked scaffold serves as a bulk encapsulation medium (BEM), effectively shielding the perovskite from oxygen, moisture and releasing the mechanical stress under deformation. Consequently, the target rigid and flexible PSCs deliver exceptional PCEs of 27.16% (certified 26.87%) and 25.13%, respectively. The optimized devices exhibit robust long-term stability, retaining 93.3% of their initial efficiency after 1000 h of continuous power output at maximum power point (MPP) and 96.0% under 630 h of ultraviolet irradiation. Notably, rigid and flexible minimodules demonstrate PCEs of 23.25% and 20.59% (aperture area: 10.24 cm^2^), while maintaining 95.1% of their initial performance after 200 thermal cycles (−40 °C to 85 °C, ISOS-T-3). More encouragingly, large-area submodule (active area: 655.2 cm^2^) exhibits an impressive PCE of 22.60% (certified 21.07%), representing one of the highest efficiencies for such large-scale inverted PSMs. Therefore, this study provides a universally scalable pathway for manufacturing high-performance, robust perovskite photovoltaics.

## Results

### Material design and the underlying mechanism of stepwise polymerization

**Fig. 1:**
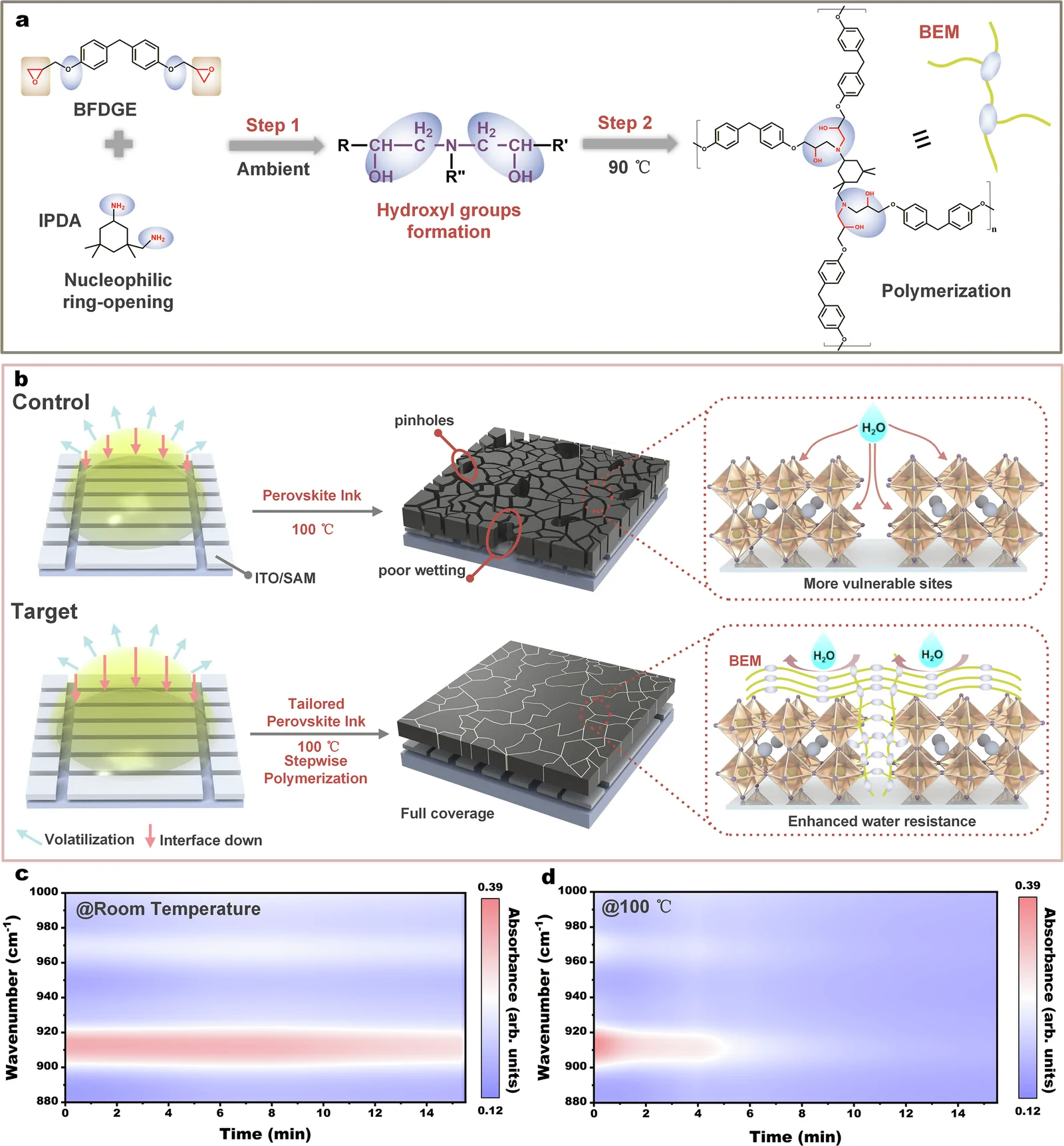
Material design and the underlying mechanism of stepwise polymerization. **a** Ring-opening polymerization reaction between BFDGE and IPDA. **b** Schematic illustration of the proposed stepwise polymerization strategy that enables scalable fabrication and stability enhancement. **c**, **d** In situ FTIR spectroscopy of BFDGE and IPDA mixture at room temperature (**c**) and 100 °C (**d**).

The chemical structures of BFDGE and IPDA are depicted in Fig. 1a, highlighting the functional end groups, epoxide, and amine, respectively. In this study, the epoxide group was selected as the cross-linking site owing to its high reactivity and polymerization temperature, which coincides closely with the thermal-assisted perovskite growth^20^. Notably, the cyclic hexyl-bodied IPDA was innovatively selected over conventional aliphatic hardeners to modulate reactivity. The reaction between BFDGE and IPDA is a stepwise polymerization process, initiated at ambient temperature in the absence of catalyst, followed by polymerization upon heating to approximately 90 °C^23^. As shown in Fig. S1, fresh BFDGE exists as a transparent, viscous liquid at room temperature, and this state can be maintained even after adding 5 wt% of IPDA. Upon gradual heating to 90 °C, the liquid BFDGE, progressively solidifies, achieving complete solidification near 100 °C. Additionally, this polymerization process involves the nucleophilic ring-opening of epoxide groups in BFDGE by the amines in IPDA, contributing to the formation of covalent linkage. The reaction ends with a proton transfer from the amine to oxygen atom, generating hydroxyl groups^24^. Figures S2a and S2b depict the reaction pathway, wherein epoxide groups react with amines to yield secondary or tertiary amines accompanied by hydroxide formation^25^. The resulting predominant polymer structure is also displayed in Fig. 1a, with corroborative evidence obtained from the solution-phase 1H NMR spectrum shown in Fig. S2c. Notably, the primary resonance of IPDA at 1.15 ppm disappears in the spectrum of the resulting polymer (BEM), revealing the participation of IPDA amine groups in the epoxide ring-opening reaction^26^. Moreover, new resonances at 2.22 ppm in the BEM spectrum signifies the occurrence of tertiary amine bonds^27^, suggesting their predominance in the final polymer network.

Building on the preceding discussion, we proposed a plausible underlying mechanism of stepwise polymerization to tailor perovskite ink for the scalable fabrication of f-PSCs, as illustrated in Fig. 1b. The pristine perovskite ink exhibits poor wettability on hydrophobic glass/SAM substrates, which consequently result in suboptimal perovskite film quality. Especially, this issue is even more pronounced during the scale-up process, where perovskite films frequently suffer from the inevitable formation of pinholes^11,28^. Additionally, the uncontrollable crystallization also induces perovskite polycrystalline films, characterized by massive grain boundaries (GBs), which inherently render perovskite films mechanically fragile and generate more sites vulnerable to moisture and oxygen intrusion^16,29^. These difficulties challenge the development of perovskite photovoltaic technologies. To overcome these bottlenecks, a stepwise polymerization strategy was proposed to tailor perovskite ink, aiming to improve both the scalability and stability of f-PSCs. Specifically, BFDGE, a cross-linkable monomer was incorporated into the perovskite precursor solution, with IPDA serving as the initiator. Typically, the reaction between BFDGE and IPDA commences at ambient temperature, although the polymerization remains incomplete at this stage. This stepwise pre-reaction generates intermediate hydroxyl-functionalized oligomers that enhance the wettability of the perovskite ink on SAMs substrate, facilitating the uniform fabrication of large-area perovskite films. This hypothesis is substantiated by the contact angle measurements of the perovskite precursor on the glass/ITO/SAM substrates (Fig. S3). While the addition of pure BFDGE or IPDA only marginally decreased the contact angle (26.0° and 23.0°, respectively), the tailored precursor exhibited a significantly smaller contact angle (16.2°) compared to the control (29.5°). To further quantify this improvement, we calculated the surface tension of the precursors; the tailored ink exhibited a reduced surface tension of 28.5 mN/m compared to 35.0 mN/m for the control (Table S1), which effectively lowers the energy barrier for the ink to spread on the relatively hydrophobic SAMs. Notably, this wettability enhancement demonstrates excellent universality across various SAMs (Fig. S4), and macro-photographs of the perovskite films deposited on the 5 × 5 cm^2^ substrates further confirm the resolution of the coverage issue (Fig. S5). Subsequently, during the thermal annealing process, robust polymerization and perovskite growth are simultaneously triggered. Notably, benefiting from the incorporated agents containing a variety of functional groups, such as carbonyl (-C=O), hydroxyl (-OH), and amine (-NH_2_) moieties, multiple potential interactions between additives and perovskite are possible. The -C=O, and -NH_2_ groups can coordinate with Pb^2+^ within perovskite, whereas the generated -OH groups can bind with FA^+^/MA^+^ through hydrogen bonds as well as passivated iodine-related defects. These interactions playing a vital role in chemical passivation and crystal growth regulation^30,31^. Importantly, the resulting polymer with hydrophobic nature can serve as a bulk encapsulation medium (BEM), which will enhance the moisture resistance and overall stability of the devices.

To explicitly elucidate these dynamic stepwise reaction kinetics, Fourier-transform infrared (FTIR) spectroscopy was performed on the pure BFDGE, IPDA, and their mixture following heating at different temperatures. Static FTIR spectra initially reveal that upon mixing BFDGE and IPDA at room temperature, a peak emerges at ~3400 cm^−1^, confirming the pre-reaction that generates the hydroxyl-contained oligomers (Fig. S6)^25^. To further capture the temporal dynamics, in situ FTIR mapping was conducted to track the characteristic epoxide stretching vibration (~914 cm^−1^). As shown in Fig. 1c, at room temperature, the epoxide signal gradually weakens but persistently remains over 15 min. This demonstrates that the polymerization is intentionally maintained at an intermediate oligomer state without full cross-linking. In stark contrast, during in situ heating at 100 °C, the epoxide signal rapidly diminishes and completely vanishes within ~10 min. This distinct temporal evolution verifies that the ring-opening cross-linking is specially triggered under perovskite annealing conditions. Furthermore, X-ray photoelectron spectroscopy (XPS) was conducted on perovskite films with and without BEM. As illustrated in Fig. S7a, the XPS spectral profiles revealed that the principal peaks at 143.64 eV (Pb 4*f*_5/2_) and 138.77 eV (Pb 4*f*_7/2_) shifted toward lower binding energy regions upon stepwise polymerization treatment (hereafter denoted as Target), specifically to [143.45 eV (Pb 4*f*_5/2_) and 138.59 eV (Pb 4*f*_7/2_)], accompanied by decrease in metallic Pb peak, which indicates that electron donation from the functional groups in BEM to the undercoordinated Pb^2+^ site^32^. Similarly, the peak position of N 1*s* peak (400.90 eV) of the control film was shifted to lower binding energy of 400.76 eV (Fig. S7b), implying that the cross-linking scaffold BEM can form hydrogen bonds with FA^+^/MA^+^ cations^33^. Likewise, the I 3*d* signals at 631.10 eV (I 3*d*_3/2_) and 619.60 eV (I 3*d*_5/2_) also exhibited analogous shifts to 630.94 eV and 619.45 eV, respectively (Fig. S7c), which can be attributed to N-H···I and O–H···I hydrogen bonding interactions between the BEM and the perovskite surface^34^. Collectively, these results provide compelling evidence for the effective chemical passivation imparted by BEM toward perovskite.

### The effect of stepwise polymerization strategy on perovskite growth

**Fig. 2:**
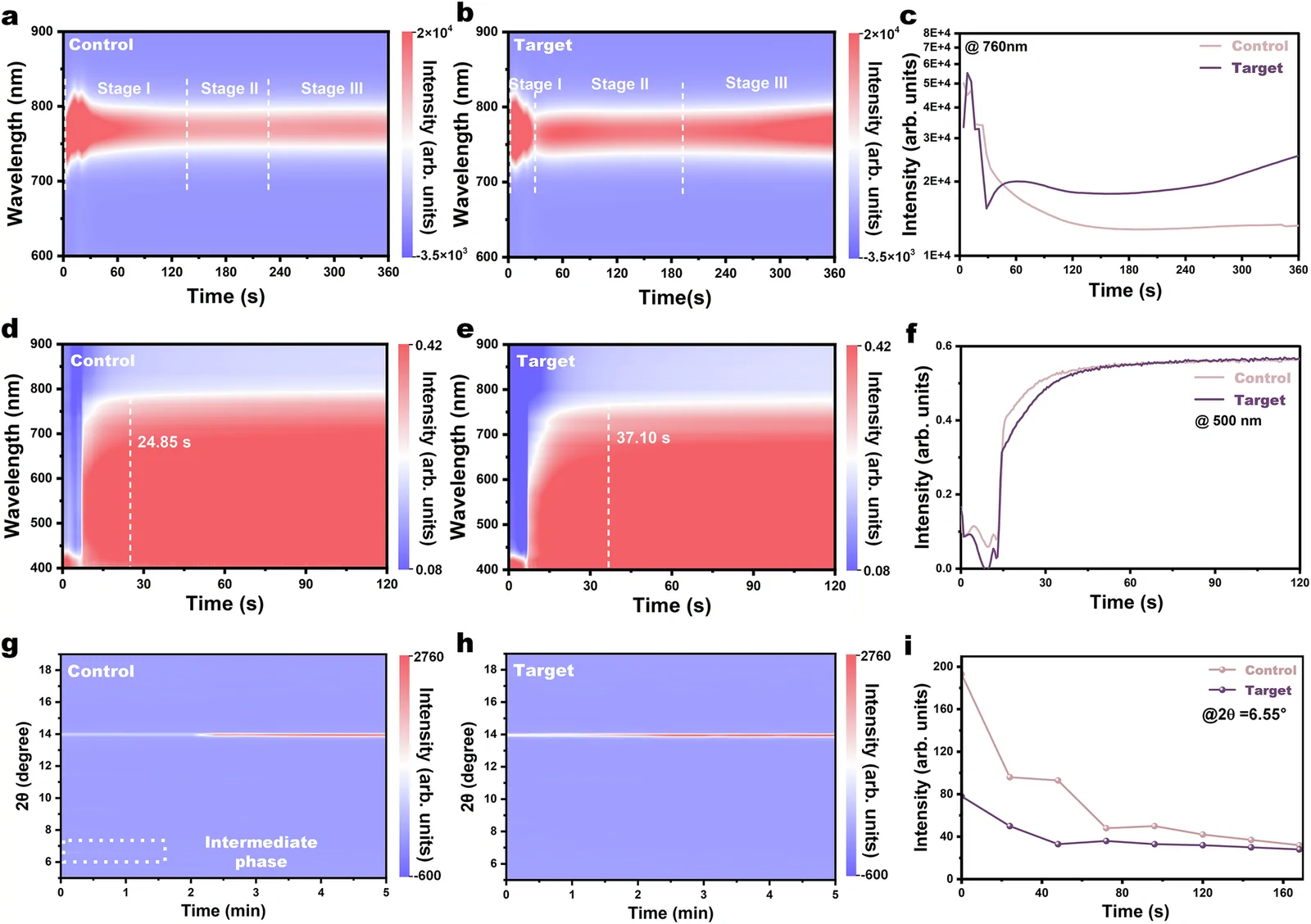
The effect of stepwise polymerization strategy on perovskite growth. **a**, **b** In situ PL spectra of **a** control and **b** target perovskite films during the thermal annealing. **c** The extracted PL intensity versus annealing time curves. **d**, **e** In situ UV-vis spectra of **d** control and **e** target perovskite films during the thermal annealing. **f** The extracted absorption intensity versus annealing time curves. **g**, **h** In situ XRD spectra of **g** control and **h** target perovskite films during the thermal annealing. **i** The extracted XRD peak intensity at 2*θ* = 6.55° of the corresponding films during the annealing procedure.

A critical factor in determining the performance of f-PSCs and f-PSMs involves the crystal quality of perovskite films. To gain deeper insight into the crystallization dynamics modulated by the stepwise polymerization strategy, we performed in situ photoluminescence (PL) measurements during thermal annealing. As illustrated in Fig. 2a, b, the temporal evolution of the PL emission peak position reveals a pronounced three-stage mechanism governing the crystallization process during thermal annealing. In the initial stage, a marked rise in PL intensity is observed, which can be attributed to the increased exposure of crystal nuclei following solvent evaporation. This is followed by a distinct PL quenching phase, interpreted as a consequence of solvent volatilization that promotes the transition from a wet precursor film to a solid-state framework. Subsequently, residual solvent evaporation induces partial redissolution of crystalline grains, leading to transient structural reorganization and a corresponding decline in PL intensity. The third stage is characterized by a gradual recovery and subsequent enhancement of PL intensity, associated with Ostwald ripening-driven secondary crystal growth. In this phase, smaller crystals undergo fusion, leading to a reduction in GBs and improved crystallinity. The overall progression of the PL intensity is illustrated in Fig. 2c, where the second stage predominantly governs dynamic crystallization behavior. Notably, the target film exhibited a prolonged duration for complete perovskite conversion, confirming a retardation of crystal growth kinetics. This delayed crystallization was further corroborated in the third stage, where the target film exhibited a sustained increase in PL signal, indicative of enhanced crystal quality and a reduction in defect density. These findings underscore the efficacy of the stepwise polymerization strategy in optimizing thin-film morphology and optoelectronic properties through controlled crystallization. Furthermore, the postponed phase transition and enhanced crystallinity were further validated using in situ UV/Visible spectroscopy analysis (Fig. 2d, e). The results reveal an initial brief plateau followed by a rapid increase in absorption intensity, indicating that nucleation and crystal growth occur simultaneously from the onset of heating. Subsequently, we extracted the profiles of absorption intensity versus annealing time curves at a wavelength of 500 nm (Fig. 2f). The stepwise polymerization-modified sample exhibited an extended phase transition duration of 37.10 s, longer than that of the control film, which is 24.85 s, indicating a delayed crystallization process^35^. To monitor real-time structural evolution, in situ XRD was performed (Fig. 2g–i). The control film exhibited a persistent solvent-coordinated intermediate peak (6.55°), leading to sluggish growth of the (100) perovskite peak (~14°). Conversely, in the target film, this intermediate phase was markedly suppressed, contributing to a more direct and robust emergence of the perovskite phase. This indicates that the stepwise polymerization strategy facilitates a superior crystallization pathway by bypassing disordered intermediate stages.

The distinctly different crystallization kinetics observed in in situ PL, UV-vis, and XRD spectroscopy are expected to profoundly impact the resulting film morphology and crystallinity. Figure S8 shows the scanning electron microscopy (SEM) images of perovskite films from the view of top surface and buried interface. It is evident that the pristine perovskite film comprises numerous small grains with an average size of 309.12 nm, alongside distinct pinholes and PbI_2_ fragments distributed throughout the surface (Fig. S8a). In striking contrast, the perovskite film subjected to the stepwise polymerization strategy, which enhances crystallization and wettability, exhibited significantly enlarged and more uniform grain sizes (average size: 551.71 nm), accompanied by pinhole-free morphology and nearly eliminated PbI_2_ (Fig. S8b) relative to the control sample. In addition, SEM images of perovskite films with varying BFDGE concentrations (0.1, 0.2, 0.3, 0.4, and 0.5 mg/ml), revealed corresponding average grain sizes of 320.33 nm, 359.14 nm, 402.39 nm, 551.71 nm, and 394.72 nm, respectively (Fig. S9). These results indicate that BFDGE (0.4 mg/ml) is the optimal amount for perovskite systems, and this fixed doping concentration was consequently adopted for all subsequent experiments. Moreover, to verify the scalability of the stepwise polymerization strategy, large-area perovskite films (5 × 5 cm^2^) from both control and treated samples were sectioned into nine parts, with five representative regions selected for SEM characterization (Fig. S10a). Notably, the target perovskite films exhibited larger grains and more homogeneous surface morphology, whereas pronounced pinholes and surface inhomogeneity were observed across different regions of control perovskite film (Fig. S10b). Therefore, the proposed stepwise polymerization strategy exhibits considerable potential in modulating crystallization processes and facilitating scalable fabrication of high-quality perovskite films. In addition, atomic force microscopy (AFM) and Kelvin probe force microscopy (KPFM) measurements were conducted to further investigate the morphologies and surface potential distribution of the perovskite films. Figures S11a and S11b illustrate that stepwise polymerization strategy reduced the root mean square (RMS) roughness of the perovskite film from 23.6 to 14.3 nm, promoting better interfacial contact with the electron transport layer (ETL) and facilitating charge extraction^34,36^. Furthermore, KPFM measurements revealed a lower surface potential distribution (110.3 mV) and a narrower potential distribution in the target film compared to the control (420.2 mV) counterpart, as depicted in Fig. S11c–f, indicating that BEM incorporation contributes to a more n-type behavior and higher homogeneity characteristic of the perovskite surface^37^. Additionally, the target film exhibits a notably reduced potential difference between the grain boundaries and the grains (Fig. S11g, h). Normally, the presence of charged defects such as undercoordinated Pb^2+^ at GBs would hinder intergranular charge transfer due to electrostatic interactions and charge localization, leading to charge accumulation and recombination at the interface^38^. For the perovskite films modified by stepwise polymerization, the smaller surface potential difference implies reduced defect density and a weaker energy barrier between grains, which will improve the charge transfer efficiency in adjacent charge-selective contact layers (Fig. S11i).

To further elucidate the crystal manipulation effect of stepwise polymerization strategy on perovskite films, we performed X-ray diffraction (XRD) and grazing incidence wide-angle X-ray scattering (GIWAXS) analyses. The XRD patterns of control, pure IPDA-treated, pure BFDGE-treated, and target perovskite films are shown in Fig. S12. The target perovskite film exhibited enhanced crystallinity, as evidenced by a sharper diffraction peak at 14°, corresponding to the (100) plane, along with a narrower full width at half maximum (FWHM)^39^. Additionally, a notable reduction or disappearance of the peaks associated with *δ*-FAPbI_3_ at 11.5° and PbI_2_ at 12.7° were observed in the perovskite film modified by the stepwise polymerization strategy, compared to the control, and films treated solely with IPDA or BFDGE, suggesting the in situ stepwise polymerization between BFDGE and IPDA contributes to the formation of a high-quality perovskite phase. Figure S13a–d displays the two-dimensional (2D) GIWAXS patterns of the perovskite films treated with different agents. All the samples showed similar scattering rings at *q* = 0.86, 0.98, and 1.39, 1.7, and 1.98 Å^−1^, which can be assigned to the PbI_2_, (100), (012), (111), and (200) lattice planes, respectively^20^. Typically, the scattering ring attributed to the (100) plane in the target perovskite film appeared brighter and more sharply defined than those of the control and single-additive films, evidenced by the detailed integration along the out-of-plane (*q*_*z*_) direction, in which the target perovskite film revealed an increased (100) peak intensity (Fig. S13e). Given that the (100) facet is associated with enhanced carrier mobility, reduced trap state density, and lower exciton binding energy, it is conducive to improve the photovoltaic performance of the corresponding devices^35^. These results indicate that the in situ stepwise polymerization process between BFDGE and IPDA can assist the perovskite film crystallization, corroborating the results obtained from XRD and SEM analyses.

### Formation of the BEM for mechanically robust perovskite films

**Fig. 3:**
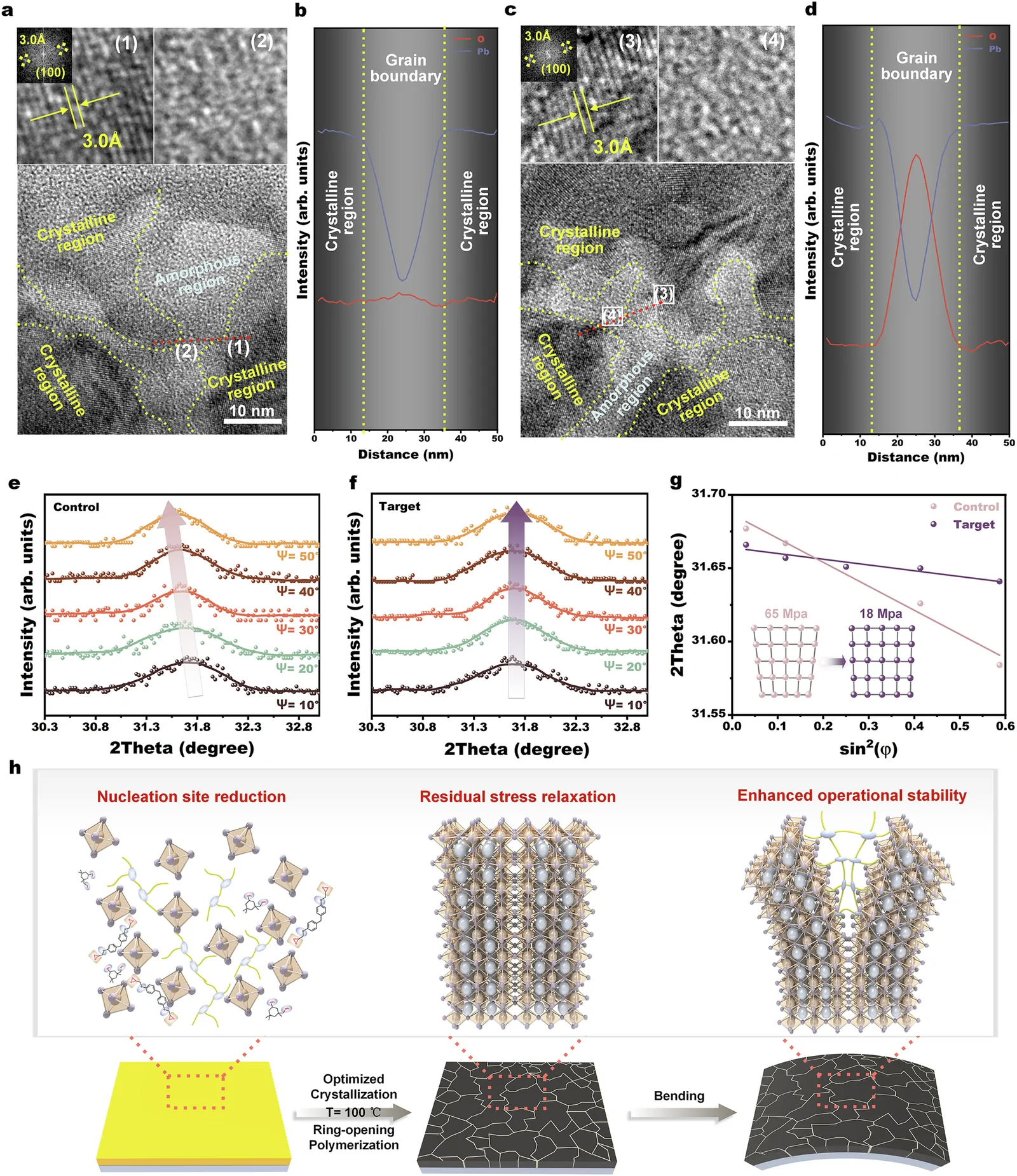
Distribution of the BEM and mechanical performance of perovskite films. **a**, **b** Magnified HRTEM images of control (inset: highlighted regions (1) and (2)) and EDX elemental line scan profiles for the red dashed line shown in (**a**). **c**, **d** Magnified HRTEM images of target perovskite films (inset: highlighted regions (3) and (4)) and EDX elemental line scan profiles for the red dashed line shown in (**c**). **e**, **f** GIXRD spectra at a 0.5° incidence angle for different perovskite films, with Gaussian fitting of the diffraction peaks. The direction of the arrows represents the peak position variation of perovskite thin films at different instrumental angles (*ψ*), **e** control, **f** target perovskite films. **g** Corresponding linear fit of 2*θ*-sin^2^*φ*. **h** Schematic of the stepwise polymerization strategy-assisted crystal regulation and stability enhancement.

To elucidate the microstructural integration of BEM within the perovskite film, high-resolution transmission electron microscopy (HRTEM) was performed focusing around the GBs regions, as indicated by the representative alternating crystalline and amorphous regions in Fig. 3a (control) and Fig. 3c (target). Notably, some specific zones delineated by a white solid line framework were selected to study, where regions (1) and (3) represent the crystalline region, whereas amorphous regions (2) and (4) indicate the GBs. The inverse fast Fourier transform analysis of the amplified HRTEM images demonstrated that the crystalline regions (highlighted regions (1) and (3)) were characterized by an interplanar spacing of 3.0 Å, which matched well with the (100) reflection of the α-phase perovskite, implying that the BEM is predominantly excluded from the perovskite lattice during crystallization^19,20^. Furthermore, energy-dispersive X-ray spectroscopy (EDX)-based depth profiling was conducted along the red dashed lines in Fig. 3a, c to determine the composition of the amorphous regions (regions (2) and (4)), with Pb selected as the representative perovskite phase and O as an indicator of BEM. As shown in Fig. 3b, the control sample exhibited a marked reduction in Pb signal intensity across regions (1) and (2) over a width of approximately 22 nm, revealing a spatial separation between adjacent perovskite grains and a lack of detectable oxygen signal. In contrast, the BEM-containing perovskite film exhibited a pronounced variation in O intensity across the GBs (Fig. 3d). These findings reveal that the BEM predominantly segregates into the amorphous regions and attached to the perovskite grains through coordination and hydrogen bonds, which are suitable to serve as a barrier and passivation agent to isolate moisture, oxygen and inactivate common defects^40^.

Considering the disparity in thermal expansion coefficients between the flexible substrate and perovskite films, as well as the mismatched ionic radius between the organic cation FA^+^ and anion [PbI_6_]^4−^, residual strain within the perovskite lattice is inevitable, which tends to result in poor mechanical integrity and inferior PCE^39^. To investigate the influence of BEM distributed along the GBs on the residual strain of the 3D perovskite lattice, we performed grazing-incidence X-ray diffraction (GIXRD) analyses combined with *2θ-*sin^2^*φ* fitting method (Supplementary Note S1). Notably, the X-ray incidence depth could be realized by varying the incidence angle and the final XRD curves were obtained by adjusting the instrument tilt angle *ψ* (10°, 20°, 30°, 40°, 50°) at a fixed diffraction angle (2*θ*). The (012) crystallographic plane oriented around 31.6° was selected for strain analysis due to its high multiplication factor, reliable mitigation of orientation effects, and representative grain information^41^. As illustrated in Fig. 3e, f, increasing *ψ* from 10° to 50° resulted in a shift of the (012) diffraction peaks of the control toward lower diffraction angles, indicating an increase in the *d*_(012)_ spacing with depth, and the perovskite film underwent enhanced tensile stress. Conversely, the perovskite film treated with BEM showed negligible peak shifts, attributed to the distribution characteristics of BEM and its interaction with the perovskite. Assuming a quasi-isotropic stress state in the perovskite films, the relationship between 2*θ* and sin^2^*φ* was fitted, as the slope of the fitted line could reflect the residual stress value. The residual tensile stress of perovskite films was estimated to be 65 (control) and 18 MPa (target), respectively (Fig. 3g), indicating that the incorporation of BEM effectively relieved the residual tensile stress. To further study the mechanical properties of the modified films, peak force quantitative nanomechanical AFM (PFQNM-AFM) was conducted to characterize the Young’s modulus (Fig. S14). As expected, the target film displayed a Young’s modulus of 2.11 GPa, which was notably smaller than that of control (6.77 GPa). This proves that the cross-linked BEM serves as a mechanical “scaffold” to buffer strain and relax stress, which in consistent with GIXRD observations.

Drawing upon the preceding investigation, a possible schematic illustrating the stepwise polymerization strategy-assisted crystallization regulation and stability optimization is presented in Fig. 3h. During the crystallization process of perovskite, the Pb-I framework ([PbI_6_]^4−^) serves as the starting point, forming nanoscale nucleation centers in the precursor solution^42,43^. Upon introducing the BFDGE and IPDA mixture into the perovskite precursor solution, it tends to coordinate with Pb^2+^ to modulate the Pb-I structure. This interaction is anticipated to optimize the phase transition process and reduce the density of nucleation sites of perovskite, thus regulating the crystallization process of perovskite on both rigid and flexible substrates. Dynamic light scattering analysis confirmed that the colloidal particles in the control precursor solution exhibited a size distribution ranging approximately from 1.2 to 364.4 nm, whereas this value increases to 59.1 to 768.5 nm with the introduction of BFDGE + IPDA mixture (Fig. S15), indicating modified precursor aggregation. During thermal annealing, the ring-opening polymerization of BFDGE and perovskite crystal growth could be simultaneously triggered, yielding a polymer network predominantly distributed along GBs that fills the interstitial spaces between adjacent grains. Interestingly, the incorporated hydrophobic polymer also relieves the residual tensile strain, which alleviates the lattice mismatch and, in turn, enhances the mechanical durability of the perovskite film. To validate this hypothesis, the fabricated f-PSCs were subjected to 5000 bending cycles at a radius of 5 mm. As shown in Fig. S16, the performance of the control device declined sharply during the bending process, retaining only 69% of its initial PCE after 5000 bending cycles. In contrast, the target f-PSC exhibited excellent bending tolerance and maintained 90% of its original PCE. Furthermore, the top-view SEM images of perovskite films post-bending (Fig. S17) revealed severe cracks formation in the control counterpart, indicative of poor bending resistance, while the BEM-modified film remained intact. This can be attributed to the flexible nature of organic elastomer BEM localized at the GBs. Consequently, constructing BEM along the GBs contributes to a substantial improvement both in the mechanical and moisture stability of the perovskite films.

### Carrier dynamics and nonradiative recombination process

**Fig. 4:**
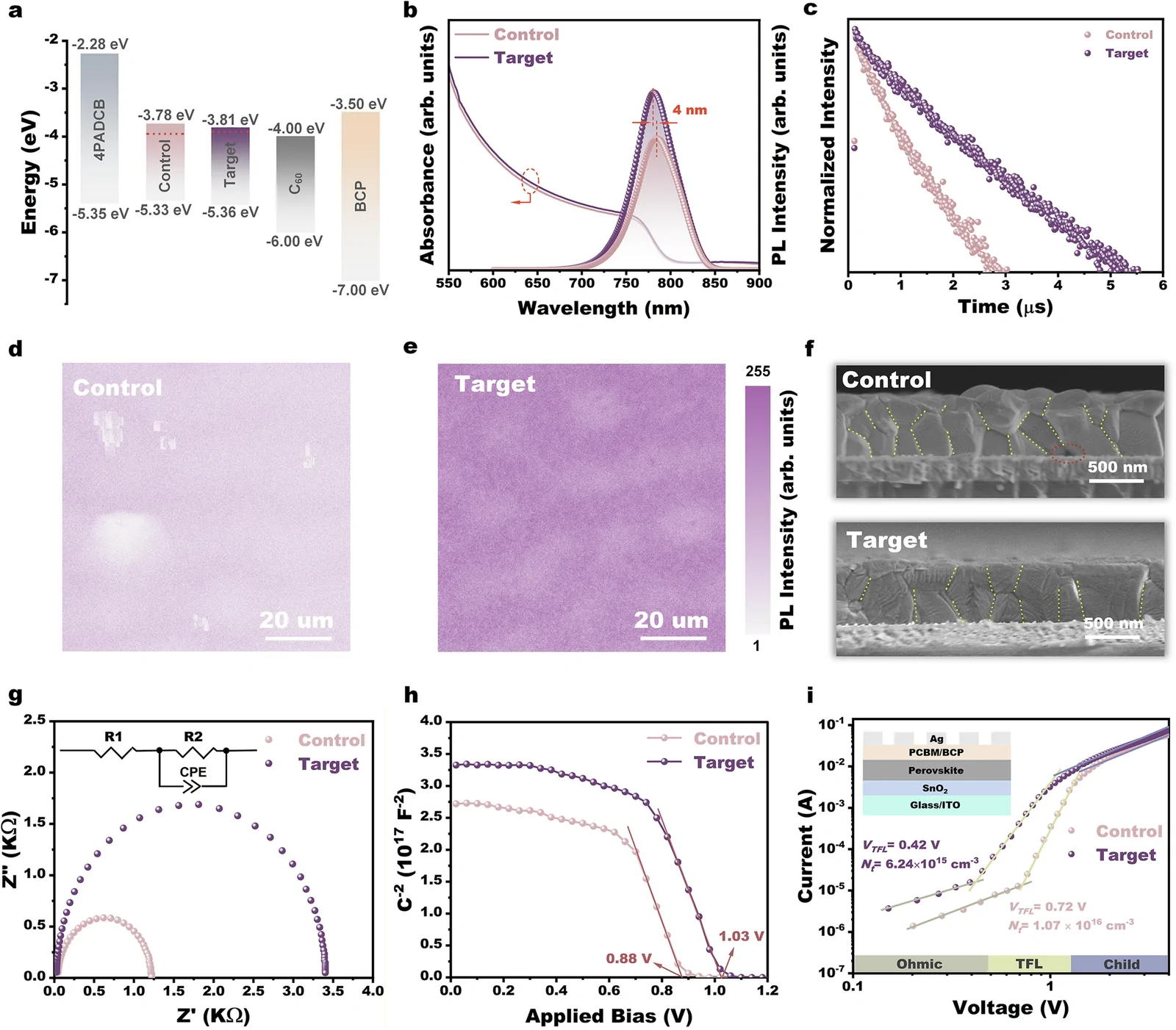
Carrier dynamics and nonradiative recombination process. **a** Energy level diagram for PSCs. **b** UV-vis absorption spectra and the steady-state PL spectra of perovskite films. **c** TRPL decay profiles measured from perovskite bottom side. **d**,** e** PL mapping patterns collected from the buried interface for the control (**d**) and target (**e**) perovskite films. **f** Cross-sectional SEM images of the perovskite films. **g** Nyquist plots of devices, inset: equivalent circuit diagram for electrochemical impedance analysis. **h** Mott-Schottky plots of devices. **i** Dark *J*–*V* curves of the SCLC measurements for the electron-only devices, inset: device architecture for SCLC testing.

Regarding carrier extraction and transport, rational energy level alignment is of great significance. Accordingly, the ultraviolet photoelectron spectroscopy (UPS) was employed to determine the energy levels of perovskite films. As illustrated in Fig. S18, the Fermi level (*E*_*F*_) shifted from −3.96 (control) to −3.89 eV (target), with corresponding valence band (*E*_*V*_) values of −5.33 and −5.36 eV, respectively. These results are in agreement with the results obtained from KPFM measurements. Moreover, according to the estimated bandgap of perovskite adsorption layer in Fig. S19, the conduction band (*E*_*C*_) value can be calculated, and the specific energy level alignment is shown in Fig. 4a. Notably, the BEM induces an N-type doping effect through its Lewis-basic N and O functional groups, which donate electron density to surface defects (Pb^2+^ or V_I_). This leads to an increased electron concentration and a consequent shift of the *E*_F_ toward the conduction band, which benefits a more favorable rational energy level alignment, facilitates electron transport, and mitigates recombination losses^44^.

In addition, to investigate the carrier transfer and recombination kinetics, we determined steady-state photoluminescence (PL) and time-resolved PL (TRPL) spectroscopy of the under researched perovskite films. As shown in Fig. 4b, the fluorescence emission intensity of the perovskite film modified by stepwise polymerization strategy was substantially higher than that of the control sample, and the target perovskite film displayed a PL emission peak at ~700 nm, along with a slight blue shift (from 784 to 780 nm), suggesting that defect-assisted nonradiative recombination can be effectively suppressed by the presence of BEM^45^. Additionally, the ameliorative crystal quality contributes to enhanced light harvesting efficiency, as supported by the ultraviolet-visible (UV-vis) absorption spectra. TRPL decay curves measured from the perovskite bottom side show a carrier lifetime of 1.9 μs for the target sample, ~1.58 times longer than that of the control (Supplementary Note S2), indicating effectively suppressed nonradiative recombination in the perovskite layer^46^ (Fig. 4c, Table S2). Furthermore, PL mapping patterns were performed to visually access the potential of stepwise polymerization strategy in mitigating the defect-assisted nonradiative recombination across large-area perovskite films. Confocal microscopy results (Fig. 4d, e) reveal micro-scale heterogeneity in the control film, with brighter regions corresponding to lower PL intensity, indicative of higher defect densities acting as nonradiative recombination centers. In contrast, the BEM-regulated perovskite film exhibits a more uniform PL intensity across the same spatial scale. This observation demonstrates that the stepwise polymerization strategy results in superior film quality with enhanced homogeneity, which holds promise for manipulating interface recombination of large-area perovskite films^47^.

Subsequently, cross-sectional SEM analysis was performed to reveal the carrier transport behavior. As shown in Fig. 4f, the control films exhibited voids at the buried interface of the perovskite/HTL, characterized by small grains and an irregular grain boundary distribution, which can be attributed to the excessively rapid crystallization rate as well as the volatilization of residual solvents^48^. Conversely, the grains of the target film were substantially larger, with any observable voids at the buried interface, and the grain boundaries were predominantly oriented vertically along the longitudinal direction. The presence of large, perpendicularly oriented crystal grains in the stepwise polymerization-modified perovskite film are beneficial to the vertical charge carrier diffusivity, as vertically aligned GBs facilitate directional charge transport, thereby enhancing collection efficiency^49^. Furthermore, electrochemical impedance spectroscopy (EIS) was conducted to evaluate the charge recombination dynamics of devices. Figure 4g shows the Nyquist plots of f-PSCs, with the equivalent circuit diagram depicted in the inset. As summarized in Table S3, the BEM-modified devices exhibited a reduced series resistance (R_1_), which contributes to an enhanced fill factor (FF) owing to the presence of vertically oriented crystal grains in the perovskite films. Meanwhile, the recombination resistance (R_2_) of BEM-modified devices was calculated to be 3377 ohms, considerably higher than that of control (1173 ohms) devices, signifying that nonradiative recombination losses can be effectively suppressed^50^. The built-in potential (*V*_bi_) was characterized by Mott-Schottky measurements. Figure 4h demonstrates that the *V*_bi_ of devices increased from 0.88 to 1.03 V following BEM modification. An elevated *V*_bi_ promotes more effective charge separation and transfer to the respective charge transport layers, which can result in improved open-circuit voltage *V*_OC_ and decreased leakage current^51^. In addition, the trap density of perovskite films was quantified by the space-charge-limited current (SCLC) analysis. Figure 4i presents the dark current-voltage curve of electronic-only devices, and the trap density was calculated using the methodology outlined in Supplementary Note S3, and summarized in Table S4. Specially, the trap density (*N*_*t*_) decreased from 1.07 × 10^16^ cm^−3^ for the control film to 6.24 × 10^15^ cm^−3^ for the BEM-treated film, confirming the effective defect passivation effect on perovskite film with BEM treatment. Last, the relationship between the *V*_OC_/short-current density (*J*_SC_) and light intensity was investigated to evaluate the charge recombination kinetics of the devices (Supplementary Note S4), with findings summarized in Table S5. Figure S20a illustrates slopes of 1.43 and 1.29 K_B_T/q for the control and BEM-treated devices, respectively, suggesting reduced defect-assisted recombination upon BEM incorporation. Figure S20b shows the exponential coefficient (*α*) values for the control and target devices, which were 0.972 and 0.998, respectively. The α value approaching unity for the BEM-treated device suggests more efficient extraction and transport of photogenerated carriers at the interface of f-PSCs. Consequently, these results discussed above provide compelling evidence that the defects within perovskite films can be effectively passivated with the assistance of stepwise polymerization strategy, thereby contributing the reduction in nonradiative recombination losses and enhancing the overall device performance.

### Photovoltaic performance and stability of small-area devices

**Fig. 5:**
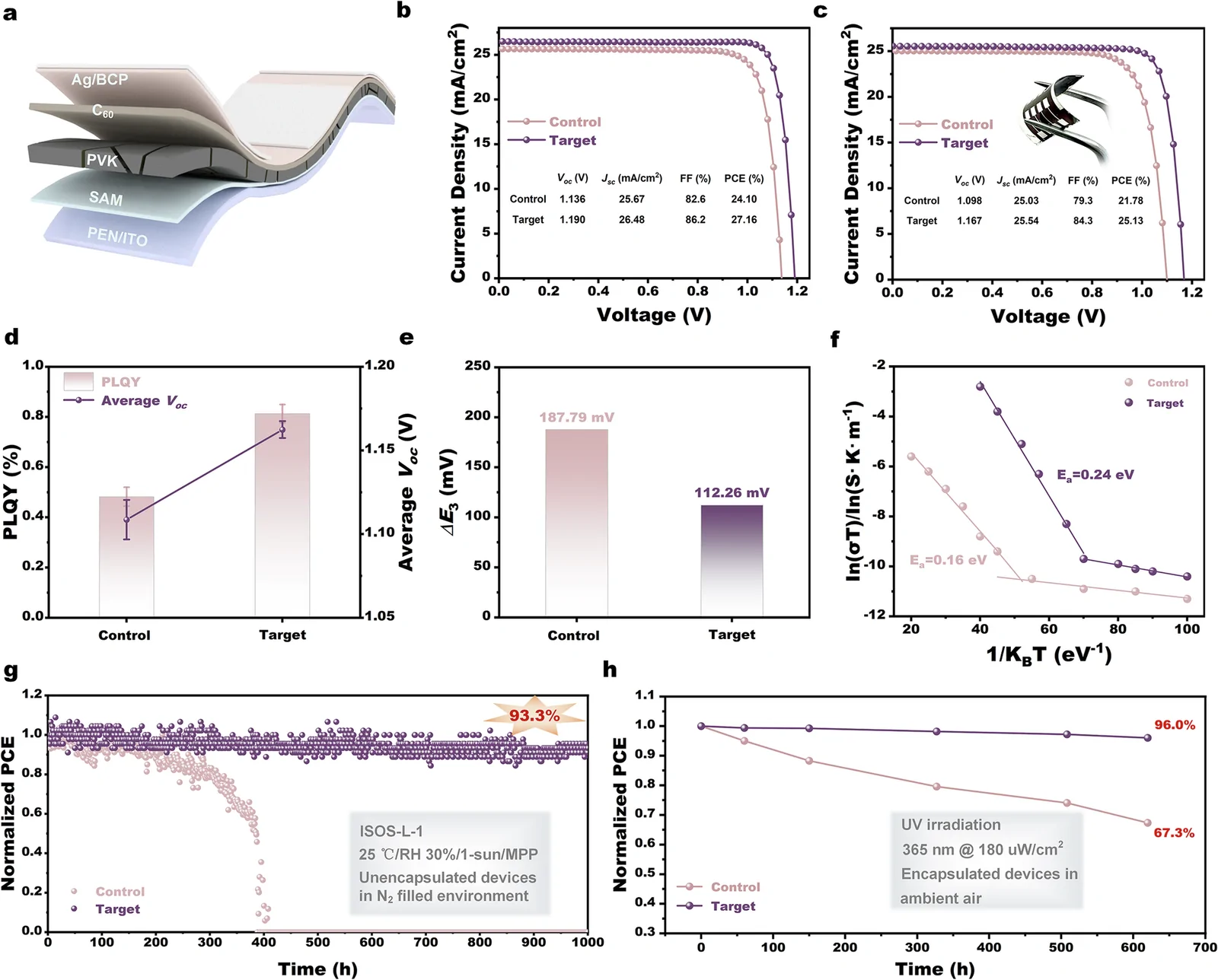
Photovoltaic performance and stability of small-area devices. **a** Device structure of f-PSCs. PVK represents the perovskite layer. **b** *J*–*V* curves of rigid PSCs. **c** *J*–*V* curves of f-PSCs, inset: photograph of the f-PSCs. **d** Comparison of the PLQY values and the measured average *V*_*OC*_ of f-PSCs. The sample size (*n*) corresponding to the error bars in (**d**) is *n* = 3. **e** Energy loss values of devices. **f** Temperature-dependent conductivity of perovskite films. **g** Stability of devices during MPP tracking measurement with 100 mW cm^−2^ LED continuous illumination. **h** UV light irradiation stability measurements of encapsulated PSCs under 365 nm UV light (180 μW/cm^2^) in air from the glass side.

Encouraged by the enhanced crystallization quality, improved surface morphology, and optimized energy level alignment, as well as the effective defect passivation of the perovskite afforded by the proposed stepwise polymerization strategy, we fabricated p-i-n planar PSCs with the configuration of glass/ITO/SAM/perovskite/C60/BCP/Ag (Fig. 5a), where the SAM denotes (4-(7H-dibenzo[c,g]carbazol-7-yl)butyl)phosphonic acid (4PADCB), and BCP is bathocuproine. The current-voltage (*J*–*V*) characteristics were determined under simulated AM 1.5 G solar illumination (100 mW/cm^2^). Figure 5b shows the *J*–*V* curves of the champion rigid PSCs obtained in the reverse scan, the target device with BEM exhibited a *V*_OC_, *J*_SC_, and FF of 1.190 V, 26.48 mA/cm^2^, and 86.2%, respectively, yielding a remarkable PCE of 27.16%, notably higher than that of control counterpart (24.10%). Furthermore, pure BFDGE or IPDA alone yielded inferior performance (Fig. S21), highlighting the necessity of the stepwise polymerization strategy. Moreover, a third-party certification organization validated stepwise polymerization-modified device, with certified PCE values of 26.87% (Fig. S22). In addition, f-PSCs were prepared to evaluate the substrate-matrix dependence, as expected, the stepwise polymerization strategy endowed the f-PSCs with a *V*_OC_*, J*_SC_, and FF of 1.167 V, 25.54 mA/cm^2^, and 84.3%, respectively, achieving an outstanding PCE of 25.13%. In contrast, the control device attained a PCE of only 21.78% with a *V*_OC_ of 1.098 V, *J*_SC_ of 25.03 mA/cm^2^ and FF of 79.3% (Fig. 5c). Therefore, the stepwise polymerization strategy can weaken the substrate dependence of the perovskite film, which can be attributed to the improved wettability and crystallinity. Notably, the PCE enhancement following the incorporation of BEM primarily stems from a substantial increase in FF, which can be ascribed to the enhanced electron extraction and transfer capabilities, facilitated by a pronounced reduction in series resistance and optimized energy level alignment. Additionally, the *V*_OC_ was remarkably increased as well, presumably due to suppressed charge recombination process resulting from the effective chemical passivation of defects at GBs^52^. These results underscore the critical role of stepwise polymerization strategy in enabling efficient PSCs. Moreover, the hysteresis index (HI) was calculated by Supplementary Note S5, and the results were summarized in Fig. S23. It is noteworthy that a lower HI was observed in the target devices, indicating improved carrier extraction and transport by BEM modification.

To verify the reliability of *J*–*V* measurements, the stabilized power output (SPO) was measured under MPP tracking. As shown in Fig. S24, the target rigid and flexible PSCs delivered steady-state power outputs of 26.12% and 24.64%, respectively, during 300 s of continuous tracking at their maximum power points. In addition, Figs. S25 and S26, along with Tables S6 and S7, present the photovoltaic parameters of 30 individual rigid and flexible devices. Devices modified with the stepwise polymerization strategy exhibited narrower distributions and enhanced photovoltaic performance, suggesting enhanced reproducibility attributable to this approach. Moreover, the external quantum efficiency (EQE) spectra were determined, as shown in Fig. S27. The integrated *J*_SC_ values of 24.46 and 24.85 mA/cm^2^ for the control and target f-PSCs, respectively, corresponded well with the *J*_SC_ derived from *J–V* measurements. Notably, to further understand the enhancement in *V*_OC_, photoluminescence quantum yield (PLQY) measurements for the glass/ITO/perovskite device stacks were performed to further investigate carrier recombination dynamics. As presented in Fig. 5d, statistical analysis reveals that the target perovskite film exhibited a slight enhanced average PLQY of 0.81%, versus the control (0.48%) sample. However, the average *V*_OC_ measured from *J*–*V* scans was 1.163 V for the target device, exceeding the control’s 1.109 V by 54 mV. Therefore, the nonradiative recombination at the interface may be a limiting factor in the performance of control devices, implying the proposed stepwise polymerization strategy plays a significant role in manipulating interfacial recombination^33^. Likewise, we conducted electroluminescence quantum efficiency (EQE_EL_) analysis. The nonradiative energy loss was calculated using the following equation: Δ*E*_3_ = −*kT*/*qln*(EQE_EL_), where *k* denotes the Boltzmann constant and *T* is the temperature. As shown in Fig. 5e, the EQE_EL_ of the target device was 1.30%, corresponding to a Δ*E*_*3*_ value of 112.26 mV. This value is much lower than that of the control counterpart (187.79 mV), further suggesting that increased *V*_OC_ stems from the reduced nonradiative recombination losses, which can be attributed to the effective passivation effect of BEM.

Furthermore, considering operational stability is a critical bottleneck for the practical applications of f-PSCs, we systematically monitored the long-term and environmental stability of the devices. To evaluate long-term chemical stability under UV exposure, encapsulated devices were subjected to continuous UV irradiation (365 nm, 180 μW/cm^2^) for 630 h. As shown in Fig. 5h, the encapsulated devices treated with BEM retained an impressive 96.0% of their initial efficiency after 630 h irradiation, while the control samples plummeted to 67.3%. This demonstrates that the encapsulated polymer network effectively suppresses UV-induced perovskite decomposition and is chemically stable under harsh radiation. Recognizing the high sensitivity of perovskite materials to moisture, the water contact angles of perovskite films were initially analyzed. As presented in Fig. S28, the water contact angles of perovskite films notably increased from 48.6° (control) to 70.8° (target). This phenomenon is benefited from the hydrophobicity of BEM distributed along the GBs, which effectively impedes moisture penetration into the film bulk and consequently inhibits hydration reactions thereafter. Moreover, the operational stability of f-PSCs was evaluated by conducting MPP tracking under continuous 1-sun illumination (AM 1.5 G, 100 mW/cm^2^) in a nitrogen-filled atmosphere (Fig. 5g). The target f-PSC exhibited superior operational durability, retaining 93.3% of its original PCE after 1000 h of continuous output, whereas the control device degraded completely within 400 h. Specially, the operational stability is intrinsically linked to the ion migration within perovskite films^30^. Unlike charge carrier conduction, which predominates at lower temperatures, ionic conductivity becomes significant at elevated temperatures due to the energy required to activate ion migration. To investigate this relationship, temperature-dependent conductivity analyses were conducted to determine the activation energy (*E*_a_) of both control and target films. As expected, the perovskite film modified by BEM exhibited an increased *E*_a_ relative to the control counterpart (Fig. 5f), indicating suppressed ion migration and consequently reduced thermal degradation. Additionally, time-of-flight secondary ion mass spectrometry (ToF-SIMS) measurements were performed to analyze ion migration in samples after 100 h of light soaking, where PbI^-^ was selected as the representative signal of the perovskite phase, while O^-^ was used to represent the BEM. Figure S29 shows a uniform O^-^ distribution throughout the target film, confirming the formation of a 3D bulk scaffold at grain boundaries, whereas no significant signal is detected in the control. Importantly, the control device exhibited a notable I^-^ ion diffusion into the electron transport layer and the Ag electrode, which may accelerate electrode corrosion and device degradation. In comparison, I^-^ ion migration was negligible in the target device, suggesting the photo-induced ion migration can be effectively inhibited by BEM distributed along the GBs. Furthermore, to explore the specific microscopic alterations under accelerated aging conditions, the XRD patterns and aging photographs of the different perovskite films were continuously tracked over a 10-day period under 1-sun illumination at approximately 50% relative humidity (RH) in ambient air. Phase stability was quantified by the intensity ratio of α-phase (100) peak at 14° to the δ-phase peak at 11.5°. As shown in Fig. S30, phase deterioration can be observed through a noticeable transition from the black α-phase to the light yellow δ-phase. The control perovskite film degraded rapidly, as indicated by the notable reduction of the α-phase after 240 h of exposure. In contrast, the α-phase (100) peak ratio in the target perovskite film remains at 95.8%, demonstrating the efficacy of the stepwise polymerization strategy in enhancing moisture resistance and structural integrity.

### Scalable fabrication potential of stepwise polymerization strategy

**Fig. 6:**
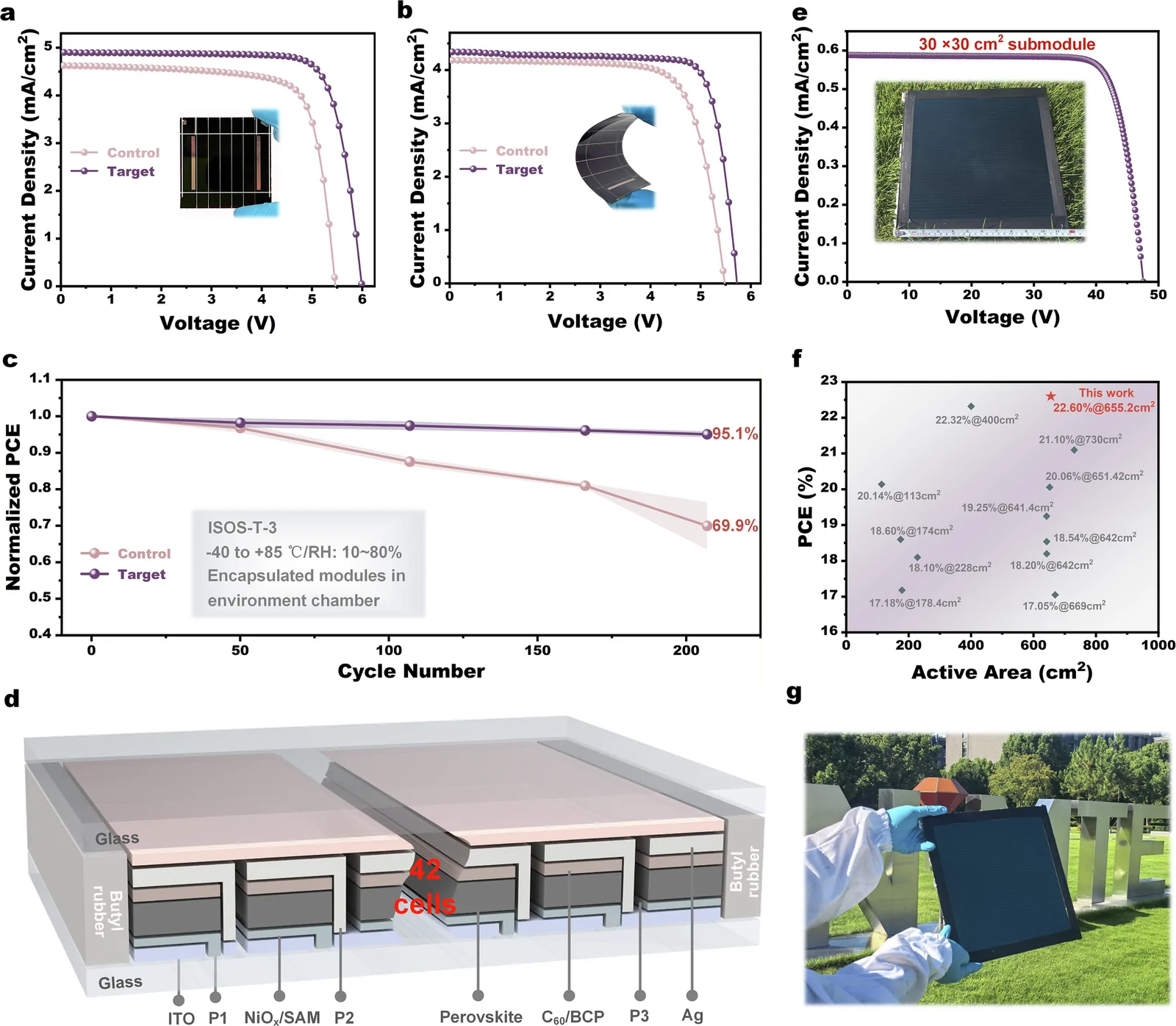
Scalable preparation potential of stepwise polymerization strategy. **a** *J–V* curves of the best-performing rigid minimodule, inset: photograph of the rigid minimodule. **b** *J*–*V* curves of the best-performing flexible minimodule, inset: photograph of the flexible minimodule. **c** Thermal cycling stability of encapsulated modules aged between −40 °C to +85 °C (ISOS-T-3). The sample size (*n*) corresponding to the error bars in (**c**) is *n* = 3. **d** The schematic diagram of 900 cm^2^ PSM with a series connection of 42 subcells. **e** *J–V* curve the champion 30 cm × 30 cm^2^ submodule with an active area of 655.2 cm^2^, inset: photograph of the submodule. **f** Summary of PCE versus area for inverted PSMs in recent years (active area over 100 cm^2^). **g** Photograph of a 30 cm × 30 cm^2^ submodule.

Encouraged by the potential of the stepwise polymerization strategy in realizing high-quality perovskite films across large-area substrates, we fabricated corresponding PSMs, with an area of 5 by 5 cm^2^ (aperture area: 10.24 cm^2^) to validate its scalable applicability. The minimodule configuration is shown in Fig. S31, each minimodule comprises five series-connected subcells, inked via precisely engineered laser-patterned P1, P2, and P3 channels, with a geometric fill factor (GFF) of 96.5%. Firstly, rigid PSMs were assembled to detect the performance feedback of deposited modified-perovskite precursor solution on rigid substrates. Notably, the target rigid PSMs achieved a champion PCE of 23.25%, with a *V*_OC_ of 5.99 V, a *J*_SC_ of 4.92 mA/cm^2^, and an FF of 78.9%. In contrast, the control module only delivered a much lower PCE of 19.24%, mainly due to variation in significant reductions in *V*_OC_ and FF values (Fig. 6a, Table S8). More importantly, based on the uniform and improved crystallinity on the flexible substrate, the flexible PSMs obtained a remarkable PCE of 20.59%, with a *V*_OC_ of 5.72 V, a *J*_SC_ of 4.50 mA/cm^2^, and an FF of 80.0%, substantially surpassing the control f-PSMs (17.61%) (Fig. 6b, Table S9). Noteworthily, the moderate *J*_SC_ mainly constrained by the transmittance of flexible substrates. Moreover, as illustrated in Fig. S32, the target champion rigid PSM maintained a stable PCE of 22.34% under MPP tracking at a bias voltage of 4.921 V, corresponding to a photocurrent of 4.54 mA/cm^2^. Likewise, the f-PSM also demonstrated a comparable stabilized efficiency of 20.00%. In addition, the statistical analyses of photovoltaic performance for both control and target samples derived from 15 individual modules, are shown in Figs. S33, S34 and Tables S10 and S11, confirming the excellent reproducibility of modules manufactured using this strategy. Collectively, these results validate the scalable fabrication potential of the proposed stepwise polymerization strategy.

To further deep insight the mechanisms underlying enhanced scalability, the drying process of perovskite ink droplets on 4PADCB-modified substrates was monitored (Fig. S35a, b). During solvent evaporation, the droplet volume decreased, causing the contact line (the edge of the droplet) to move, with the coverage defined as the ratio of the droplet’s projected area on the substrate during drying to its initial area. As a consequence, the coverage of pristine perovskite solution reduced to 89.3%. In contrast, the non-wetting problem was solved when with the assistance of the stepwise polymerization strategy. The coverage of the droplet remained 96.8% during the whole drying period (Fig. S35c). The contact line was strongly fixed to the substrate, indicating the formation of hydroxyl-functionalized oligomers acts as a molecular “anchor”, notably enhancing the interfacial affinity and preventing film retraction during large-area deposition (Fig. S35d). Subsequently, PL mapping measurements were conducted on perovskite films deposited on 5 by 5 cm^2^ substrates. A markedly higher PL intensity and homogeneity across large-area perovskite film was observed in the target sample (Fig. S36), which contributes to improved reliability and mitigates the performance loss when scaling up PSCs. Additionally, Fig. S37 displays the electroluminescence (EL) imaging of both control and target samples, highlighting irregular luminescence with localized non-emissive spots in the control module caused by inferior coverage and inhomogeneous coating. Specifically, the operational stability of encapsulated PSMs at MPP tracking was characterized, as shown in Fig. S38. Benefiting from the hydrophobic nature and strain-relaxation effects of BEM distributed along the GBs, the target PSM retained 91.1% of its initial PCE after 1000 h of continuous output. Moreover, following the ISOS-T-3 protocol, modules were cycled between −40 °C and 85 °C. The target modules retained 95.1% of their initial PCE after 200 cycles, notably outperforming the control devices, which retained only 69.9% on average (Fig. 6c). These results collectively underscore the superiority of the stepwise polymerization strategy in enabling stable and scalable f-PSMs. Regarding the exceptional performance of stepwise polymerization-modified minimodules, we attempted to extend this strategy to submodules (Fig. 6g). Figure 6d illustrates a 30 × 30 cm^2^ PSM comprising 42 subcells via series connection. The target PSM delivers a remarkable PCE of 22.60% (active area: 655.2 cm^2^) (Fig. 6e, Table S12), which is one of the highest efficiencies for such large-scale inverted PSMs (Fig. 6f and Table S13). Likewise, statistical evaluation across 10 independently fabricated submodules confirmed the excellent reproducibility of this scalable strategy (Fig. S39 and Table S14). Notably, the performance of the champion submodule was validated by independent solar cell-accredited laboratory (National PV Industry Measurement and Testing Center), conforming a high PCE of 21.07% under reverse scan (Fig. S40). These findings collectively underscore the scalable applicability and commercial potential of the stepwise polymerization strategy for fabricating efficient and stable perovskite solar modules.

## Discussion

In summary, the stepwise polymerization strategy proposed in this study effectively tackles the longstanding challenges of limited stability and scalability in flexible perovskite solar cells (f-PSCs). The engineered combination of the BFDGE monomer and IPDA curing agent enables sequential reaction kinetics perfectly synchronized with perovskite crystallization, thereby achieving effective ink modification at ambient temperature and crystallization regulation during annealing. This approach fundamentally overcomes the issue of inferior ink wettability issue on hydrophobic substrates, a primary bottleneck for scalability, while generating a multifunctional cross-linking scaffold that encapsulates perovskite grains. Consequently, this approach leads to superior film morphology, enhanced crystallinity, and remarkable operational stability. The optimized small-area rigid and flexible PSCs achieved an impressive PCE of 27.16% and 25.13%, respectively. Crucially, the PSCs exhibited remarkable durability, maintaining over 93.3% of their initial PCE after 1000 h of continuous MPP tracking and 95.1% after 200 thermal cycles (ISOS-T-3), alongside excellent mechanical flexibility (over 90% retention after 5000 bending cycles). Furthermore, perovskite solar minimodules demonstrated high PCEs of 23.25% (rigid) and 20.59% (flexible). Notably, a large-area submodule with an active area of 655.2 cm^2^ achieved a record-breaking PCE of 22.60%, certified at 21.07%, setting a new benchmark for inverted submodules. These results highlight the potential of the stepwise polymerization strategy to enable a viable and scalable pathway toward high-performance and durable f-PSCs, making a significant step toward their commercialization and broad implementation in practical photovoltaic applications.

## Methods

### Materials

Solvents: N, N-dimethyl formamide (DMF, anhydrous, ≥99.8%) and dimethyl sulfoxide (DMSO, anhydrous, ≥99.9%) were purchased from Sigma-Aldrich (USA). Diethyl ether (DE, anhydrous, 99.7%), Absolute ethanol (General-Reagent, 99.7%), Acetone (99.5%), and isopropanol (IPA, 99.7%) were purchased from Sinopharm Chemical Reagent Co., Ltd (China).

Reagents: (4-(7H-dibenzo[c,g]carbazol-7-yl)butyl)phosphonic acid (4PADCB, ≥99.8%), Fullerene C60 (C60, ≥99.5%), Bathocuproine (BCP, ≥99.5%) were purchased from Shanghai Daeyeon Chemical Corp (China). Patterned Glass/ITO substrate, PEN/ITO substrate, Cesium iodide (CsI, 99.99%), Formamidinium iodide (FAI, 99.9%), Methylammonium bromide (MABr, 99.99%), Lead(II) iodide (PbI_2_, 99.99%), Lead bromide (PbBr_2_, 99.99%) and methylammonium chloride (MACl, 99.9%) were purchased from Advanced Election Technology Corp (China). The key feedstock bisphenol F diglycidyl ether (BFDGE) was purchased from Sigma-Aldrich Corporation, and isophorone diamine (IPDA) was purchased from Tokyo Chemical Industry Co., Ltd.

All the chemicals and reagents were purchased and used directly without any further purification.

### Solution preparation

The 1.62 M perovskite precursor solution with the composition of Cs_0.05_(FA_0.98_MA_0.02_)_0.95_(I_0.98_Br_0.02_)_3_ was prepared by fully dissolving FAI, MABr, CsI, PbI_2_, PbBr_2_ in a mixed solvent of DMF and DMSO with a volume ratio of 4:1. Moreover, for the perovskite film with BEM, 10 μL BFDGE (40 mg/mL in DMF) and 10 μL IPDA (2 mg/mL in DMF) (5 wt% of BFDGE) were added into 1 mL perovskite precursor solution. Then, the perovskite precursor solution was stirred at room temperature for 6 h.

### Device fabrication

Firstly, the patterned glass/indium tin oxide (ITO) substrates were washed with detergent, deionized water, acetone, and isopropanol in sequence for 20 min. After that, the cleaned substrates were dried by nitrogen (N_2_) flow and then treated with oxygen plasma for 5 min. The prepared devices with an architecture of Glass /ITO/4PADCB/Perovskite/C60/BCP/Ag. In terms of the preparation of rigid device, 100 μl 4PADCB (0.8 mg/ml) dissolved in absolute ethanol was spin-coated on ITO for 30 s at 3000 rpm and then annealed at 120 °C for 10 min. Subsequently, 50 μl perovskite precursor solution was deposited on 4PADCB-based substrate at 4000 rpm for 30 s, 600 μl DE as the antisolvent was added dropwise onto the center of substrate at 15 s before the end of spin-coating. The as-prepared perovskite films were immediately transferred to hotplate and annealed at 100 °C for 30 min. The aforementioned spin-coated processes were all conducted at room temperature (25–30 °C) in a N_2_-filled glove box with the contents of O_2_ and H_2_O < 0.01 ppm. Finally, 25 nm of C60 at a rate of 0.2 Å/s, 10 nm of BCP at a rate of 0.2 Å/s, and 100 nm of silver (Ag) electrode at a rate of 2.0 Å/s were thermally evaporated, respectively, under high vacuum (<1 × 10^−4^ pa). Additionally, it must note that maintaining the flatness of flexible substrate during the device fabrication process is of great significance for realizing highly efficient flexible perovskite devices. Taking this into account, glass/184 silicone elastomer (PDMS) was used as rigid support, the PEN/ITO substrate was attached on the support before the whole fabrication process, then the same spin-coating procedure was performed.

### Modules fabrication process

For the fabrication of perovskite solar modules (PSMs), the primary difference compared to small-area device fabrication is that the modules involve additional scribing steps to divide a large-area device into small-area subcells and form electrical interconnections between them. The scribing scheme includes three basic steps (known as P1, P2, and P3 scribing). The P1 pattern is etched on glass/ITO substrates by laser, a femtosecond infrared laser (1030 nm, 400 fs, 0.5 MHz, scribing speed 2000 mm/s, average power 10.063 W), and then the substrates are cleaned using the aforementioned methods. The 4PADCB/Perovskite/C60/BCP layers are subsequently deposited on the substrates. The P2 scribing step removes the BCP/C60/PVK/4PADCB layers, exposing the ITO within the P2 pattern lines (1030 nm, 400 fs, 0.5 MHz, scribing speed 2000 mm/s, average power 3.123 W). Afterward, 100 nm of Ag was deposited by thermal evaporation, covering the top of ETL and filling the P2 channels. Finally, the P3 scribing step is conducted to isolate the Ag electrode between neighboring subcells, using the same laser with 4.858 W average power and scribing speed 3000 mm/s, completing the monolithic interconnections.

### Submodules fabrication process

The 1.2 M perovskite precursor solution with the nominal composition of Cs_0.05_(FA_0.98_MA_0.02_)_0.95_(I_0.98_Br_0.02_)_3_ was prepared by dissolving FAI, MABr, CsI, PbI_2_, and PbBr_2_ in a mixed solvent of DMF and NMP (6:1 volume ratio). To accommodate the slot-die coating process conducted under ambient conditions (T: 23 °C, RH: 20%), an additional 10% MACl, 3% PbCl_2_, and 5% PbI_2_ were introduced into the precursor to enhance the crystallization quality of the perovskite film.

For the 300 × 300 mm^2^ ITO substrates, the P1, P2, and P3 scribing were conducted using the same femtosecond laser system and power/speed parameters described for the minimodules. The specific process for the submodule includes:

#### Transport layer

A NiO_x_ hole transport layer doped with 1% Mg was deposited by magnetron sputtering to a thickness of 8 nm. Subsequently, a 4PADCB layer was coated by slot-die coating using an ethanol solution with a concentration of 0.5 mg/mL, at a coating speed of 100 mm/s and a gap height of 80 μm. The film was directly annealed at 100 °C for 10 min without vacuum flash.

#### Perovskite layer

The perovskite layer was then deposited by slot-die coating at a coating speed of 80 mm/s with a gap height of 100 μm. Immediately after coating, the substrate was subjected to vacuum flash evaporation at a minimum pressure of 10 Pa for 65 s. Pre-annealing was carried out at 70 °C for 3 min, followed by a final annealing at 100 °C for 30 min.

### Characterizations of films

The Fourier transform infrared (FTIR) spectroscopy was detected by FTIR8400S (Shimadzu). The X-ray photoelectron spectroscopy (XPS) experiments were recorded on a monochromatic Al-Kα (1486.6 eV) as the radiation source (Shimadzu, Axis Supra). The Scanning Electron Microscope (SEM) (verios G4 UC) (Rimono Scientific Company, America) was performed at 2 kV to obtain the top-view and cross-sectional SEM images of perovskite films. X-Ray diffraction (XRD) measurements were performed using a D/MAX Ultima III XRD spectrometer (Rigaku) with a Cu Kα line of λ = 1.5410 Å. Grazing incident wide-angle X-ray scattering (GIWAXS) measurements were performed by the XEUSS SAXS/WAXS equipment, at an incident angle of 0.5°. The Atomic Force Microscopy (AFM) images, Kelvin Probe Force Microscopy (KPFM) images and the peak force quantitative nanomechanical atomic force microscopy (PFQNM-AFM) were captured by Bruker Dimension ICON SPM, America. The energy level was obtained by ultra-violet photoelectron spectroscopy (UPS) (R3000, Scienta) using HeI (21.2 eV) as the excitation source and ultraviolet-visible absorption spectra (UV-vis) (Lambda 950). The steady photoluminescence (PL) spectra were obtained from the Horiba jobin Yvon fluorolog-3 spectrofluorometer system with incident light at 405 nm and the time-resolved photoluminescence (TRPL) was analyzed by the FLS 980 fluorescence spectrometer equipped with a 475 nm excitation. PL mapping was measured by LEICA TCS SP5.

### Characterizations of devices

The photovoltaic performance of the device was measured under simulated solar lamp (100 mW/cm^2^, AM1.5G) provided by Newport oriel sol3A 450 W solar simulator. The *J–V* curves of devices were measured at room temperature The light source parameters are 450 W xenon lamp (oriel solar simulator), calibrated to 100 mW/cm^2^ by silicon reference cell. The effective area is 0.06 cm^2^, defined as a non-reflective mask. The scanning parameters were forward scanning (−0.2 to 1.2 V, step 0.02 V, scan rate: 0.1 V/s dwell time 50 ms) and reverse scanning (from 1.2 to −0.2 V, step 0.02 V, scan rate: 0.1 V/s dwell time 50 ms). The stabilized power output (SPO) and steady-state current density curves of the devices were measured by detecting the photocurrent density output with the biased voltage set at the MPP. The dependence of *V*_OC_/*J*_SC_ on light intensity was measured by varying the light intensity. The solar cell quantum efficiency test system (Elli Technology Taiwan) was used to measure the EQE spectra of devices with a wavelength range of 300–1100 nm. The capacitance versus frequency (C–F) curves were recorded by the 1240 A Impedance Analyzer. The Mott-Schottky curves and the impedance spectroscopy (IS) were determined with the Chenhua CHI760E electrochemical workstation. EQE_EL_ measurements were measured by applying external voltage/current sources through the PSCs with a REPS measurement instrument (Enlitech). The operational stability of the devices was also examined by monitoring the maximum power point (MPP) tracking under 1 sun illumination in an N_2_ environment (~25 °C and ~30% RH).

### Reporting summary

Further information on research design is available in the Nature Portfolio Reporting Summary linked to this article.

## Supplementary information

Supplementary Information

Reporting Summary

Transparent Peer Review file

## Source data

Source Data

## Data Availability

The data that support the findings of this study are available in the paper and Supplementary Information/Source data file. Source data are provided with this paper.
