# Supplementary material for: Stepwise polymerization enables tailored perovskite ink for scalable flexible perovskite solar cells
Source: Nat Commun. 2026 Sep 15;17:10858. doi: 10.1038/s41467-026-77703-w (PMC13672371; doi:10.1038/s41467-026-77703-w)
Supplement: Supplementary file 1 [file 41467_2026_77703_MOESM1_ESM.pdf]

Supplementary Information

Stepwise Polymerization Enables Tailored Perovskite Ink for Scalable Flexible Perovskite Solar Cells

Weifu Zhang[1,2,3 †], Zhaojin Wang[4 †], Hengyu Zhou[1 †], Wei Song[1], Chenfan Xing[1], Jiahan Xie[1], Jintao Zhu[5], Jiaming Huang[6*], Xiaowei Xu[1*], Mengjin Yang[1*], Yang Bai[4], Ziyi Ge[1,3*]*

[1] Zhejiang Provincial Engineering Research Center of Energy Optoelectronic Materials and Devices, Ningbo Institute of Materials Technology & Engineering, Chinese Academy of Sciences, Ningbo 315201, China.

[2] CAS Key Laboratory of Green Printing, Institute of Chemistry, Chinese Academy of Sciences Beijing 100190, China.

[3] Center of Materials Science and Optoelectronics Engineering, University of Chinese Academy of Sciences, Beijing 100049, China.

[4] Faculty of Materials Science and Energy Engineering, Shenzhen University of Advanced Technology, Shenzhen, China

[5] Department of Chemical and Environment Engineering, University of Nottingham Ningbo China, Ningbo 315100, China.

[6] Department of Electrical and Electronic Engineering, The Hong Kong Polytechnic University, Hung Hom, Kowloon, Hong Kong, China.

* Corresponding authors.

E-mail: songwei@nimte.ac.cn; jiaming1993.huang@polyu.edu.hk; xuxiaowei@nimte.ac.cn; yangmengjin@nimte.ac.cn; geziyi@nimte.ac.cn

[†] These authors contributed equally to this work.

Calculation Methods

Note S1: Residual stress calculation based on GIXRD results

According to Bragg's Equation and generalized Hooke's Law, the residual stress (σ) can be determined by 2θ-sin2φ method:

$$\sigma = -\frac{E}{2\,(1+\nu)}\,\frac{\pi}{180^\circ}\,\cot\theta_0\,\frac{\partial(2\theta)}{\partial(\sin^2\varphi)}$$

Where φ and E are the diffraction vector angle respective to the sample surface normal and the perovskite modulus, respectively. νis the Poisson's ratio of the perovskite, θ_0 is half of the scattering angle $2\theta_0$ (the (012) crystallographic plane oriented at near 31.6°). By linear fitting 2θ as a function of $\sin^2\varphi$, the σ could be obtained. Positive slope indicates compressive stress, and negative slope indicates tensile stress.

Note S2: Carrier recombination lifetime and exciton lifetime calculation

The carrier recombination lifetimes can be derived from time-resolved photoluminescence (TRPL), the TRPL decay curves were fitted by the bi-exponential function:

$$y\,(t) = A_1 \exp^{\frac{-t}{\tau_1}} + A_2 \exp^{\frac{-t}{\tau_2}} + y_0$$

$$\tau_{ave} = \frac{\sum A_i \tau_i^2}{\sum A_i \tau_i}$$

where, A_1 and A_2 are the relative amplitudes, τ_1 and τ_2 represent time constants for fast and slow decay lifetimes in relation to nonradiative recombination of surface traps near the GBs and radiative recombination from the bulk perovskite film, respectively.

Note S3: Analyzation of space-charge-limited current (SCLC) model

The dark *J-V* curves display three typical regions: (I) the Ohmic region at low bias region, (II) the trap-filled limited region at intermediate bias region, and (III) a trap-free SCLC region at high bias region. In the trap-filled limited region, the trap density levels are continuously filled as bias increases, and all of traps are filled until the trap-filled limit voltage (V_{TFL}). The trap density is estimated according to the equation:

$$N_t = \frac{2\varepsilon\varepsilon_0 V_{TFL}}{qL^2}$$

Where N_t is the trap density, ε is the dielectric constants of perovskite, ε_0 is the vacuum permittivity, L is the thickness of the perovskite film, and *q* the elementary charge. V_{TFL} is obtained by fitting the dark *I-V* data with the equation.

Note S4: The dependence of J_{SC} and V_{OC} of the PSCs on light intensity

The ideality factor nKT/q can be calculated by equation:

$$V_{OC} = \frac{\mathrm{nKT}}{\mathrm{q}} \ln(\mathrm{P_{light}})$$

The exponential factor α can be calculated by equation:

$$\alpha = \frac{\ln(J_{sc})}{\ln(\mathrm{P_{light}})}$$

Note S5: Hysteresis index (HI)

The *J-V* hysteresis index (HI) is defined by the following equation:

$$\mathrm{HI} = \frac{\mathrm{PCE_{Reverse}} - \mathrm{PCE_{Forward}}}{\mathrm{PCE_{Reverse}}}$$

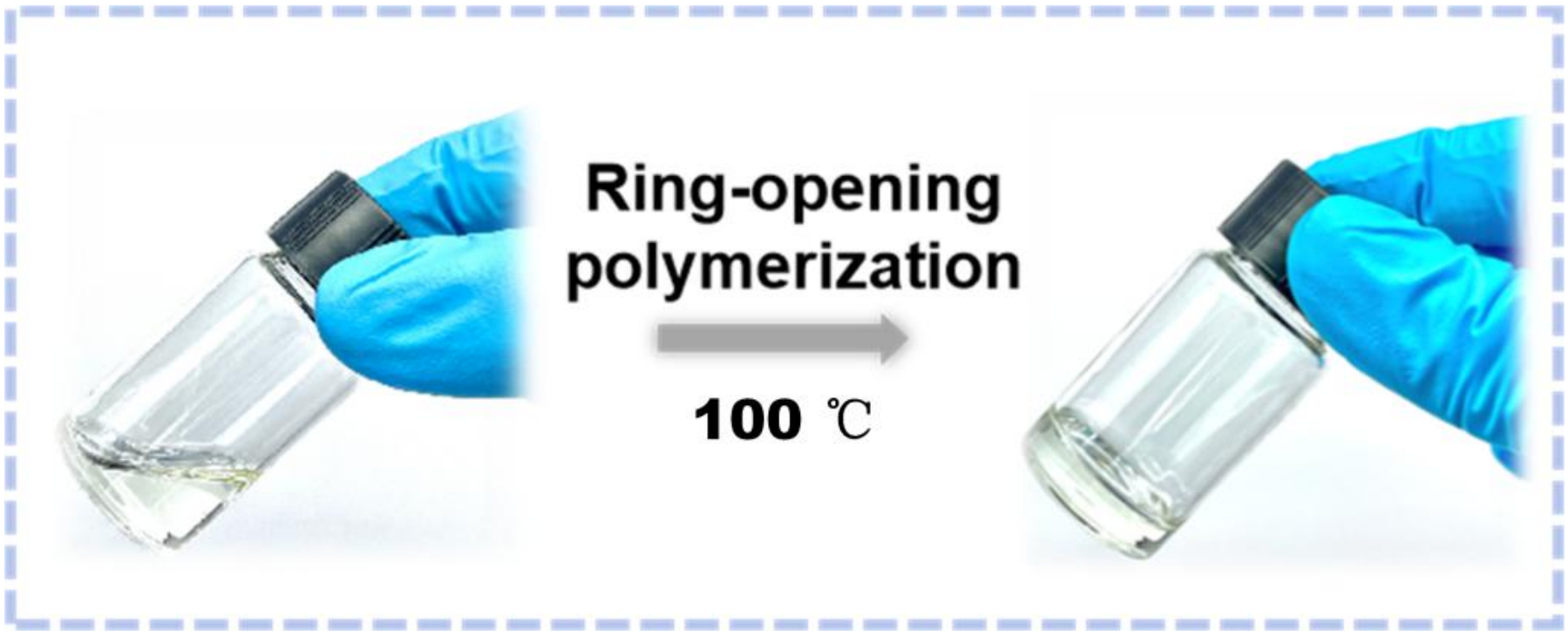

Figure S1. Crosslinking of BFDGE molecules occurred via ring opening polymerization of epoxide following a thermal treatment with IPDA as crosslinking initiator.

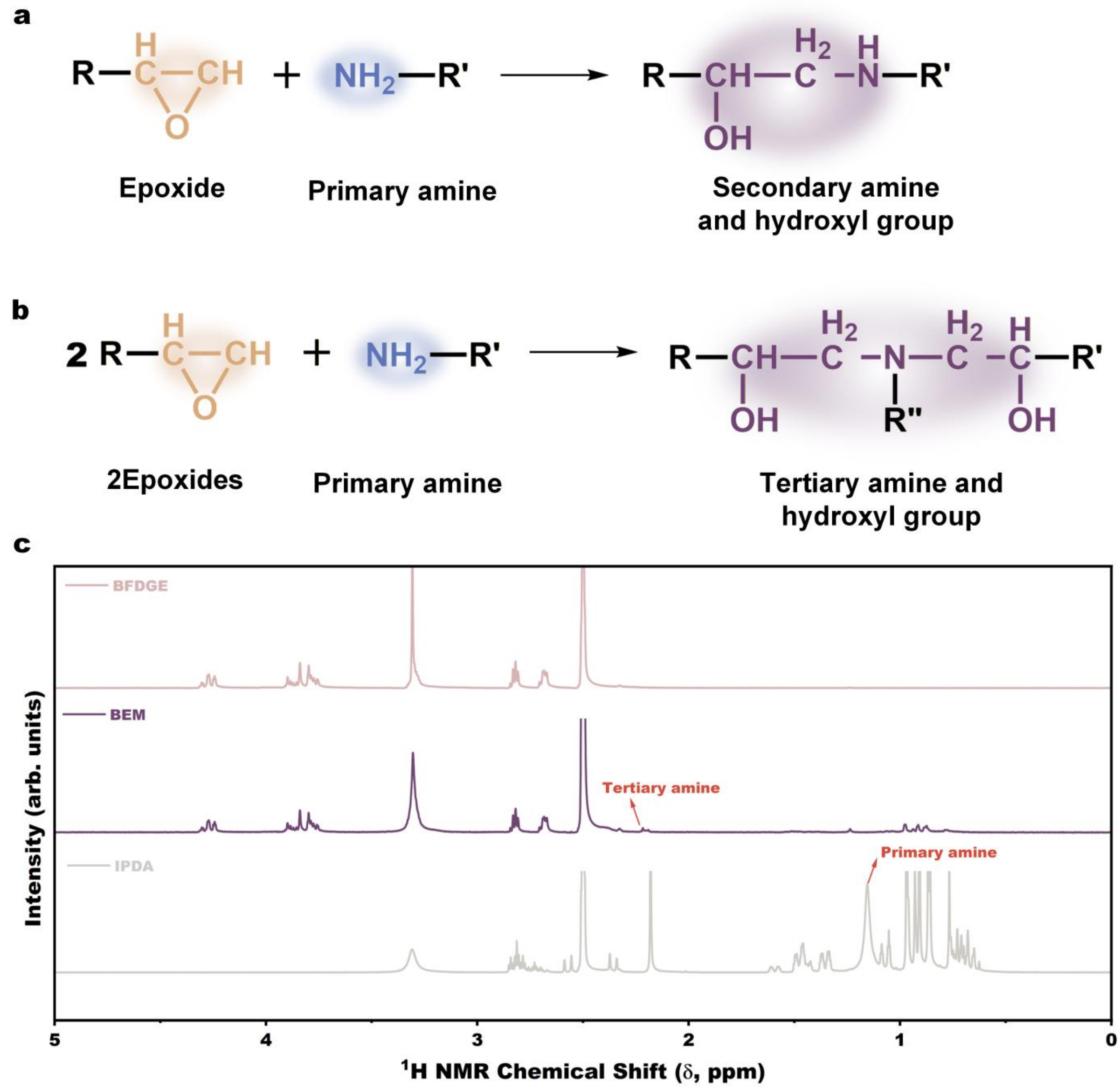

Figure S2. Ring-opening reactions of epoxide within amine and hydroxyl groups forming. (a) Secondary amine, (b) Tertiary amine and (c) ^{1}H NMR spectra for BFDGE, IPDA and BEM dissolved in DMSO-*d6*.

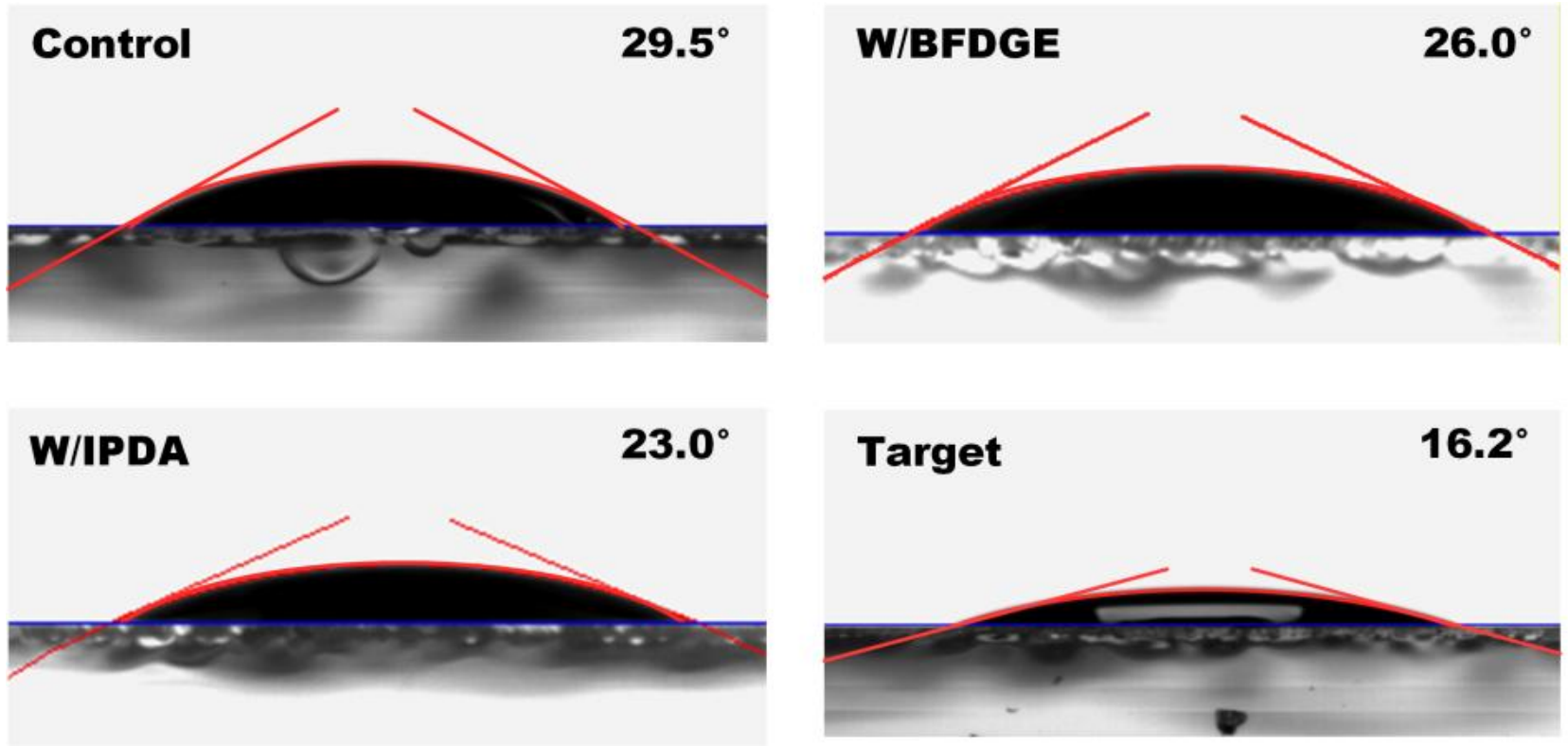

Figure S3. Contact angles of the perovskite precursor on the glass/ITO/SAM substrates.

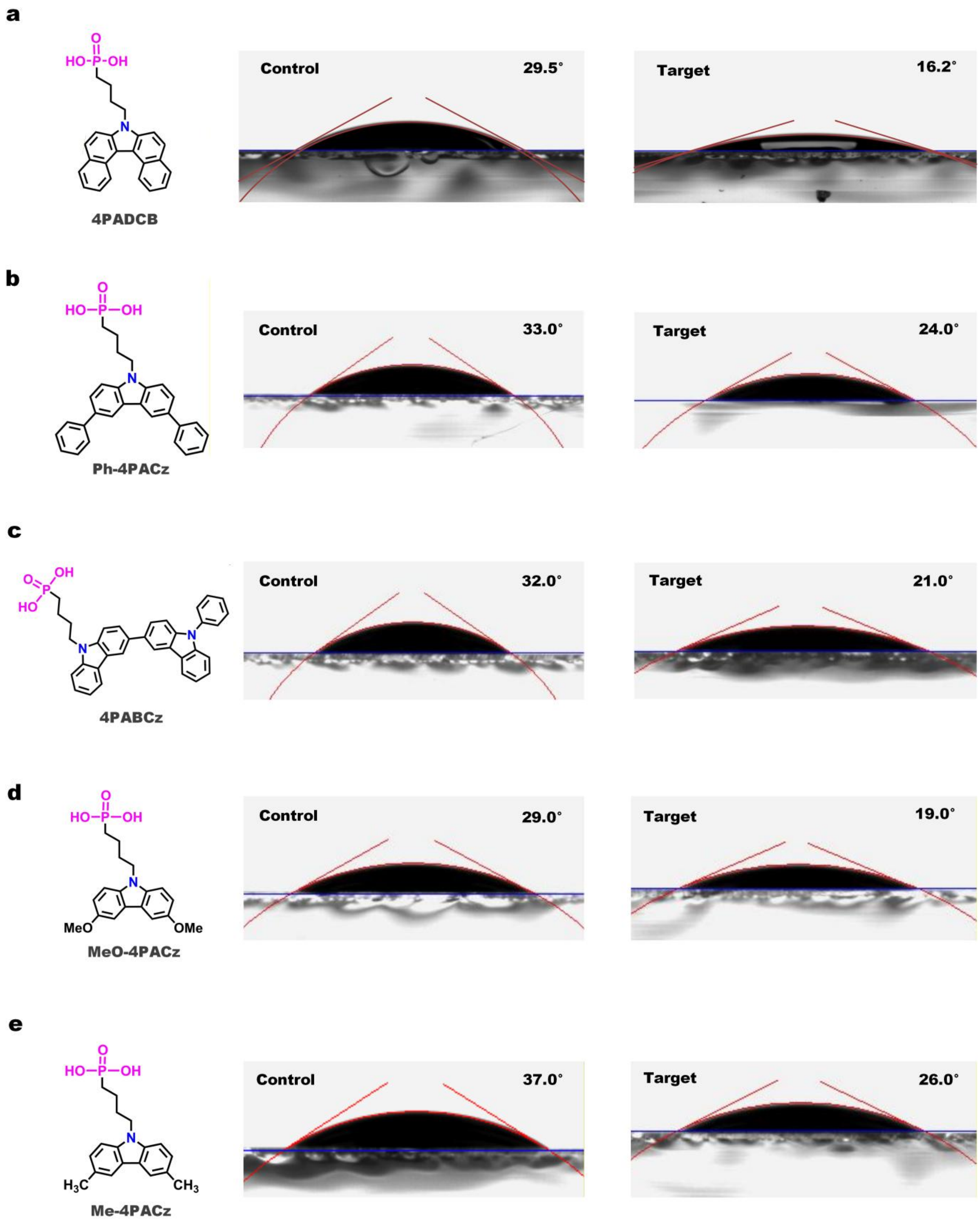

Figure S4. Comparison of contact angles for control and target perovskite inks on diverse SAM-based hole transport layers.

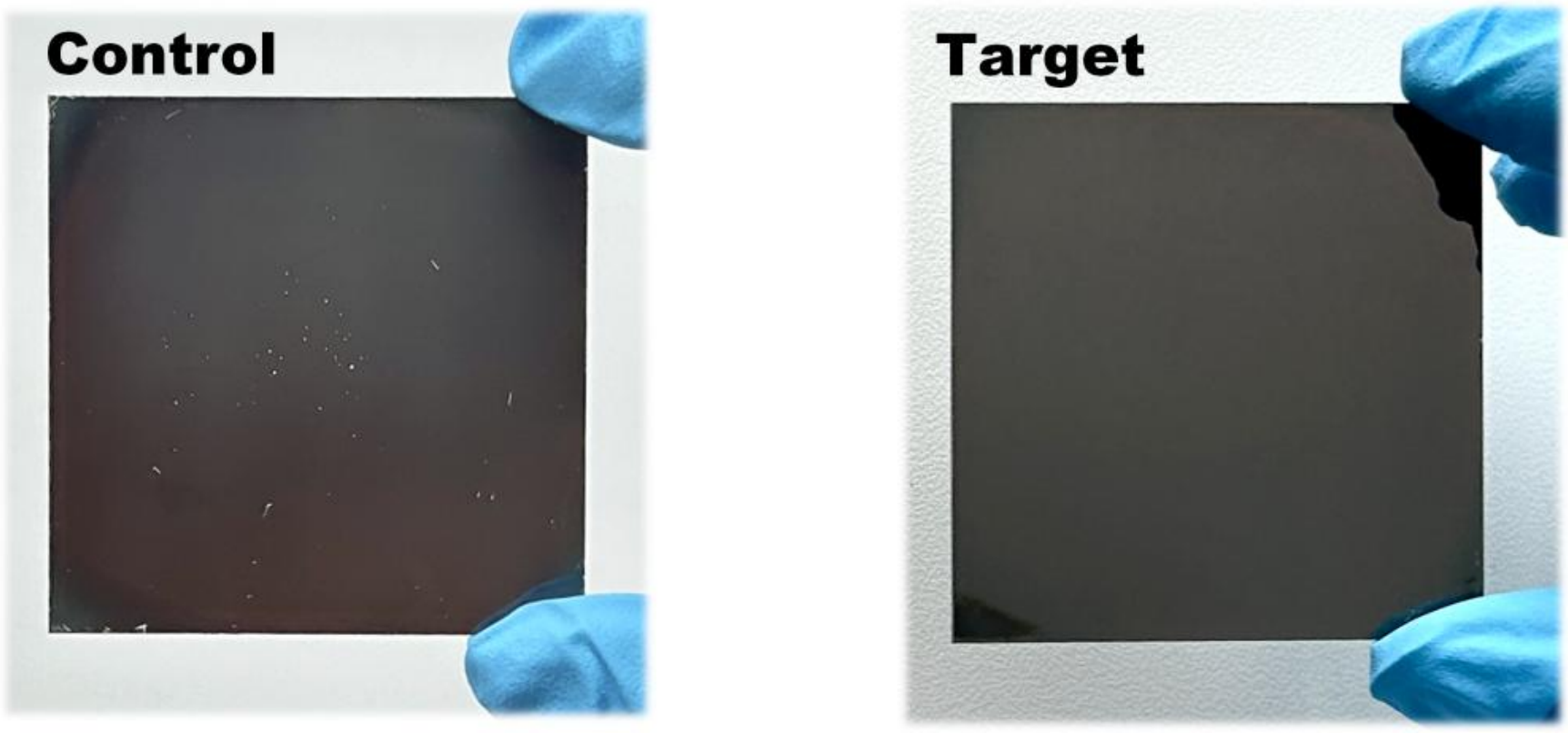

Figure S5. Photographic images of large-area perovskite films deposited on 5×5 cm^2 glass/ITO/SAM substrates.

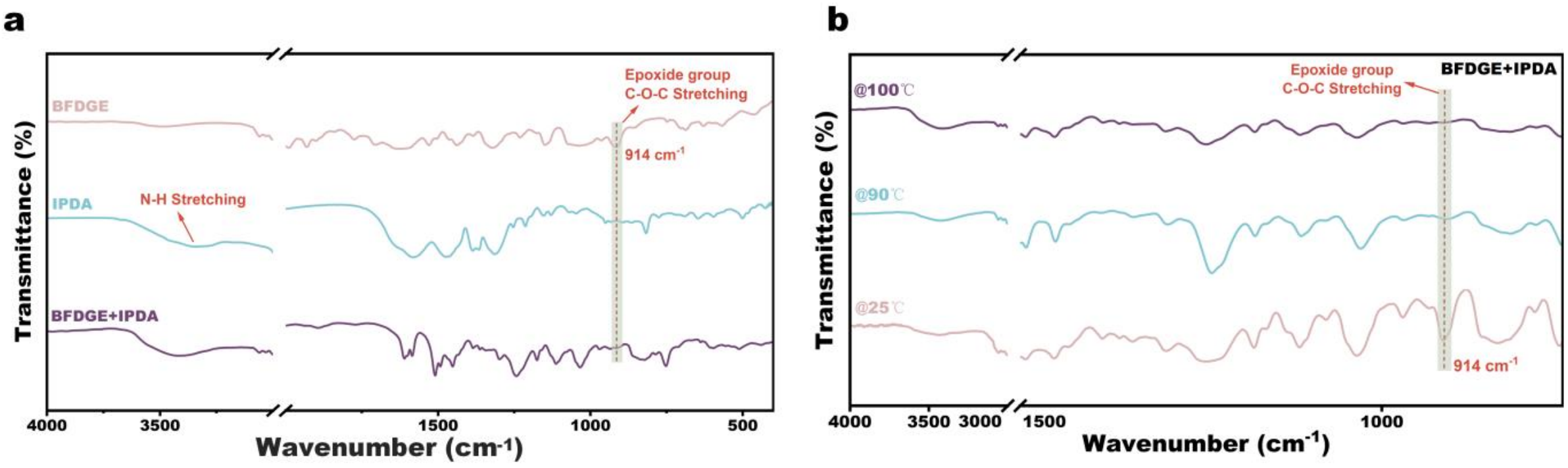

Figure S6. Confirmation of polymerization process. (a) Fourier-transform infrared spectra (FTIR) of the BFDGE monomers, pure IPDA, and BFDGE + IPDA mixture after heating at 100 ℃ for 5 min. (b) varied-temperature FTIR spectra of BFDGE + IPDA mixture.

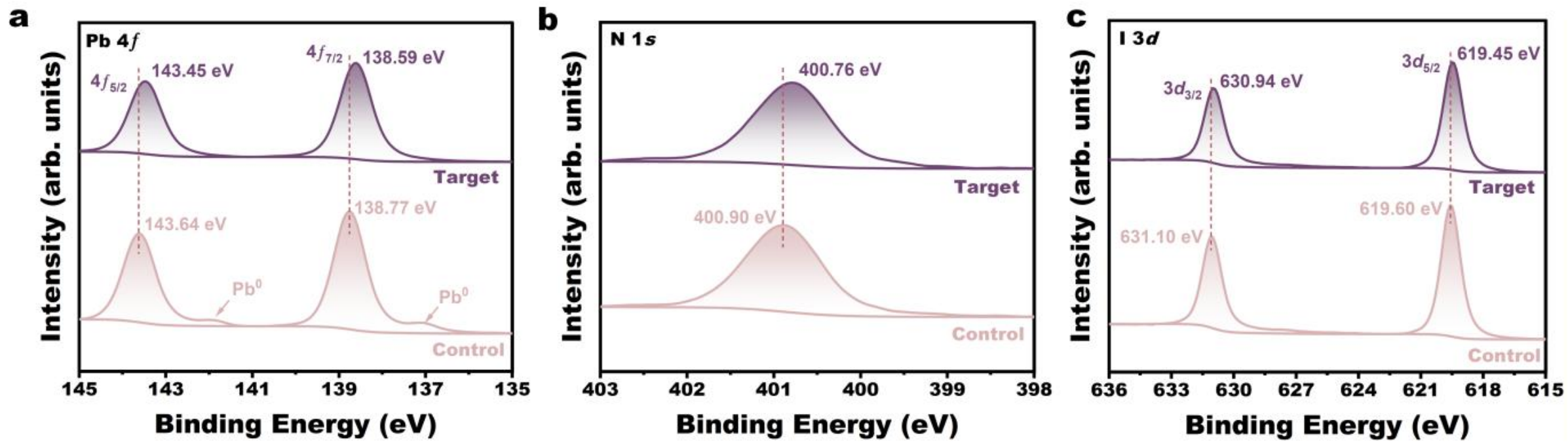

Figure S7. High-resolution XPS spectra of Pb 4f (a), N1s (b), I 3d (c) core levels for control and target perovskite films.

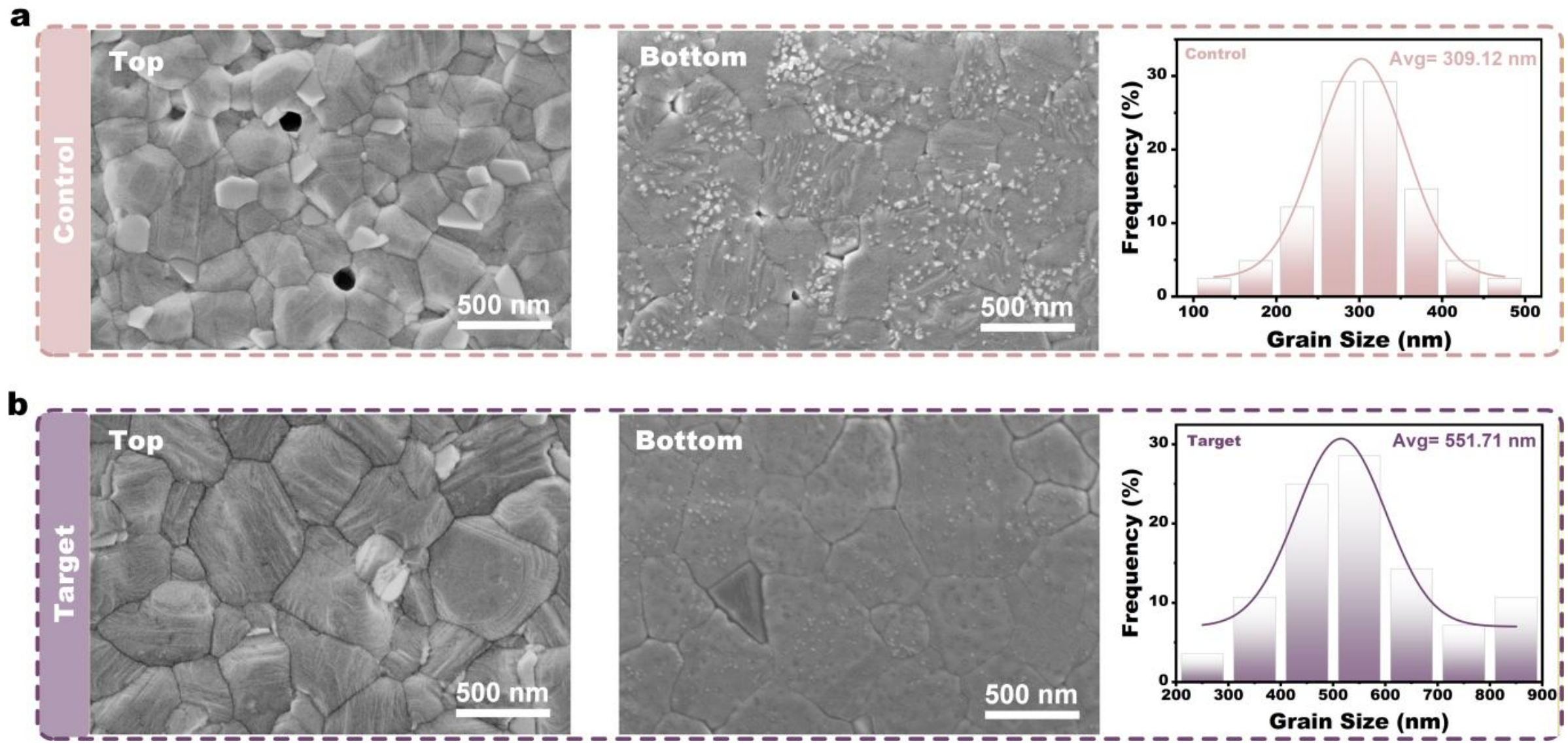

Figure S8. SEM images and the corresponding grain size distribution of the control (a), target (b) perovskite films.

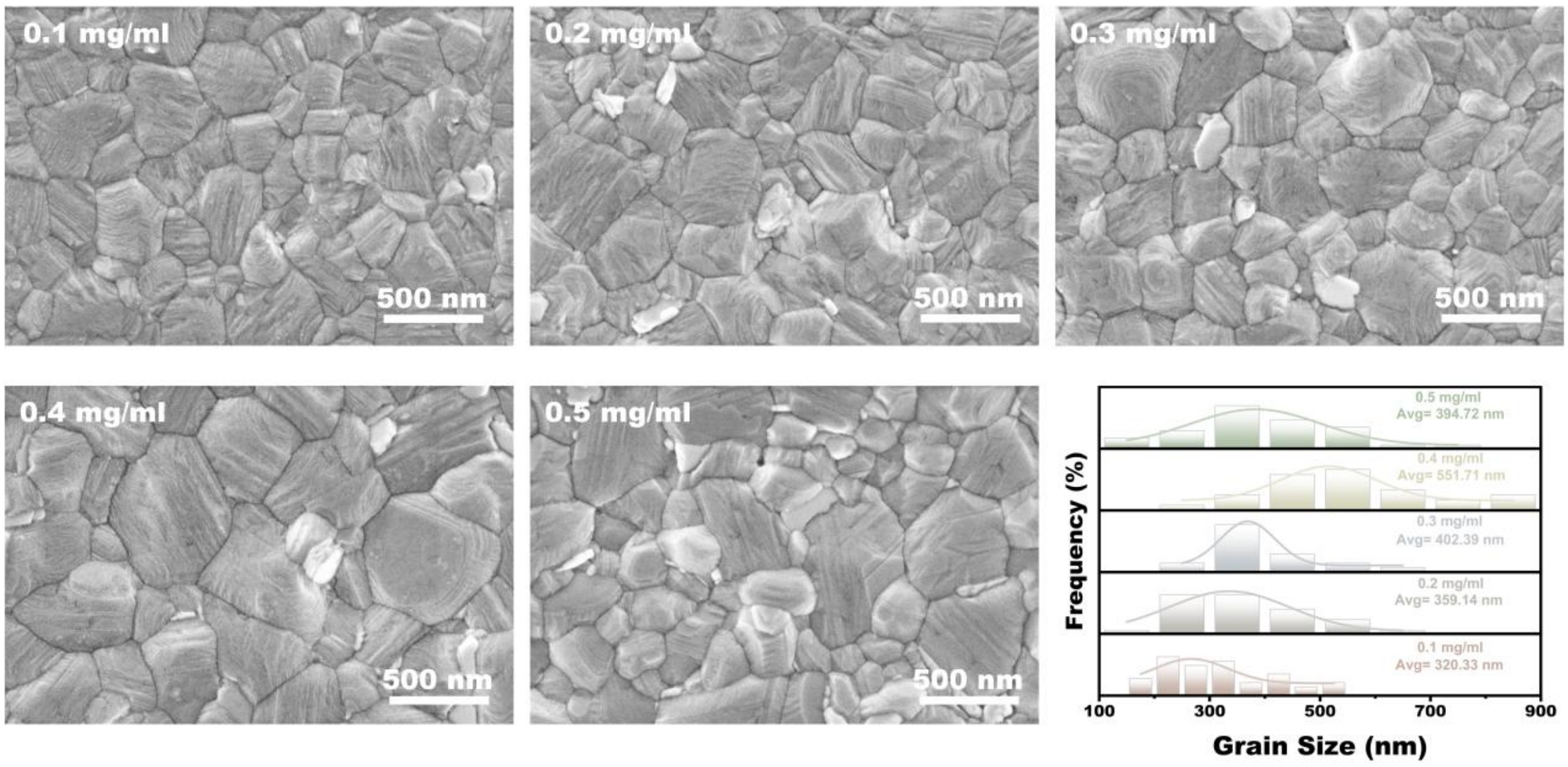

Figure S9. SEM images of perovskite films with different concentrations of BFDGE.

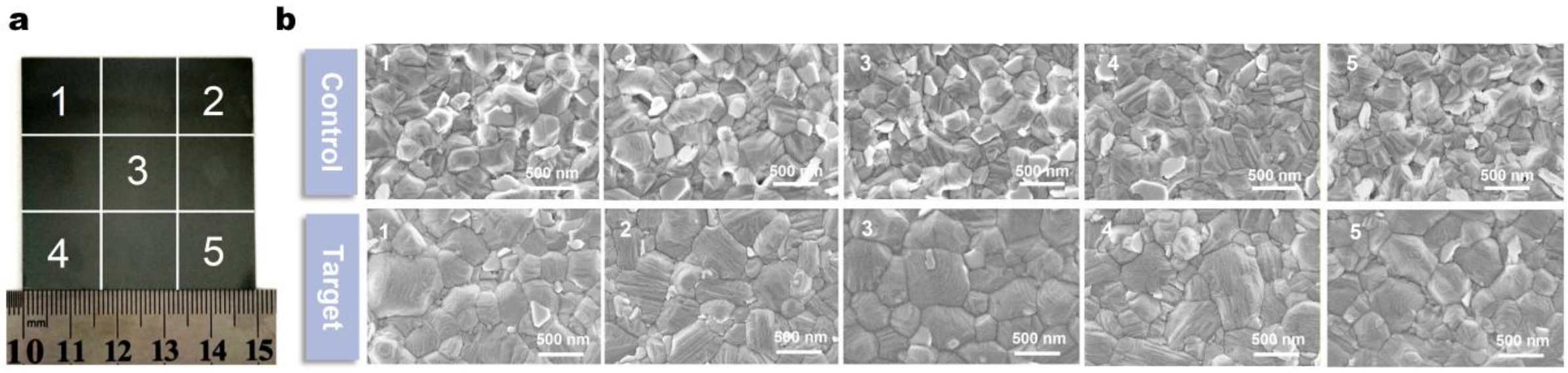

Figure S10. (a) Photograph of perovskite film deposited on 5×5 cm^2 substrate. (b) SEM images of five regions in the large-area perovskite films.

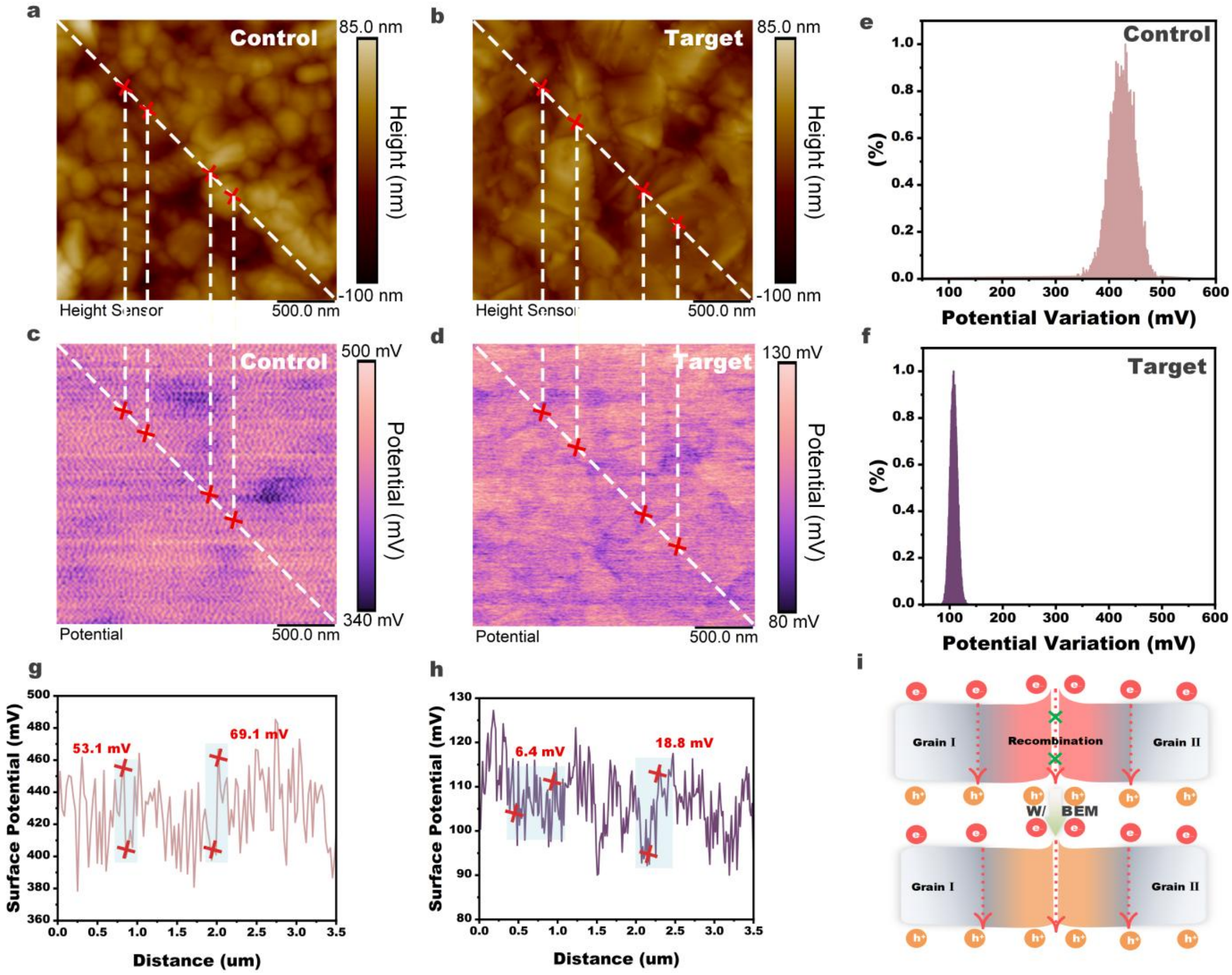

Figure S11. Surface morphology and potential distribution of perovskite films. (a, b) AFM images of control (RMS= 23.6 nm) and target (RMS= 14.3 nm) perovskite films. (c-f) KPFM images and the surface potential variation of perovskite films (g, h) The corresponding surface potential variation between grains and grain boundaries along the diagonal line for control and BEM-modified perovskite films. (i) Schematic diagram of the intergranular carrier transfer process.

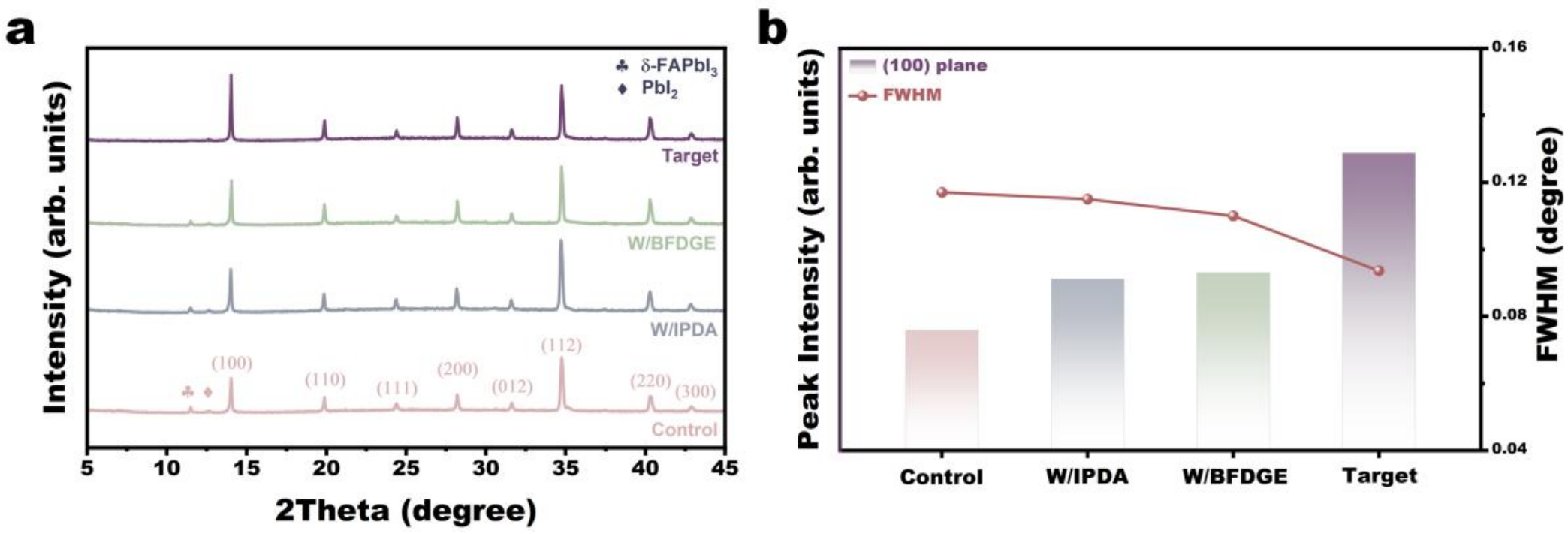

Figure S12. XRD patterns of control, target perovskite films and the corresponding FWHM of (100) facet.

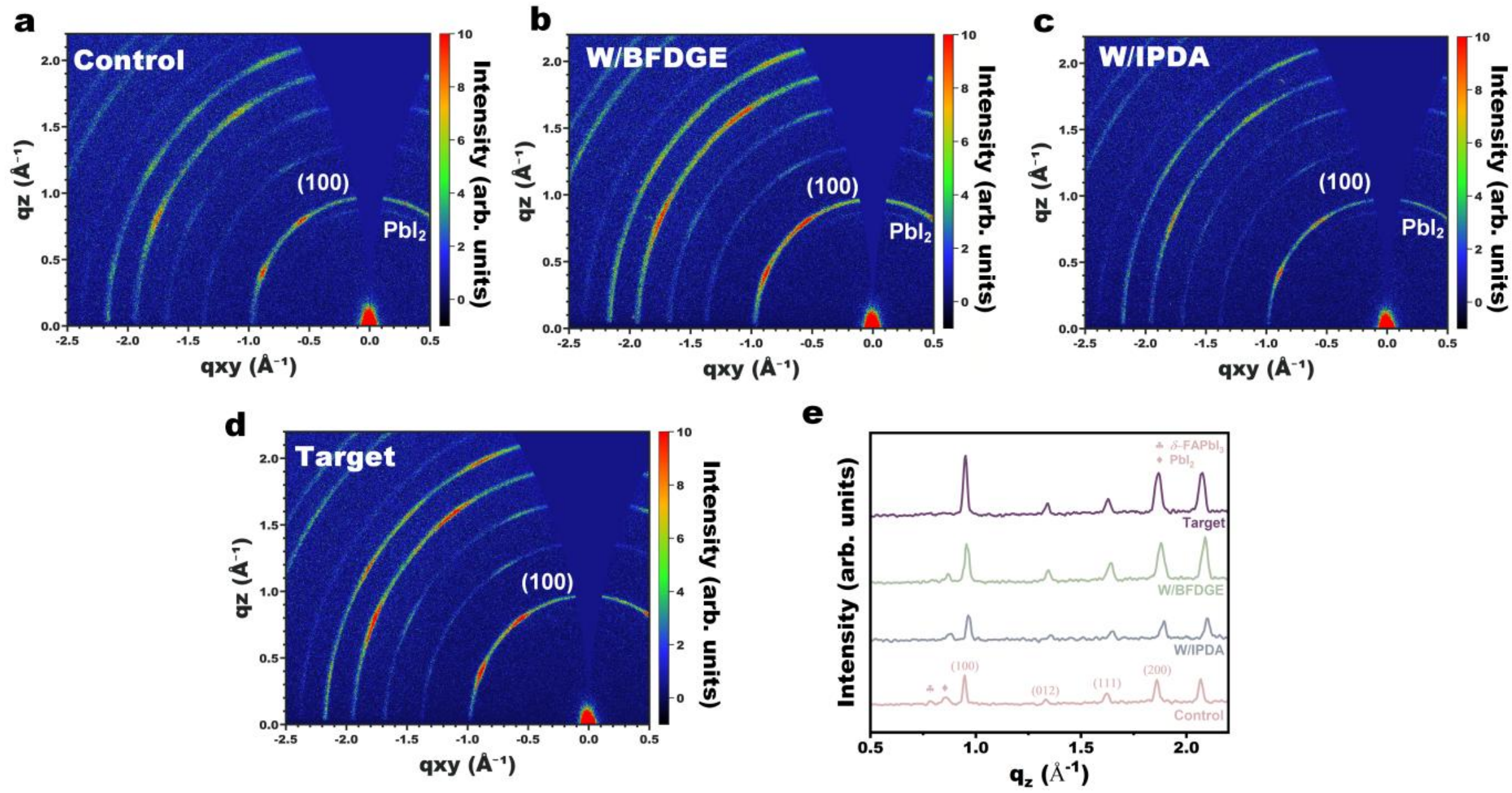

Figure S13. 2D-GIWAXS patterns of (a) Control, (b) pure BFDGE treated-, (c) pure IPDA treated-, and (d) Target perovskite films. (e) The corresponding line cuts of GIWAXS patterns.

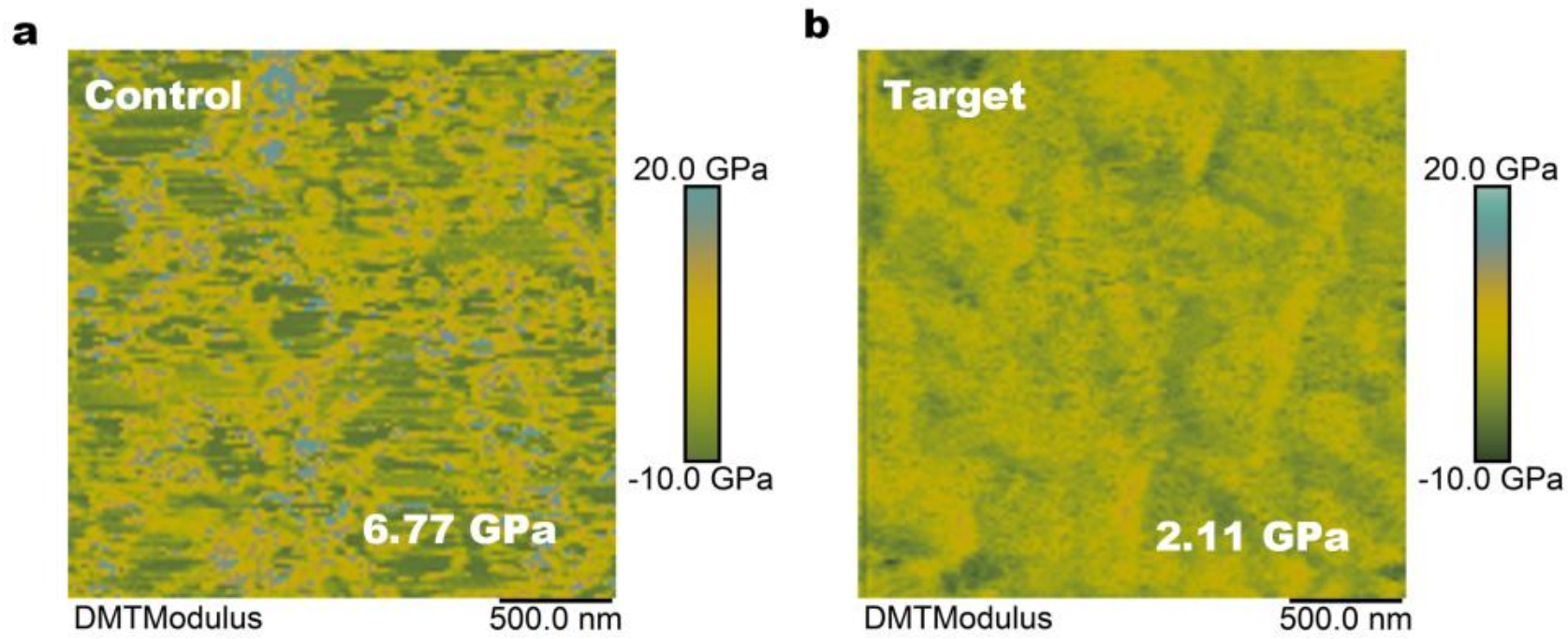

Figure S14. Young's modules of perovskite films.

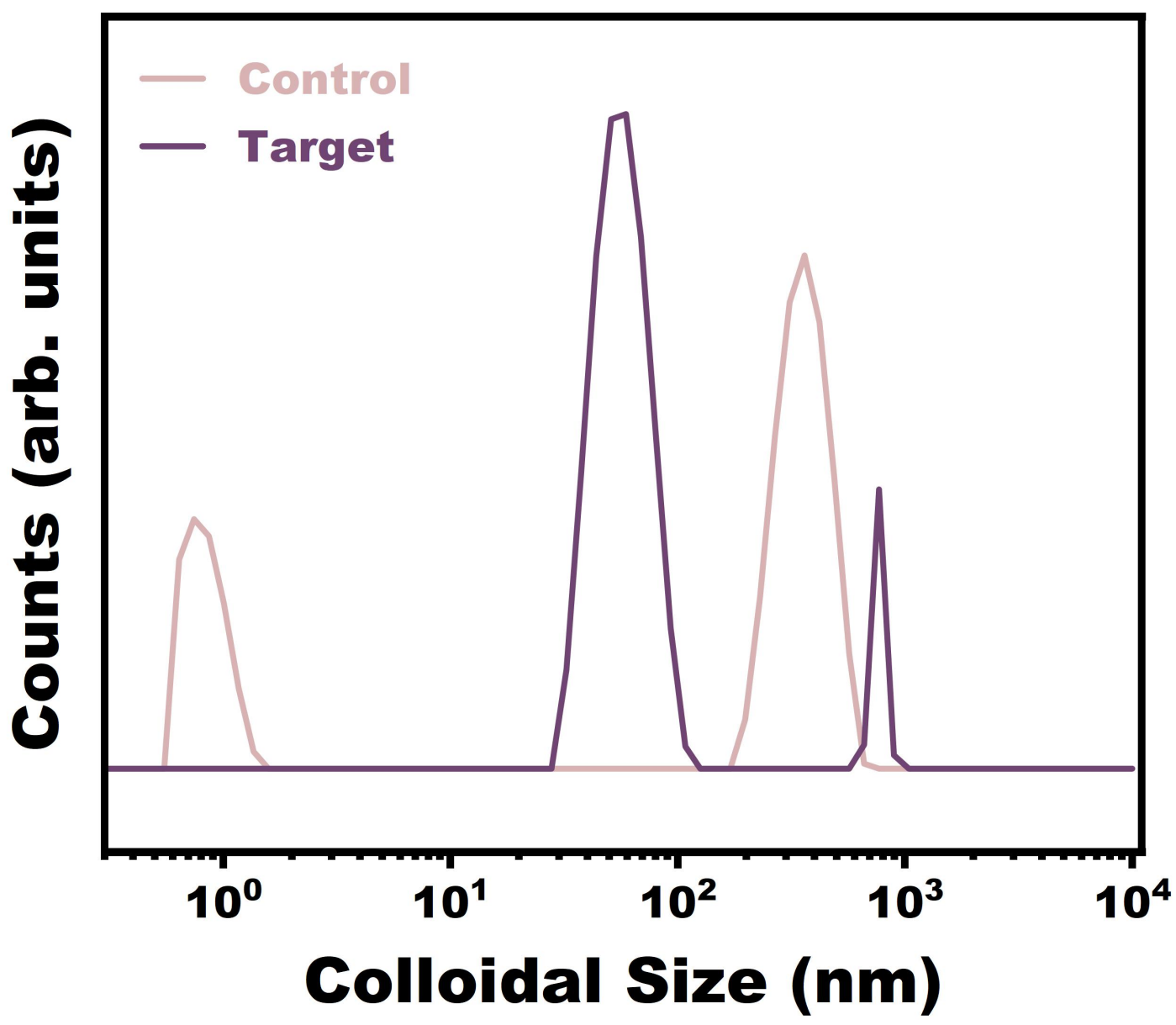

Figure S15. Dynamic Light Scattering (DLS) spectra of the control and target perovskite precursor.

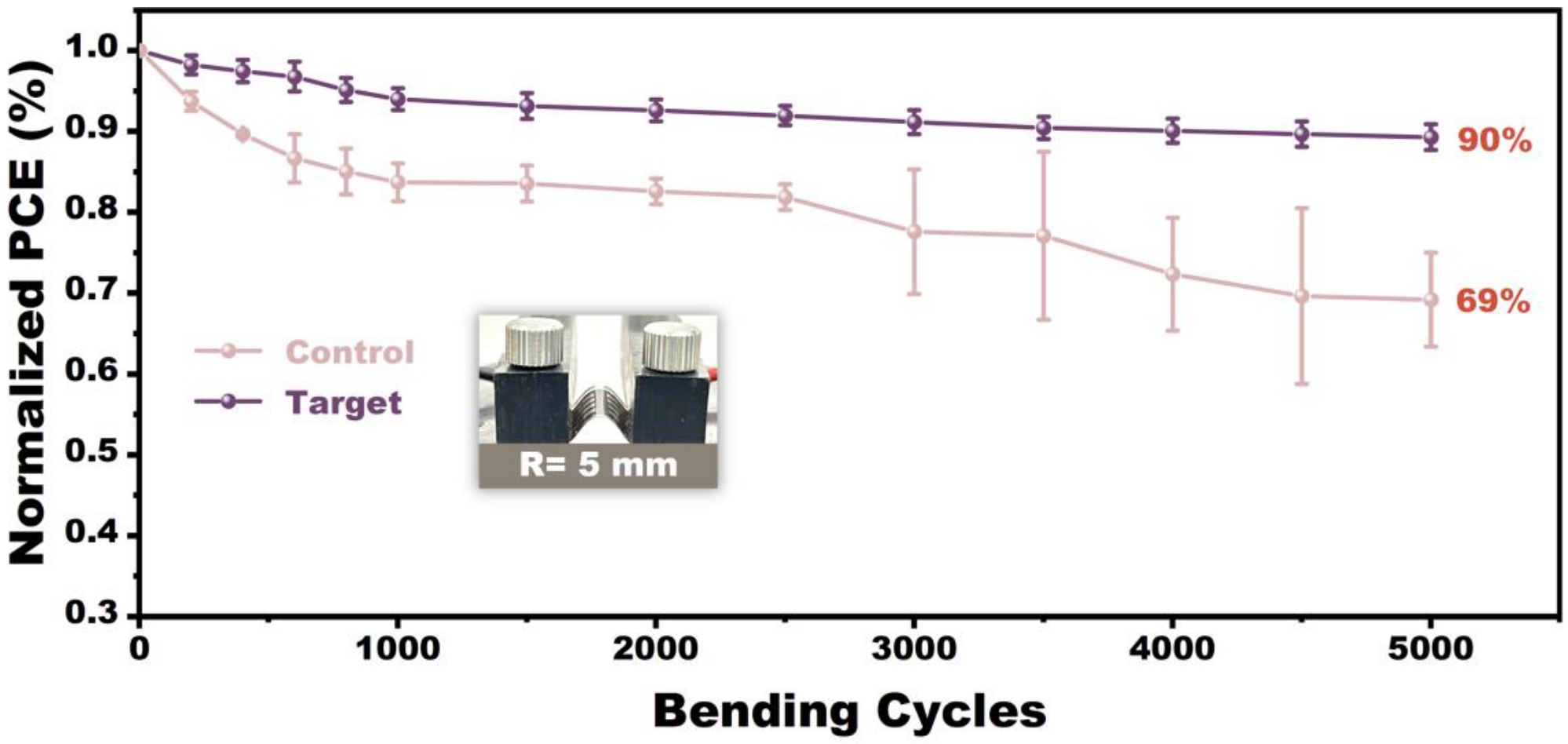

Figure S16. Mechanical stability of the f-PSCs versus bending cycles at a radius of 5 mm.

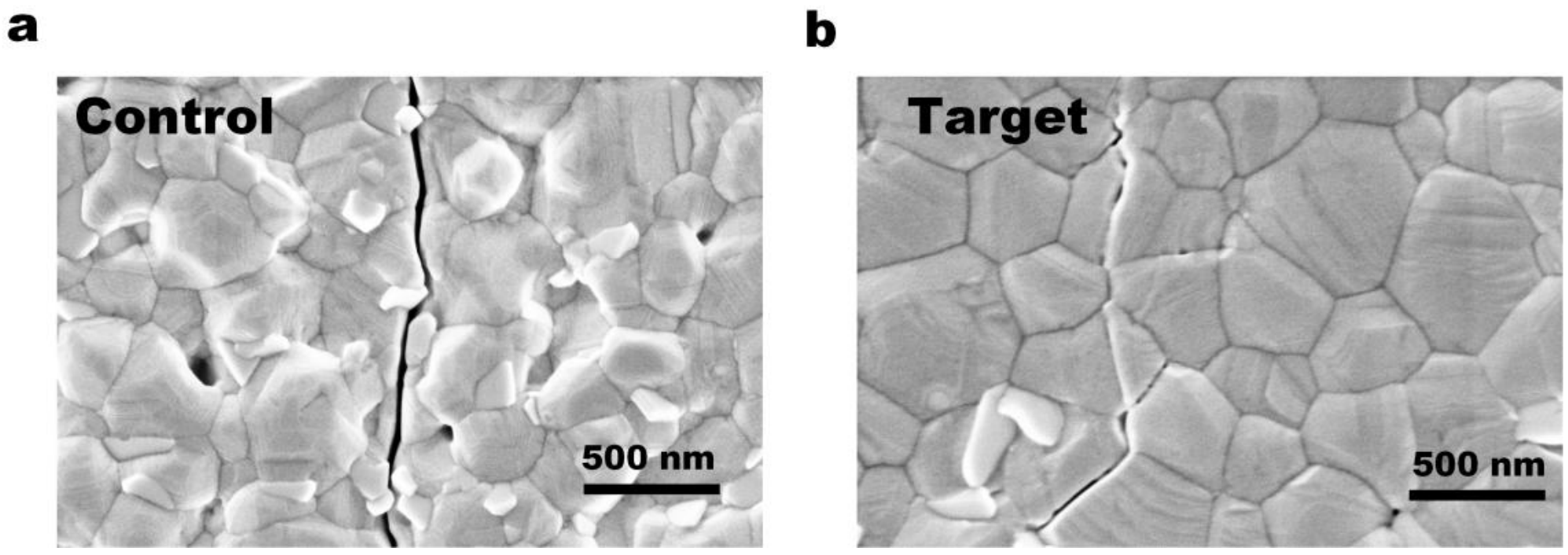

Figure S17. Top-view SEM images of the control and target perovskite films after 5000 bending cycles.

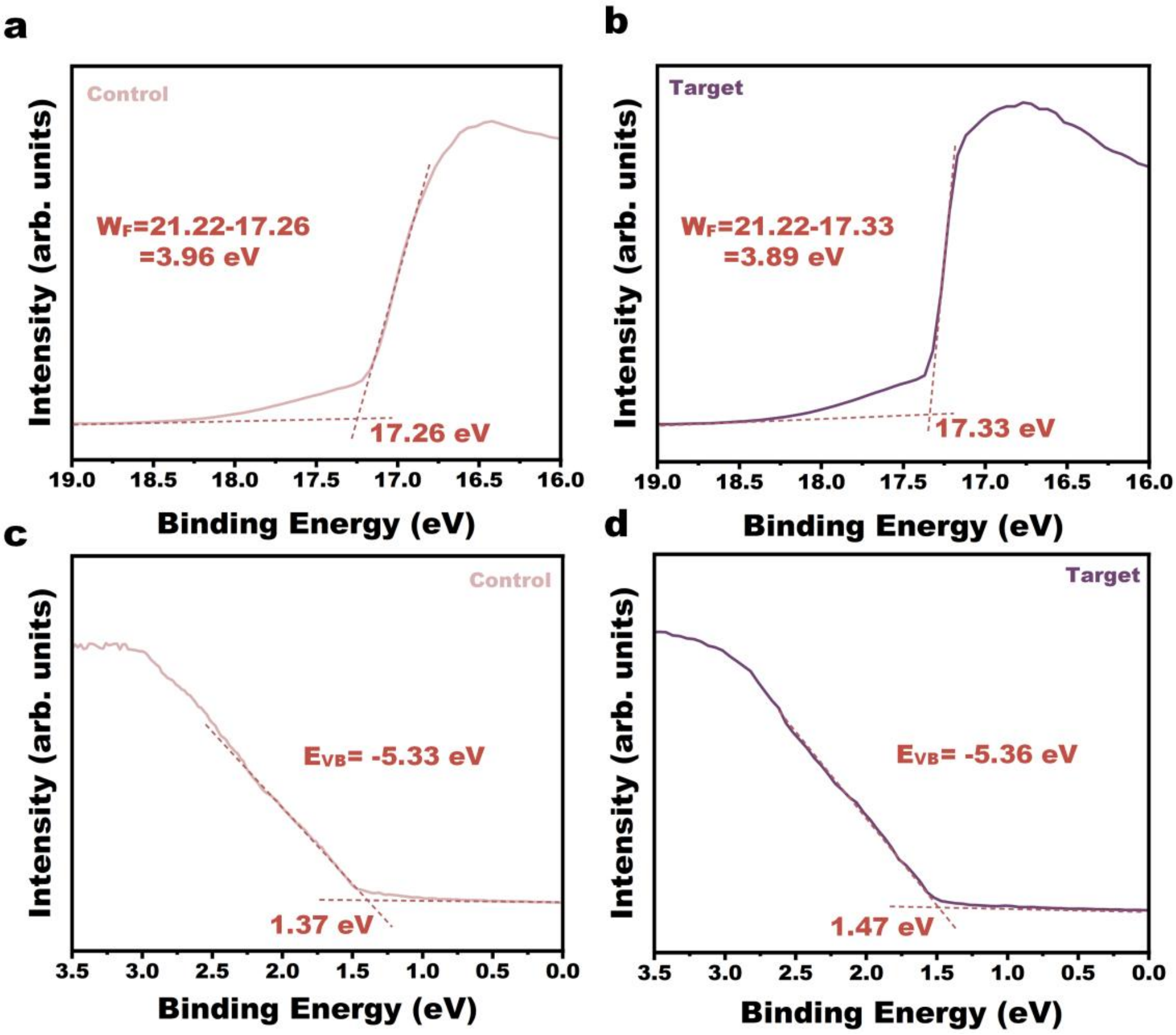

Figure S18. UPS spectra of perovskite films. (a, b) in the secondary-cutoff region. (c, d) in the valance band region.

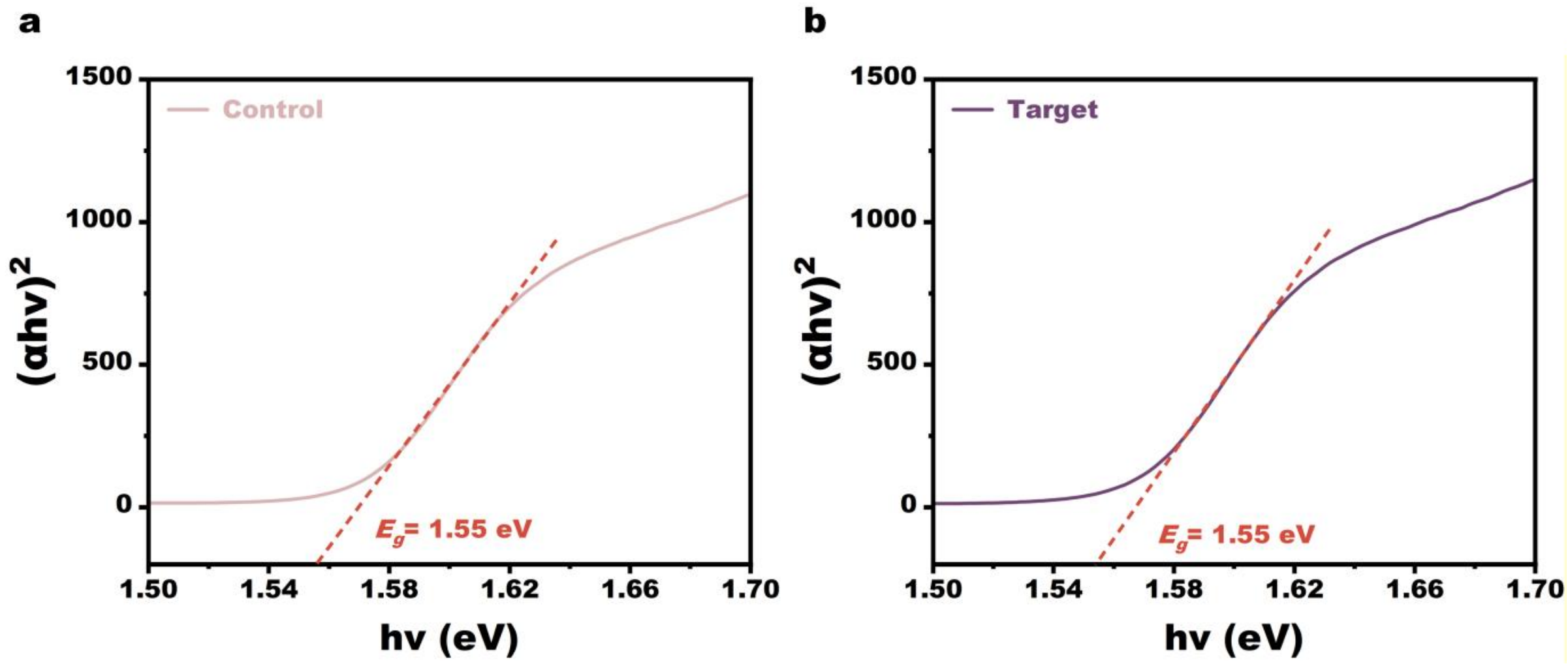

Figure S19. The UV-vis absorbance spectrum of the perovskite films.

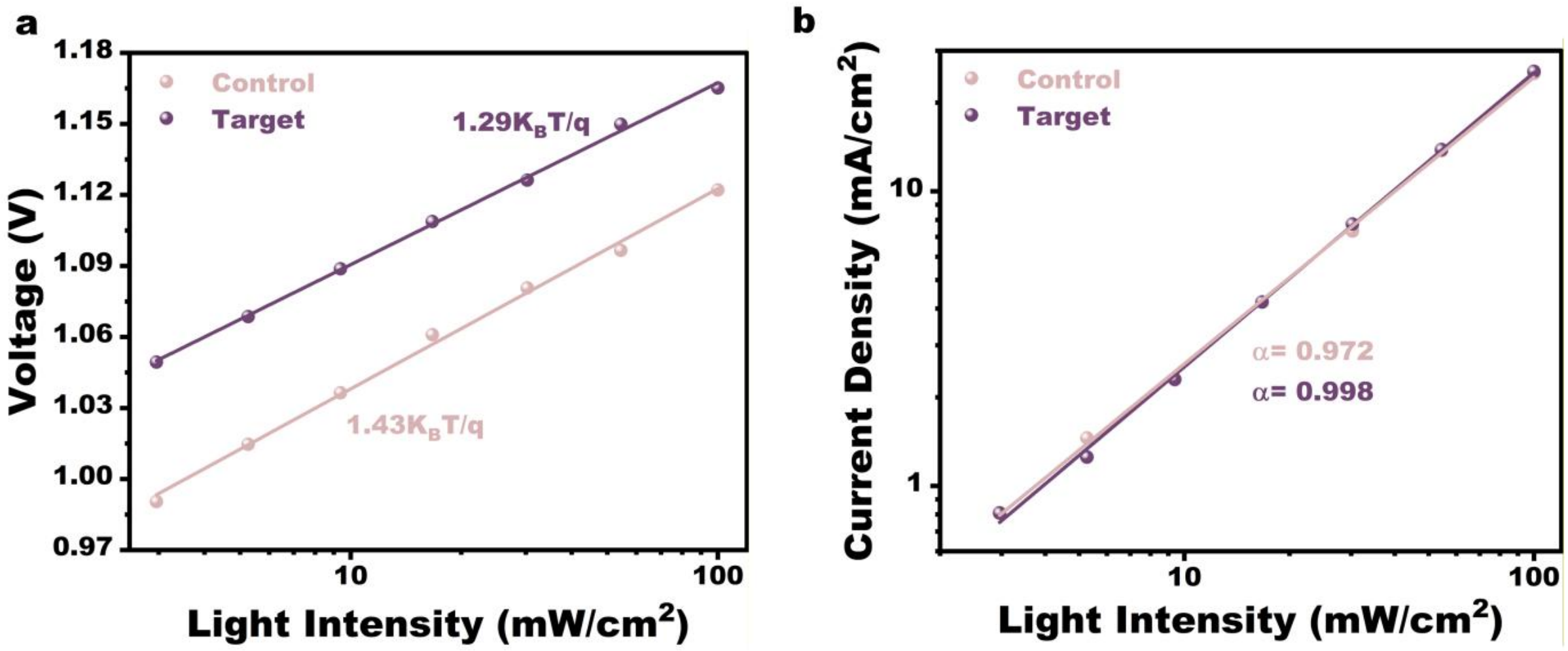

Figure S20. The light intensity dependence of the devices. (a) V_{OC} versus light intensity of the devices. (b) J_{SC} versus light intensity of the devices.

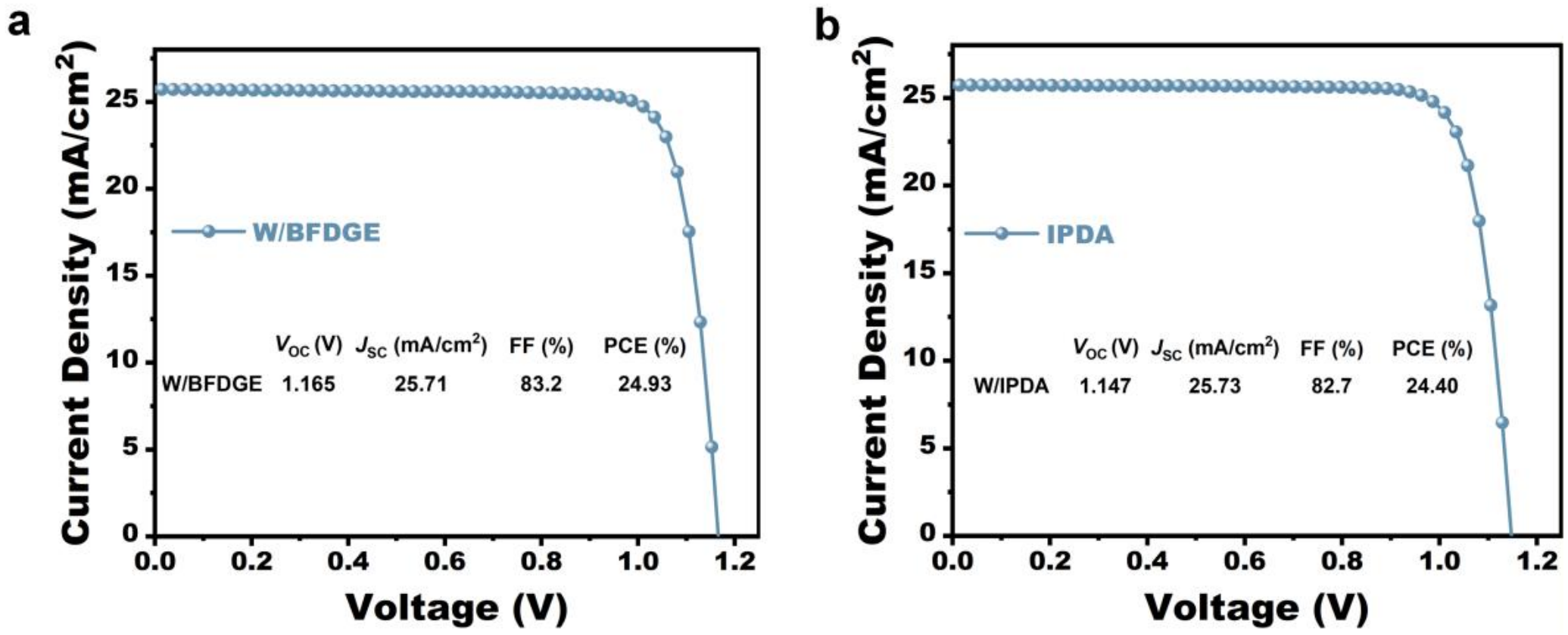

Figure S21. *J-V* curves of devices modified solely with BFDGE and solely with IPDA.

Report No. 25TR112502

Sample Information

Sample Type	p-i-n perovskite solar cell
Serial No.	6-8
Lab Internal No.	25112501-2#
Measurement Item	I-V characteristic
Measurement Environment	(23.5±2.0) °C, (35.5±5.0) %R.H

Measurement of I-V characteristic

**Reference cell**	PVM1121, mono-Si, WPVS, calibrated by NIM (Certificate No. GXgf2025-01762)
**Calibration Value/Date of Calibration for Reference cell**	143.3 mA/ May. 2025
**Measurement Conditions**	Standard Test Condition (STC): AM1.5, 1000W/m², 25°C
**Measurement Equipment/ Date of Calibration**	AAA Steady State Solar Simulator (YSS-T155-2M) / Oct.2025<br>IV test system (ADCMT 6246) / June. 2025<br>Measuring Microscope (MF-B2017C) / July.2025<br>SR Measurement System (CEP-25ML-CAS) / May.2025
**Measurement Method**	A bidirectional logarithmic voltage sweep, from open circuit voltage (Voc) to short circuit current (Isc) and back, was performed within a single flash in accordance with IEC 60904-1:2020.<br>Spectral Mismatch factor was calculated and the I-V correction was performed according to IEC 60891:2021.
**Measurement Uncertainty**	Area: 1.0%(k=2); Isc: 2.0%(k=2); Voc: 1.0%(k=2);<br>Pmax: 2.4%(k=2); Eff: 2.5%(k=2)

2 / 4

Report No. 25TR112502

====Measurement Results ====

	Forward Scan (Isc to Voc)	Reverse Scan (Voc to Isc)
Area	5.43 mm²	
Isc	1.439 mA	1.439 mA
Voc	1.177 V	1.175 V
Pmax	1.423 mW	1.459 mW
Ipm	1.375 mA	1.388 mA
Vpm	1.035 V	1.051 V
FF	84.00 %	86.28 %
Eff	26.21 %	26.87 %

- Spectral Mismatch Factor SMM=1.0040.
- Designated illumination area defined by a thin mask was measured by measuring microscope.
- Test results listed in this measurement report refer exclusively to the mentioned measured sample.
- The results apply only at the time of the test, and do not imply future performance.

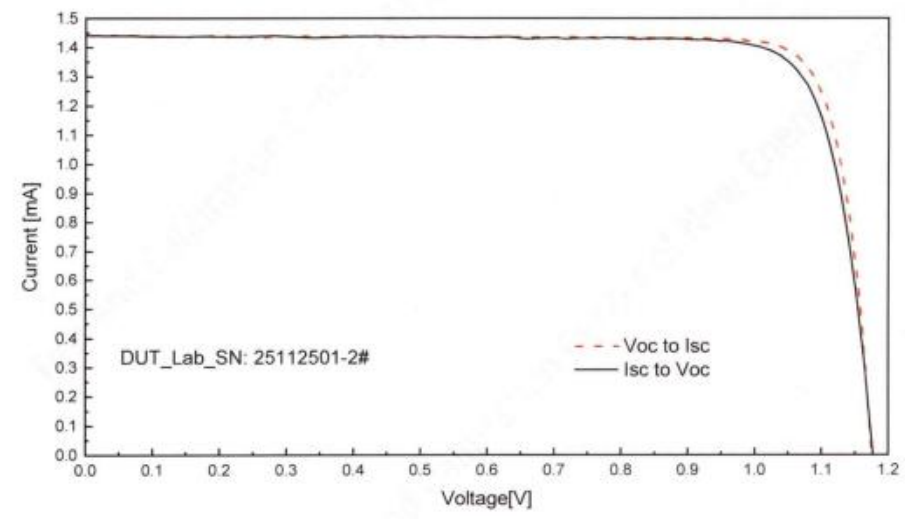

Fig.1 I-V curves of the measured sample

3 / 4

Figure S22. Certification report of the small-area target device.

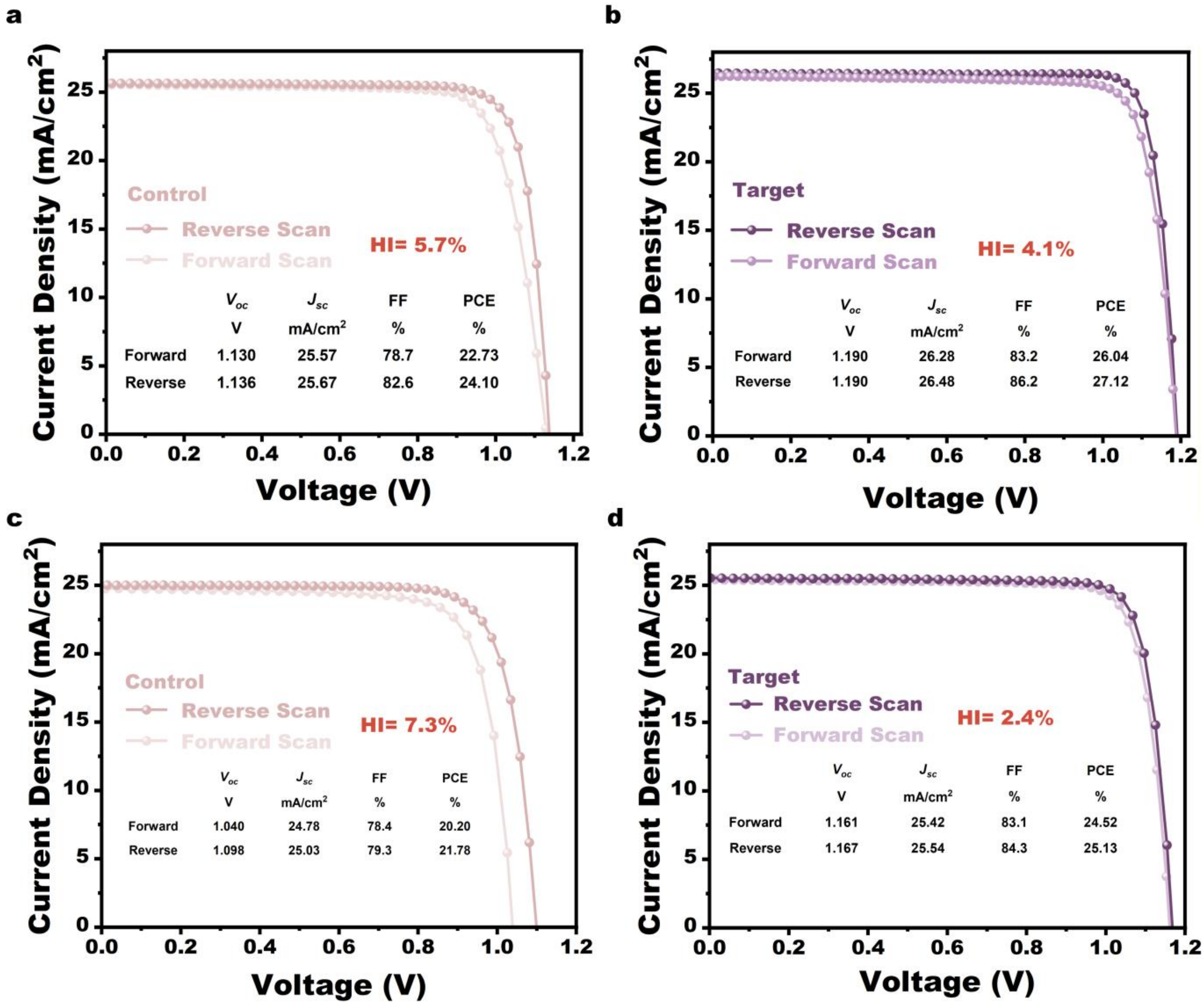

Figure S23. ***J-V* curves with forward and reverse scan for devices.** (a, b) Rigid PSCs. (c, d) Flexible PSCs.

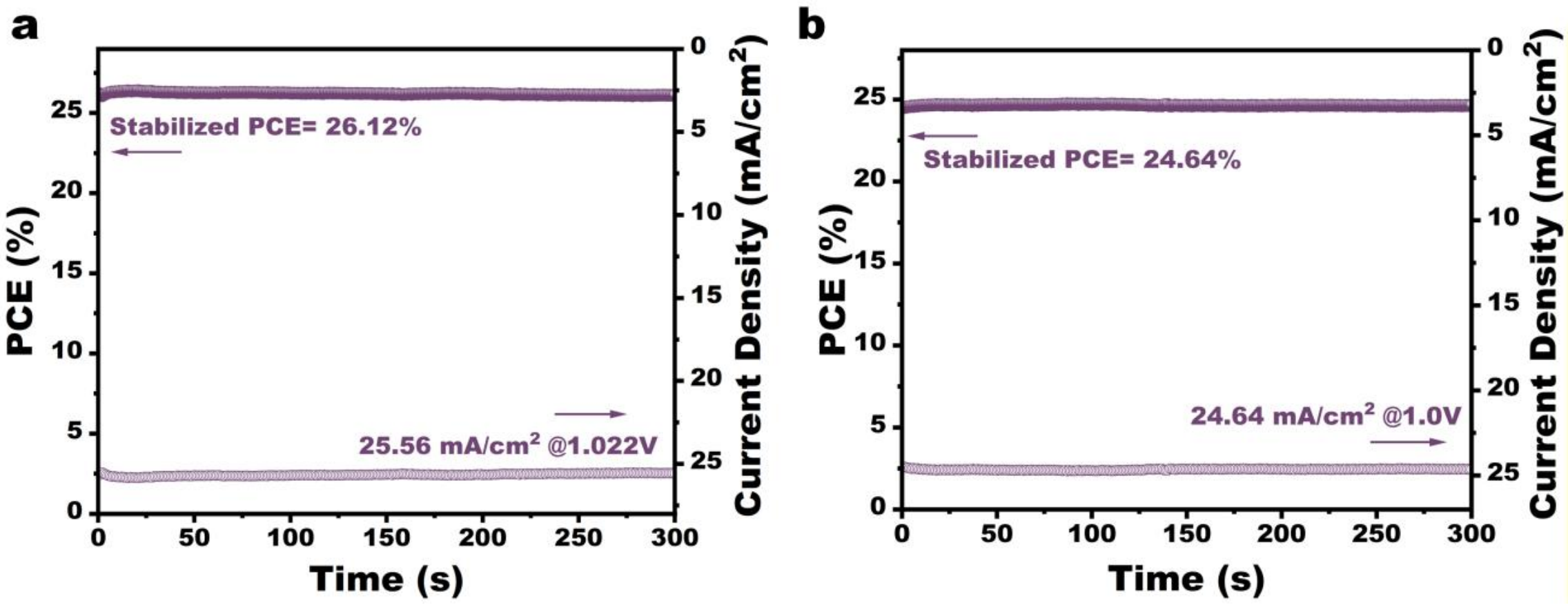

Figure S24. The stable power output and steady-state current density of the best-performance target devices, (a) rigid device, (b) flexible device.

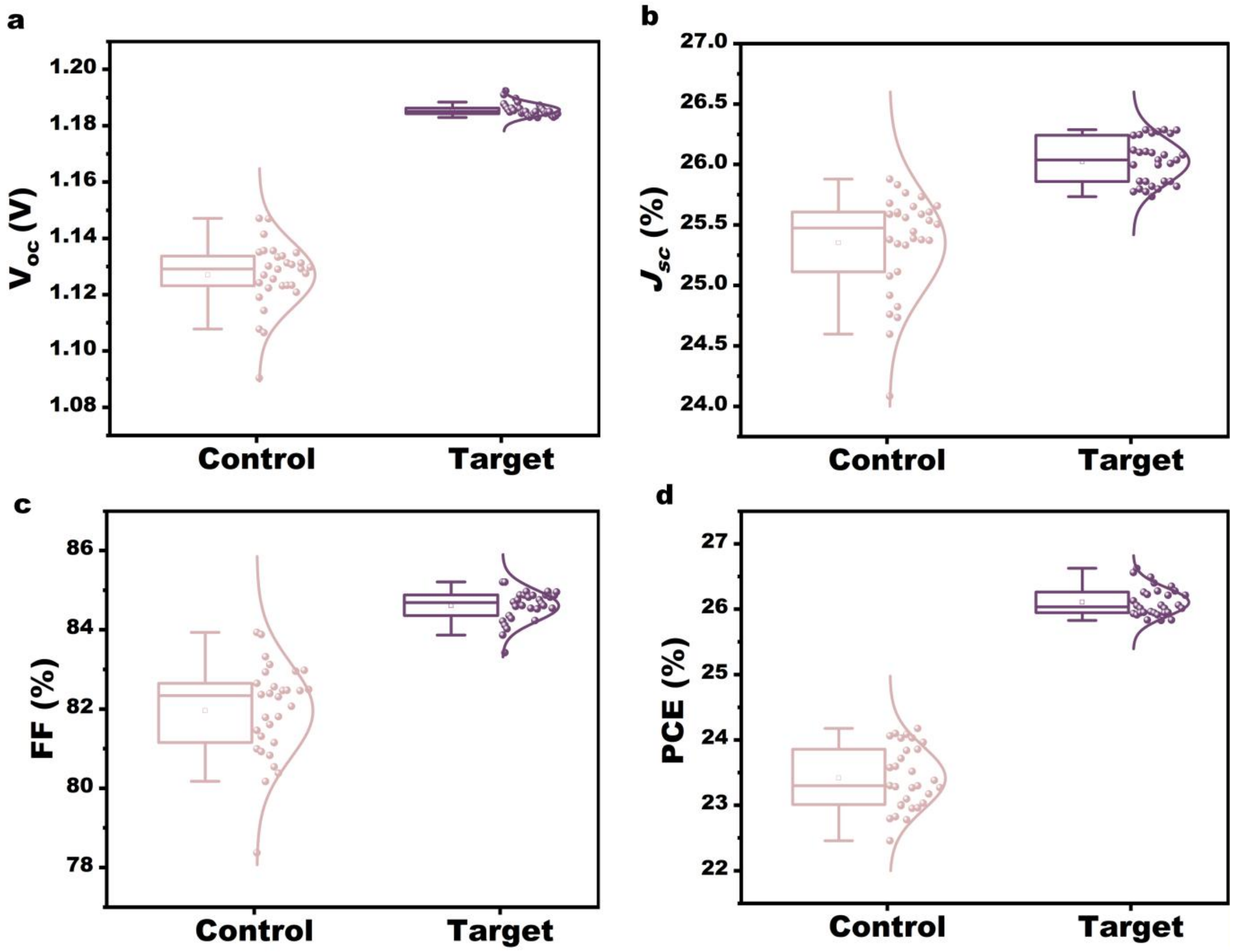

Figure S25. Statistical distribution of photovoltaic parameters from 30 individual rigid devices. (a) V_{OC} normal distribution. (b) J_{SC} normal distribution. (c) FF normal distribution. (d) PCE normal distribution. Each dataset represents 30 independent devices. The box-and-whisker plot shows the distribution of data. The minimum value is indicated by the lower whisker, whereas the maximum value is shown by the upper whisker. The box represents the interquartile range, with the first quartile at the lower edge, the median as the line inside the box and the third quartile at the upper edge. The central line of the box plot represents the media, and the open square represents the mean value. Outliers are represented as individual points beyond the whiskers.

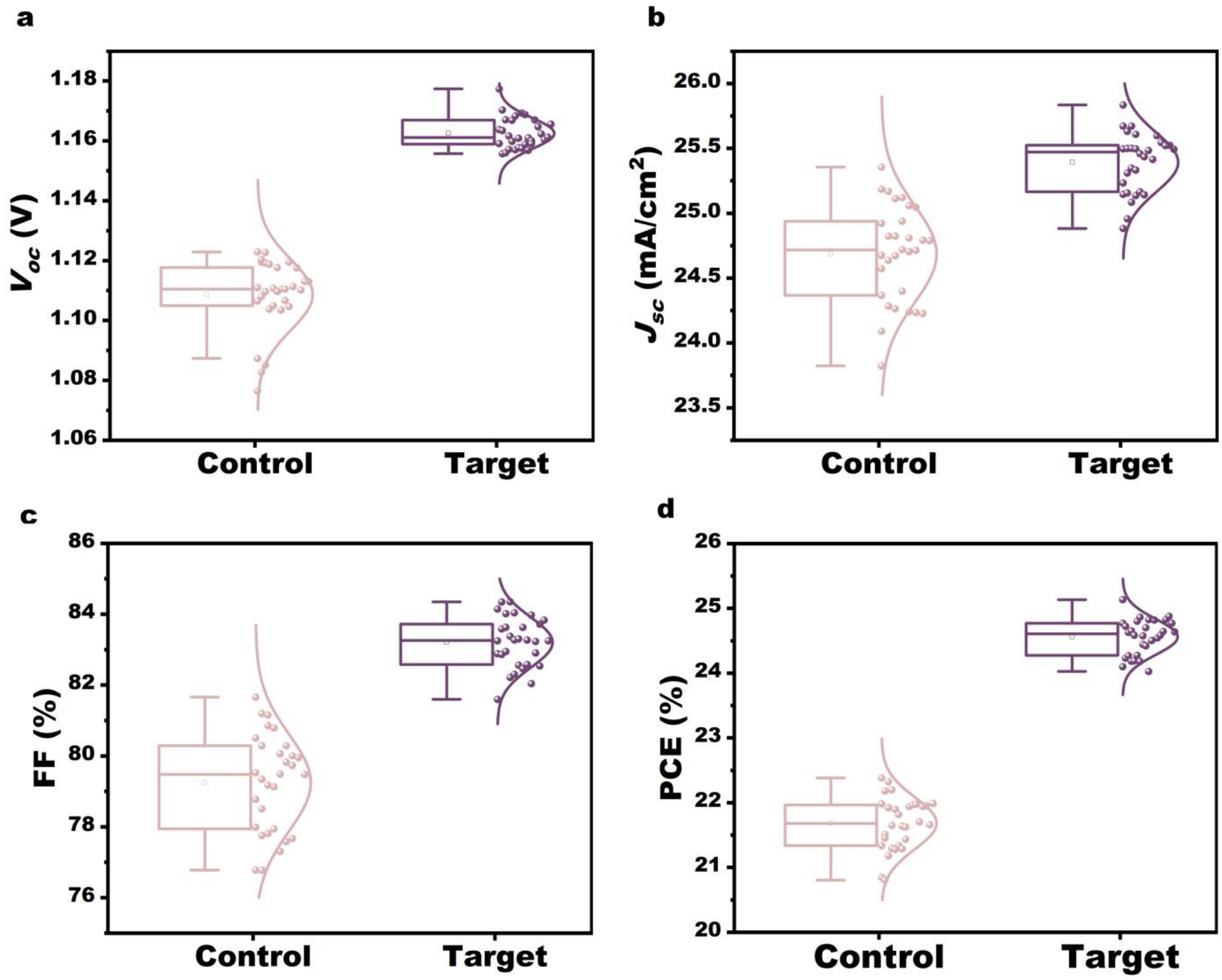

Figure S26. Statistical distribution of photovoltaic parameters from 30 individual flexible devices. (a) V_{OC} normal distribution. (b) J_{SC} normal distribution. (c) FF normal distribution. (d) PCE normal distribution. Each dataset represents 30 independent devices. The box-and-whisker plot shows the distribution of data. The minimum value is indicated by the lower whisker, whereas the maximum value is shown by the upper whisker. The box represents the interquartile range, with the first quartile at the lower edge, the median as the line inside the box and the third quartile at the upper edge. The central line of the box plot represents the media, and the open square represents the mean value. Outliers are represented as individual points beyond the whiskers.

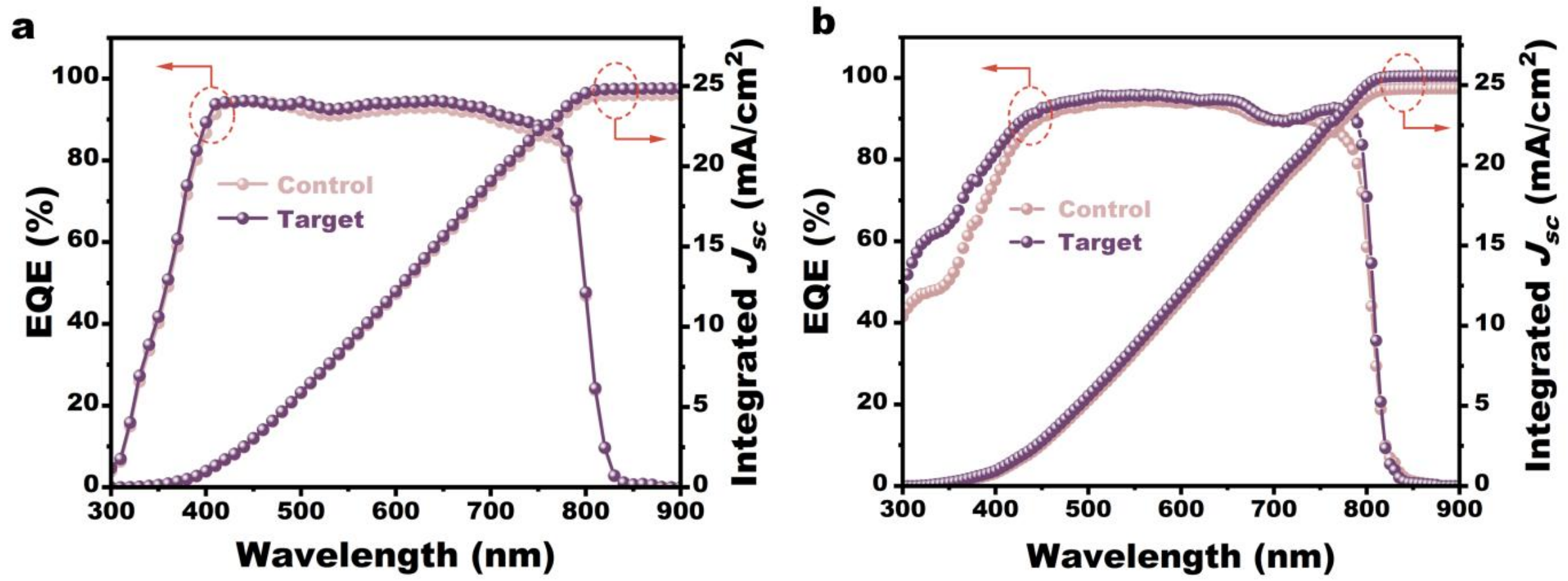

Figure S27. EQE spectra and the corresponding integrated current density of flexible (a) and rigid PSCs (b).

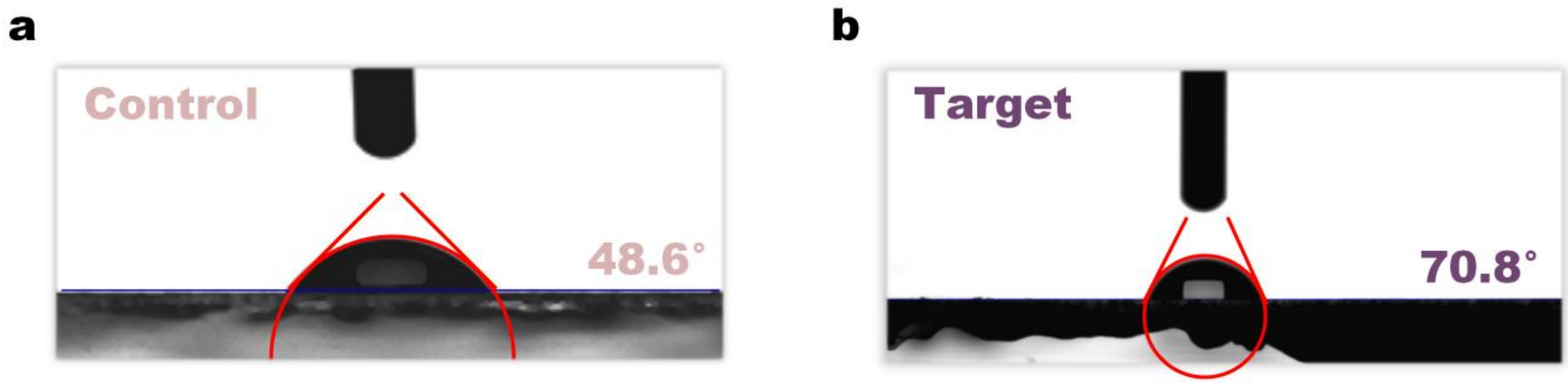

Figure S28. Water contact angles of the corresponding perovskite films.

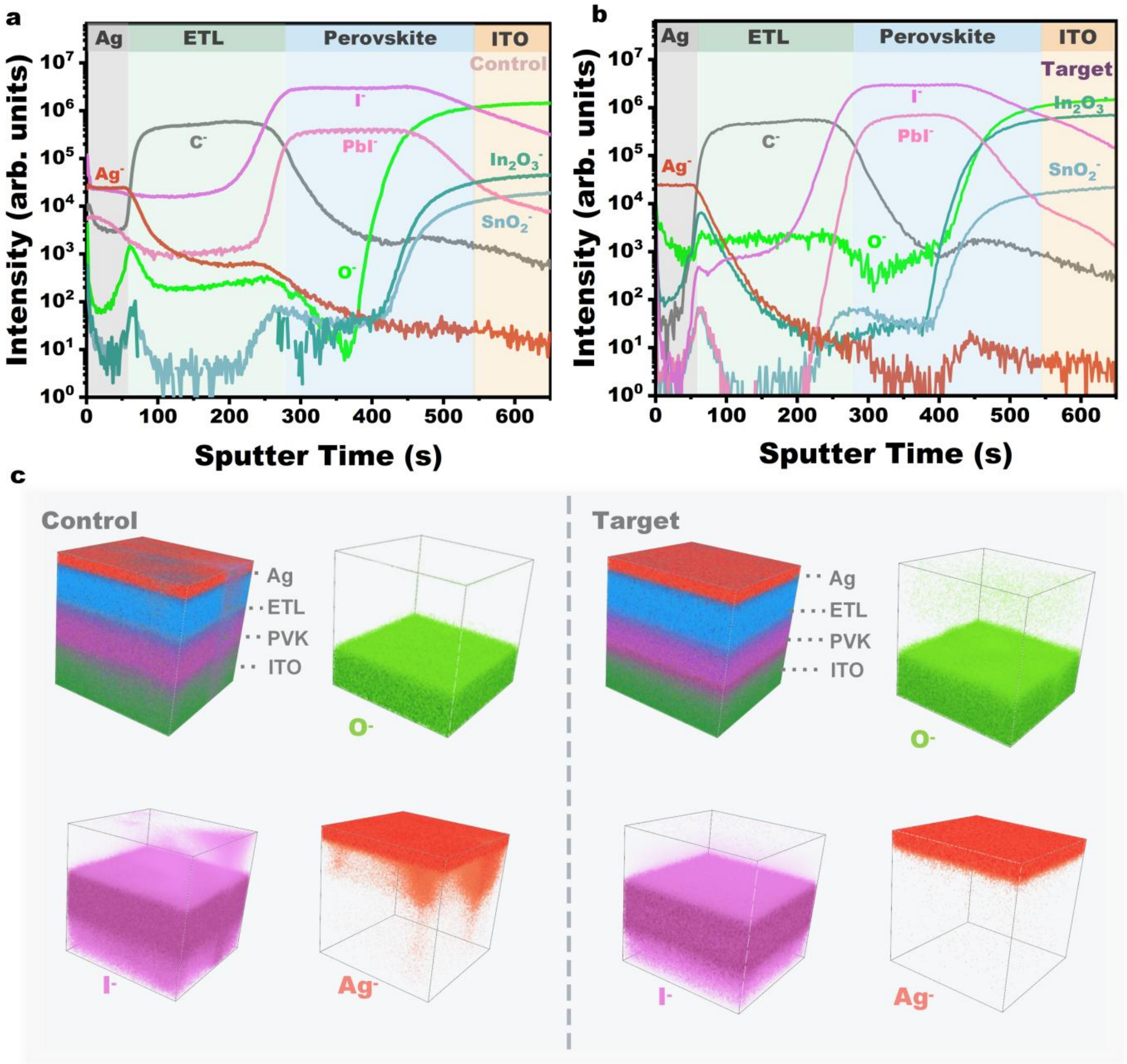

Figure S29. Time-of-light secondary-ion mass spectrometry (ToF-SIMS) depth profiles after 100 hours MPP tracking. (a) Control device. (b) Target device. (c) Corresponding ToF-SIMS 3D tomography results for control and target devices.

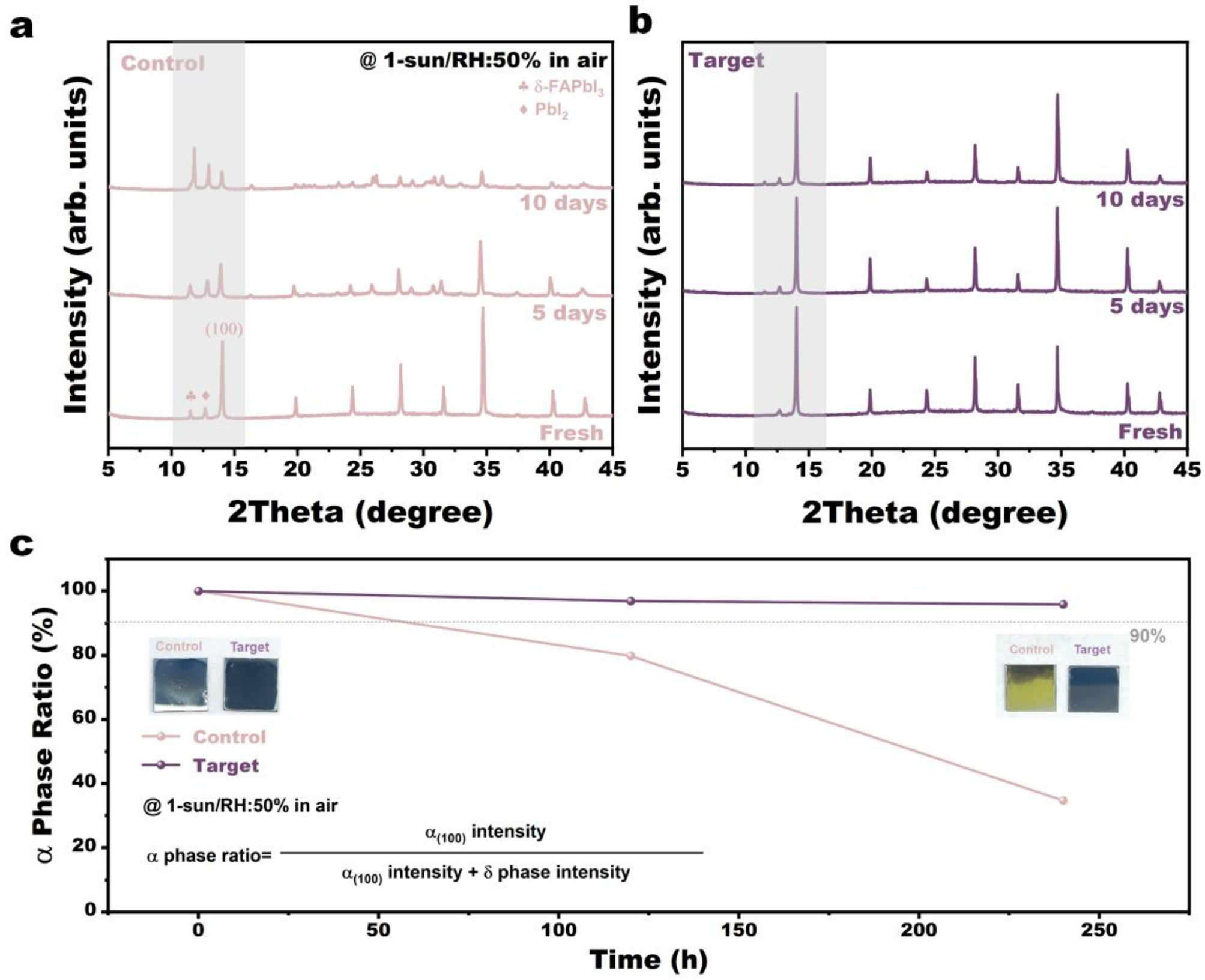

Figure S30. XRD patterns of the perovskite films during the accelerated aging process (air, 1-sun, 50% RH). (a) Control sample. (b) Target sample. (c) The corresponding α-phase (100) intensity ratio estimated by the equation $[\alpha_{(100)}]/[\alpha_{(100)} + \delta\text{-phase}]$, inset: photographs of the control and target perovskite films aged for different times, left (fresh), right(10 days).

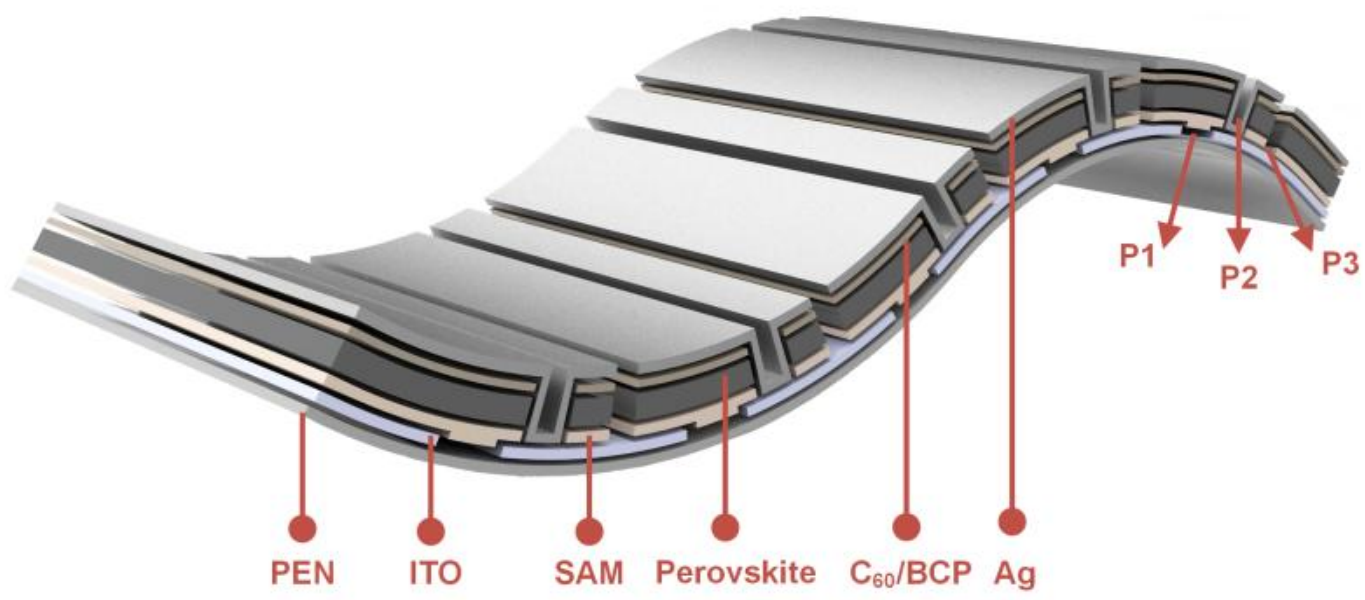

Figure S31. Schematic diagram of the minimodule's structure.

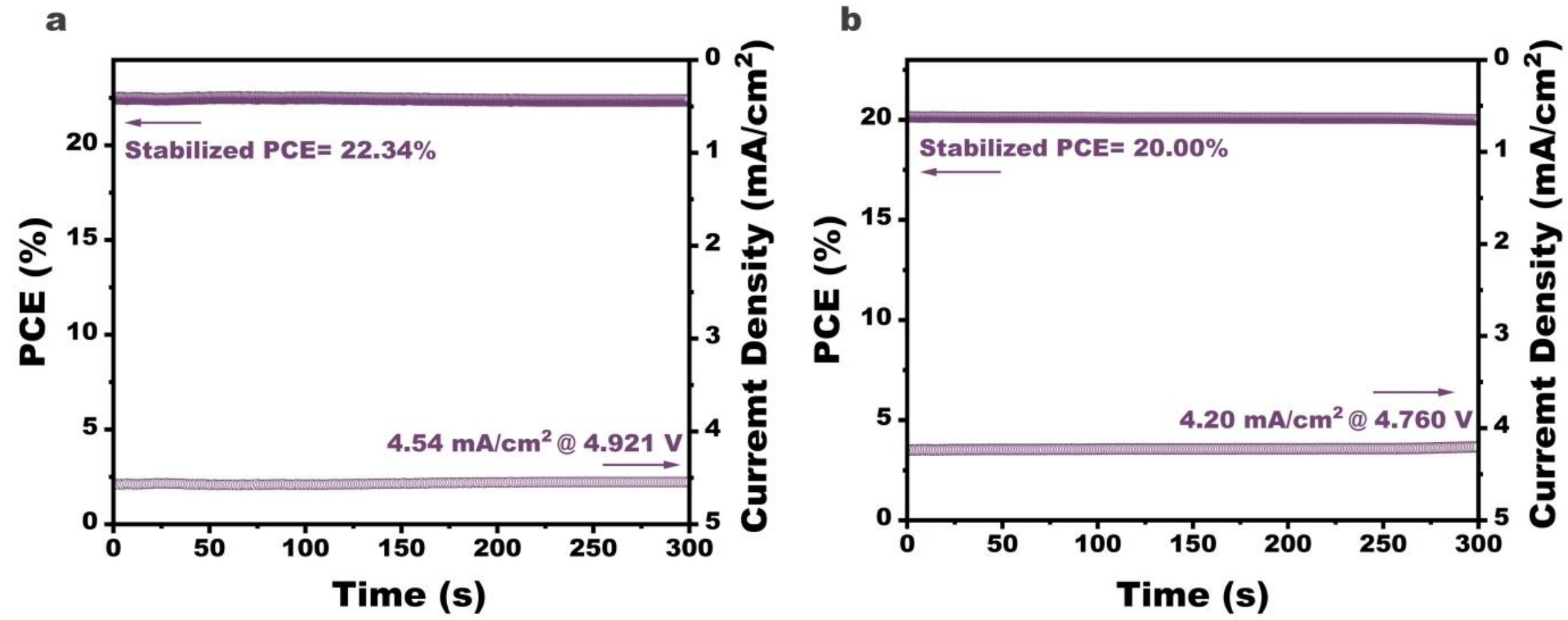

Figure S32. The stable power output and steady-state current density of the best-performance rigid and flexible PSMs.

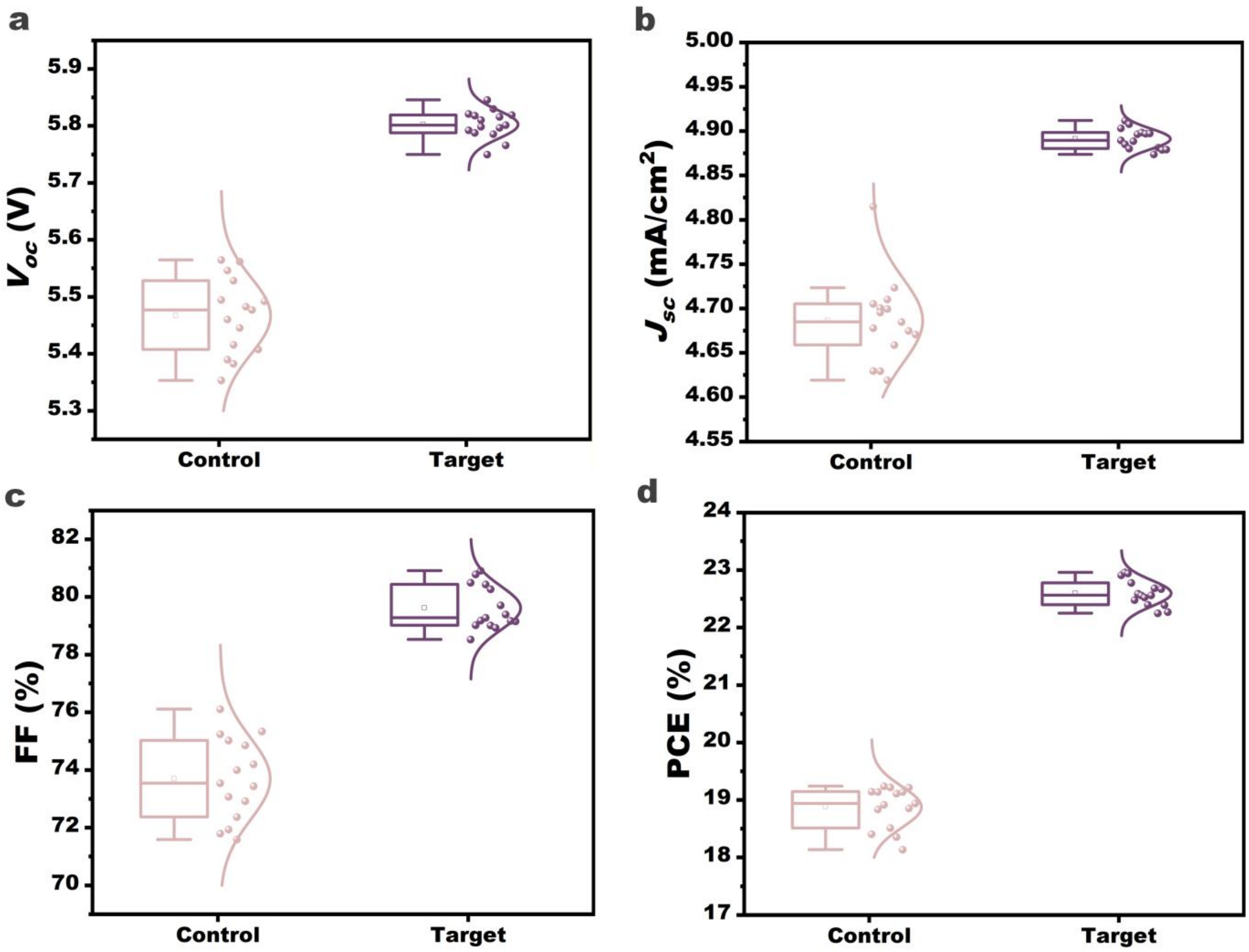

Figure S33. Statistical comparison of control and target-based perovskite mini-module with aperture area 10.24 cm^2 (the statistical results are obtained from 15 rigid mini-modules for each condition). (a) V_{OC} normal distribution. (b) J_{SC} normal distribution. (c) FF normal distribution. (d) PCE normal distribution. The box-and-whisker plot shows the distribution of data. The minimum value is indicated by the lower whisker, whereas the maximum value is shown by the upper whisker. The box represents the interquartile range, with the first quartile at the lower edge, the median as the line inside the box and the third quartile at the upper edge. The central line of the box plot represents the media, and the open square represents the mean value. Outliers are represented as individual points beyond the whiskers.

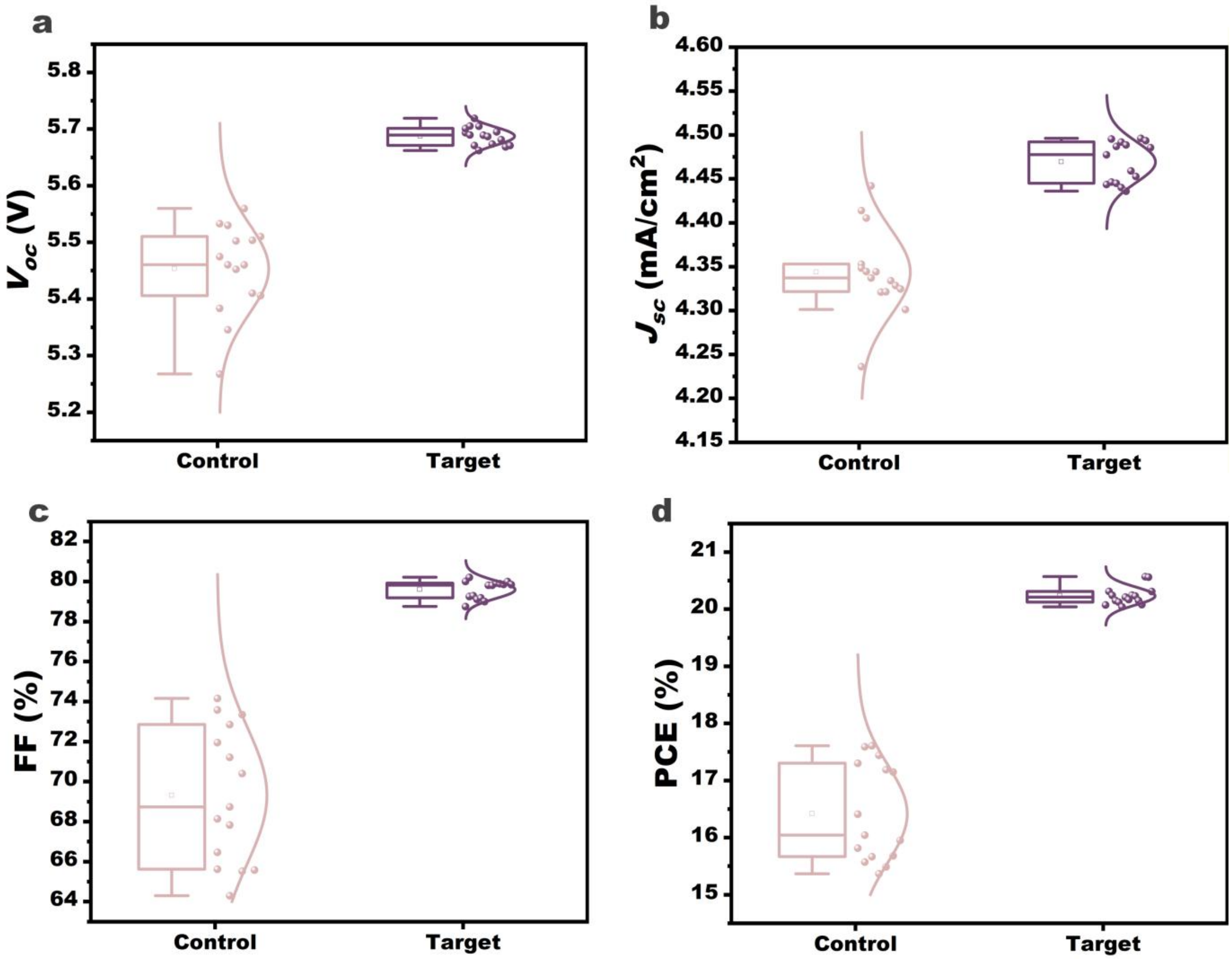

Figure S34. Statistical comparison of control and target-based perovskite mini-module with aperture area 10.24 cm^2 (the statistical results are obtained from 15 flexible mini-modules for each condition) (a) V_{OC} normal distribution. (b) J_{SC} normal distribution. (c) FF normal distribution. (d) PCE normal distribution. The box-and-whisker plot shows the distribution of data. The minimum value is indicated by the lower whisker, whereas the maximum value is shown by the upper whisker. The box represents the interquartile range, with the first quartile at the lower edge, the median as the line inside the box and the third quartile at the upper edge. The central line of the box plot represents the media, and the open square represents the mean value. Outliers are represented as individual points beyond the whiskers.

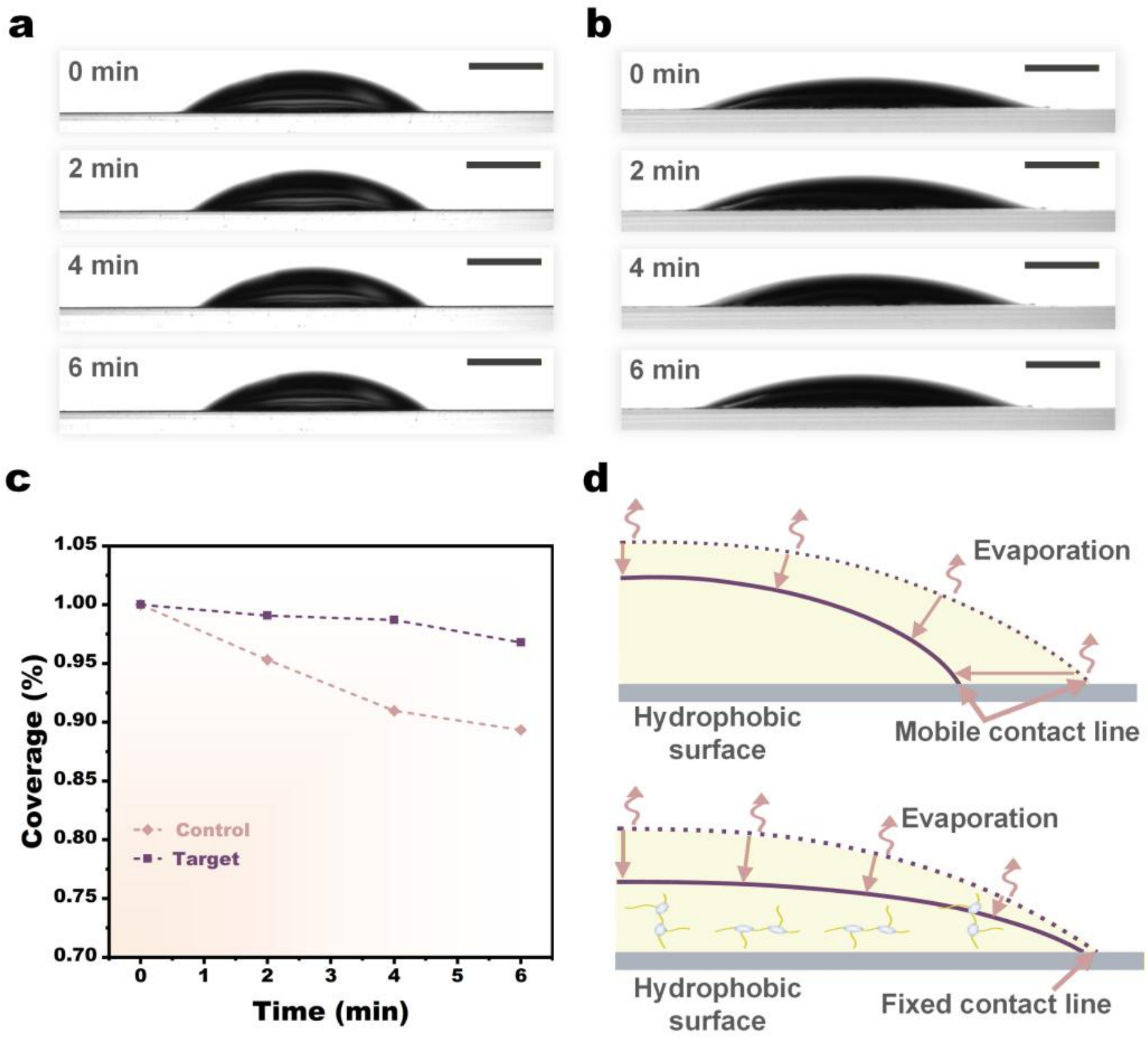

Figure S35. Comparison of droplet drying dynamics and surface coverage on hydrophobic SAM-based substrates. (a, b) Time-lapse optical images capturing the drying process of perovskite ink droplets on hydrophobic 4PADCB-covered substrates under ambient conditions: (a) control, (b) target. (c) Evolution of the normalized surface coverage of perovskite droplets as a function of drying time. (d) Schematic illustration for the shrinkage of the ink droplet drying on a hydrophobic substrate.

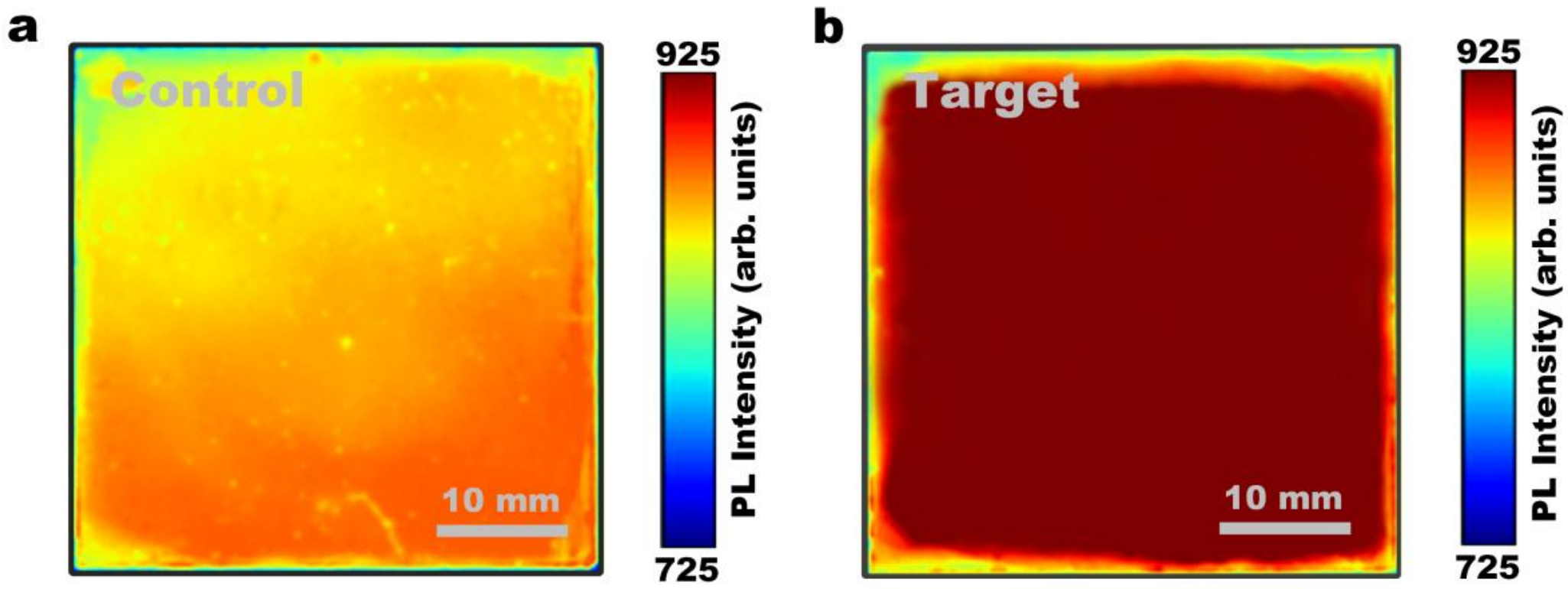

Figure S36. PL mapping images of perovskite films spin-coated on 50×50 mm^2 substrate. (a) Control sample, (b) Target sample.

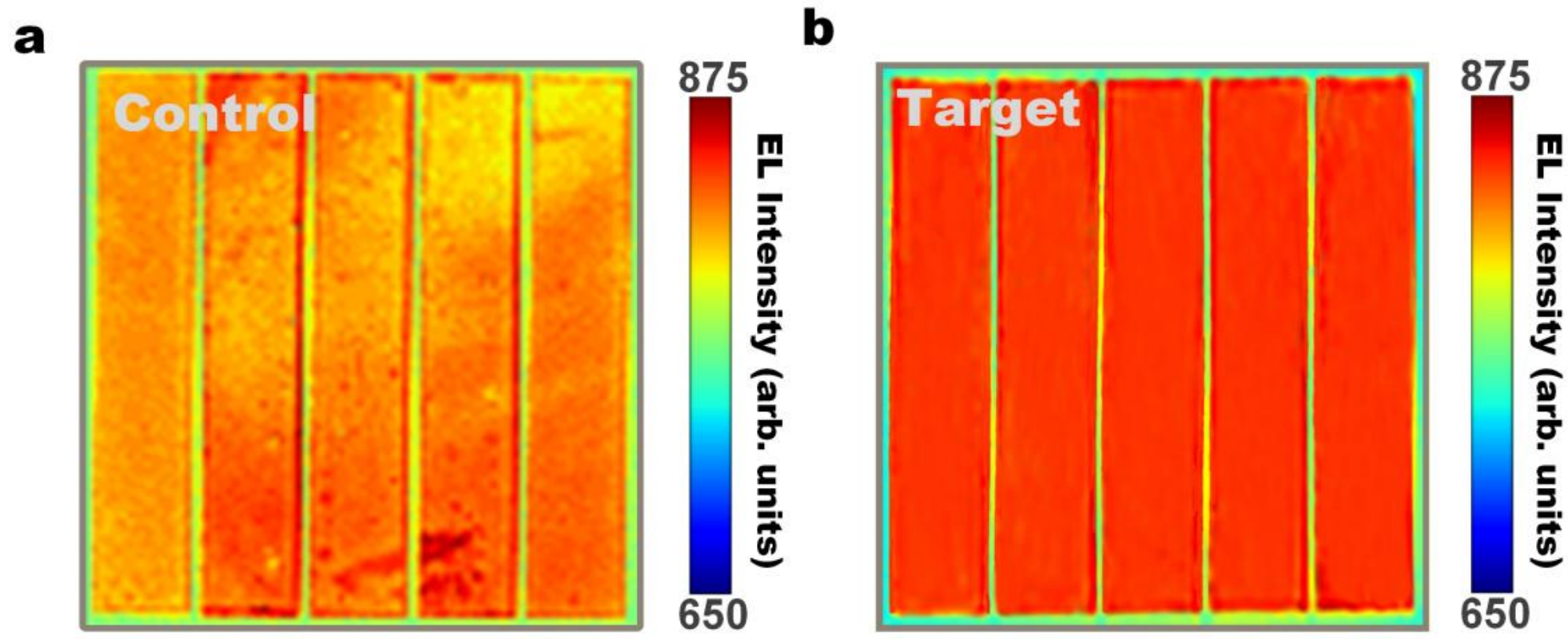

Figure S37. EL imaging of the (a) control and (b) target minimodules.

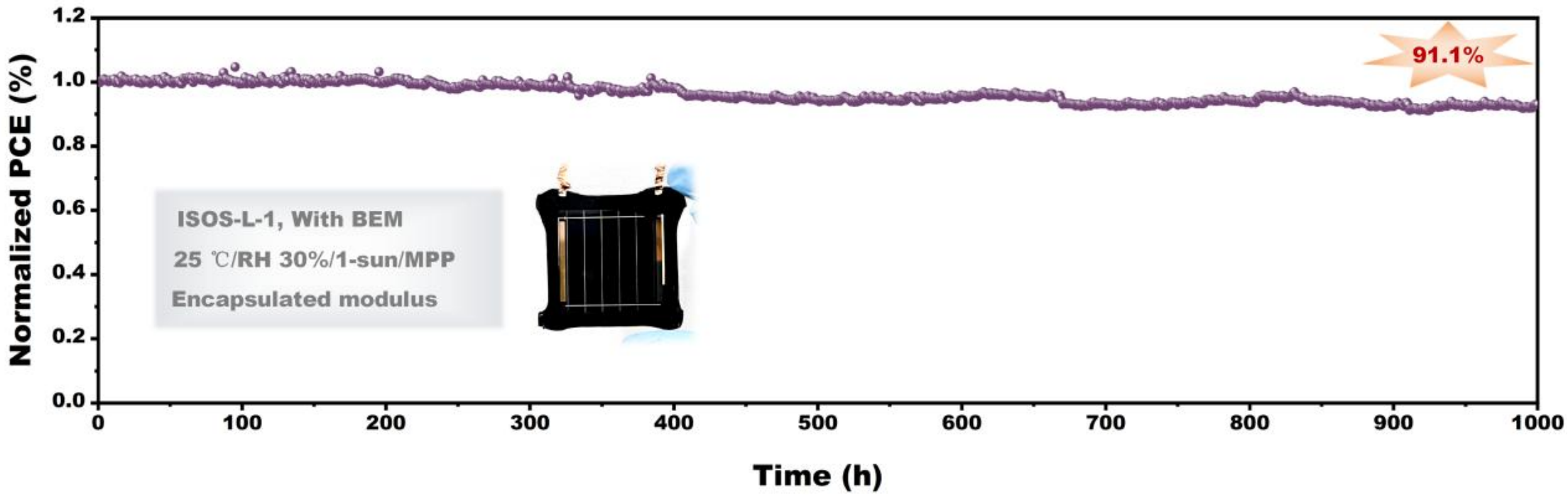

Figure S38. Stability of target module during MPP tracking measurement with 100 mW/cm^2 LED continuous illumination, inset: the encapsulated module.

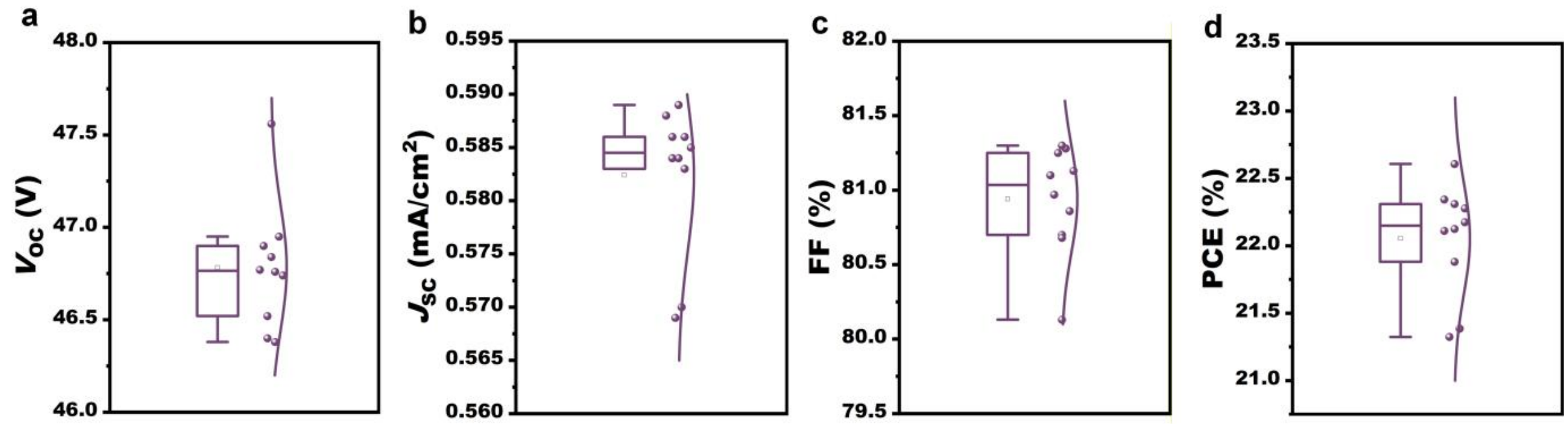

Figure S39. Statistical distribution of photovoltaic parameters for ten independent 30 × 30 cm^2 target submodules. The box-and-whisker plot shows the distribution of data. The minimum value is indicated by the lower whisker, whereas the maximum value is shown by the upper whisker. The box represents the interquartile range, with the first quartile at the lower edge, the median as the line inside the box and the third quartile at the upper edge. The central line of the box plot represents the media, and the open square represents the mean value. Outliers are represented as individual points beyond the whiskers.

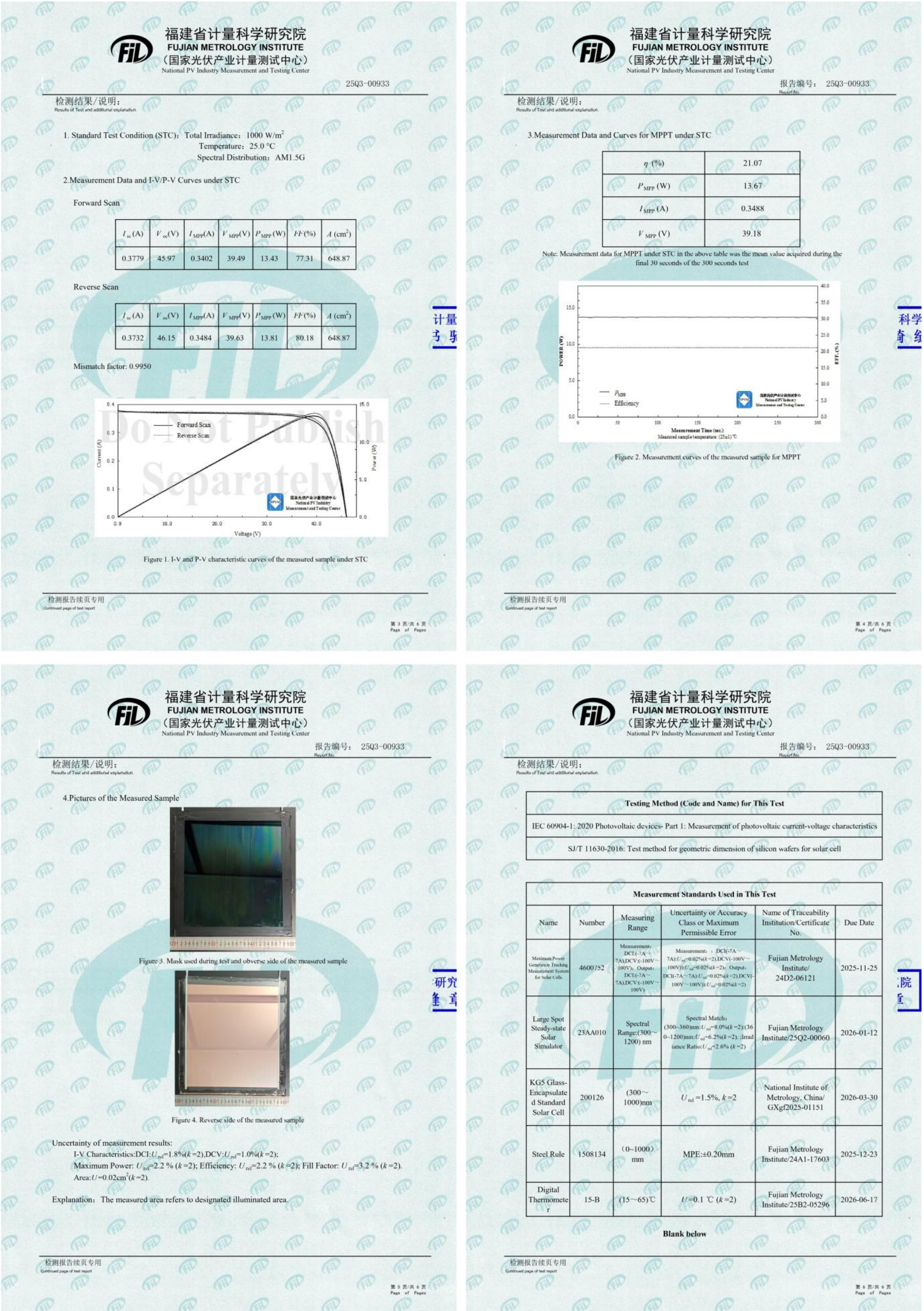

福建省计量科学研究院
FUJIAN METROLOGY INSTITUTE
（国家光伏产业计量测试中心）
National PV Industry Measurement and Testing Center

25Q3-00933

检测结果/说明：
Results of Test and additional explanation

1. Standard Test Condition (STC)：Total Irradiance：1000 W/m²
Temperature：25.0 °C
Spectral Distribution：AM1.5G

2.Measurement Data and I-V/P-V Curves under STC

Forward Scan

I_{sc} (A)	V_{oc} (V)	I_{MPP} (A)	V_{MPP} (V)	P_{MPP} (W)	FF (%)	A (cm²)
0.3779	45.97	0.3402	39.49	13.43	77.31	648.87

Reverse Scan

I_{sc} (A)	V_{oc} (V)	I_{MPP} (A)	V_{MPP} (V)	P_{MPP} (W)	FF (%)	A (cm²)
0.3732	46.15	0.3484	39.63	13.81	80.18	648.87

Mismatch factor: 0.9950

Figure 1. I-V and P-V characteristic curves of the measured sample under STC

检测报告续页专用
Continued page of test report

第 3 页/共 6 页
Page of Pages

福建省计量科学研究院
FUJIAN METROLOGY INSTITUTE
（国家光伏产业计量测试中心）
National PV Industry Measurement and Testing Center

报告编号： 25Q3-00933
Report No.

检测结果/说明：
Results of Test and additional explanation

3.Measurement Data and Curves for MPPT under STC

η (%)	21.07
P_{MPP} (W)	13.67
I_{MPP} (A)	0.3488
V_{MPP} (V)	39.18

Note: Measurement data for MPPT under STC in the above table was the mean value acquired during the final 30 seconds of the 300 seconds test

Figure 2. Measurement curves of the measured sample for MPPT

检测报告续页专用
Continued page of test report

第 4 页/共 6 页
Page of Pages

福建省计量科学研究院
FUJIAN METROLOGY INSTITUTE
（国家光伏产业计量测试中心）
National PV Industry Measurement and Testing Center

报告编号： 25Q3-00933
Report No.

检测结果/说明：
Results of Test and additional explanation

4.Pictures of the Measured Sample

Figure 3. Mask used during test and obverse side of the measured sample

Figure 4. Reverse side of the measured sample

Uncertainty of measurement results:
I-V Characteristics:DCI:U_{rel}=1.8%(k=2),DCV:U_{rel}=1.0%(k=2);
Maximum Power: U_{rel}=2.2 % (k=2); Efficiency: U_{rel}=2.2 % (k=2); Fill Factor: U_{rel}=3.2 % (k=2).
Area:U=0.02cm²(k=2).

Explanation：The measured area refers to designated illuminated area.

检测报告续页专用
Continued page of test report

第 5 页/共 6 页
Page of Pages

福建省计量科学研究院
FUJIAN METROLOGY INSTITUTE
（国家光伏产业计量测试中心）
National PV Industry Measurement and Testing Center

报告编号： 25Q3-00933
Report No.

检测结果/说明：
Results of Test and additional explanation

Testing Method (Code and Name) for This Test
IEC 60904-1: 2020 Photovoltaic devices- Part 1: Measurement of photovoltaic current-voltage characteristics
SJ/T 11630-2016: Test method for geometric dimension of silicon wafers for solar cell

Measurement Standards Used in This Test

Name	Number	Measuring Range	Uncertainty or Accuracy Class or Maximum Permissible Error	Name of Traceability Institution/Certificate No.	Due Date
Maximum Power Generation Tracking Measurement System for Solar Cells	4600752	Measurement: DCI:(-7A~7A),DCV:(-100V~100V); Output: DCI:(-7A~7A),DCV:(-100V~100V)	Measurement: : DCI(-7A~7A):U_{rel}=0.02%(k=2),DCV(-100V~100V):U_{rel}=0.02%(k=2); Output: DCI(-7A~7A):U_{rel}=0.02%(k=2),DCV(-100V~100V):U_{rel}=0.02%(k=2)	Fujian Metrology Institute/ 24D2-06121	2025-11-25
Large Spot Steady-state Solar Simulator	23AA010	Spectral Range:(300~1200) nm	Spectral Match: (300~360)nm:U_{rel}=8.0%(k=2):(360~1200)nm:U_{rel}=6.2%(k=2); ;Irradiance Ratio:U_{rel}=2.6% (k=2)	Fujian Metrology Institute/25Q2-00060	2026-01-12
KG5 Glass-Encapsulated Standard Solar Cell	200126	(300~1000)nm	U_{rel} =1.5%, k=2	National Institute of Metrology, China/ GXgf2025-01151	2026-03-30
Steel Rule	1508134	(0~1000) mm	MPE:±0.20mm	Fujian Metrology Institute/24A1-17603	2025-12-23
Digital Thermometer	15-B	(15~65)℃	U=0.1 ℃ (k=2)	Fujian Metrology Institute/25B2-05296	2026-06-17

Blank below

检测报告续页专用
Continued page of test report

第 6 页/共 6 页
Page of Pages

Figure S40. Certification report of the 30×30 cm² submodule.

Table S1. Contact angles and surface tension of control and perovskite precursor with BFDGE, IPDA, and BFDGE + IPDA.

	Control	W/BFDGE	W/IPDA	Target
θ (°)	29.5	26.0	23.0	16.2
γ (mN/m)	35.0	33.3	31.9	28.5

Table S2. Fitting carrier lifetimes of perovskite films determined by TRPL.

Sample	A_1	τ_1 (ns)	A_2	τ_2 (ns)	τ_{ave} (ns)
Control	0.41	165.36	0.72	1283.77	1207.50
Target	0.13	201.92	0.89	1929.79	1904.55

Table S3. R_1 and R_2 values from electrochemical impedance spectroscopy (EIS) data of devices.

Sample	$R_1(\Omega)$	$R_2(\Omega)$
Control	43.5	1173
Target	26.3	3377

Table S4. Results derived from electronic-only SCLC

Sample	V_{TFL} (V)	N_t (cm^{-3})
Control	0.72	1.07×10^{16}
Target	0.42	6.24×10^{15}

Table S5. The dependence of V_{OC} and J_{SC} of the f-PSCs on light intensity

Sample	nk_BT/q	α
Control	$1.43k_BT/q$	0.972
Target	$1.29k_BT/q$	0.998

Table S6. Statistics of photovoltaic parameters for 30 individual rigid PSCs.

Sample	V_{OC} (V)	J_{SC} (mA/cm^2)	FF (%)	PCE (%)
Control	1.127±0.012	25.35±0.42	81.9±1.19	23.41±0.48
Target	1.186±0.002	26.02±0.19	84.6±0.39	26.10±0.22

Table S7. Statistics of photovoltaic parameters for 30 individual flexible PSCs.

Sample	V_{OC} (V)	J_{SC} (mA/cm^2)	FF (%)	PCE (%)
Control	1.109±0.012	24.69±0.37	79.2±1.37	21.68±0.40
Target	1.163±0.005	25.39±0.23	83.2±0.71	24.56±0.27

Table S8. Photovoltaic parameters of the rigid mini-modules in Figure 6a.

Sample	V_{OC} (V)	J_{SC} (mA/cm^2)	FF (%)	PCE (%)
Control	5.46	4.63	76.1	19.24
Target	5.99	4.92	78.9	23.25

Table S9. Photovoltaic parameters of the flexible mini-modules in Figure 6b.

Sample	V_{OC} (V)	J_{SC} (mA/cm^2)	FF (%)	PCE (%)
Control	5.48	4.34	74.1	17.61
Target	5.72	4.50	80.0	20.59

Table S10. Statistics of photovoltaic parameters for 15 individual rigid PSMs.

Sample	V_{OC} (V)	J_{SC} (mA/cm^2)	FF (%)	PCE (%)
Control	5.46±0.07	4.68±0.05	73.6±1.42	18.87±0.36
Target	5.80±0.02	4.89±0.01	79.6±0.76	22.60±0.23

Table S11. Statistics of photovoltaic parameters for 15 individual flexible PSMs.

Sample	V_{OC} (V)	J_{SC} (mA/cm^2)	FF (%)	PCE (%)
Control	5.45±0.08	4.34±0.05	69.3±3.39	16.41±0.86
Target	5.69±0.02	4.47±0.02	79.6±0.45	20.23±0.16

Table S12. Photovoltaic parameters for the champion 30 × 30 cm^2 submodules.

Sample	V_{OC} (V)	J_{SC} (mA/cm^2)	FF (%)	PCE (%)
Target	47.56	0.589	80.7	22.60

Table S13. Summary of photovoltaic parameters of reported high-performance inverted PSMs up to now (active area over 100 cm^2)

Device Architecture	V_{OC} (V)	J_{SC} (mA/cm^2)	FF (%)	PCE (%)	Area (cm^2)	Subcell (unit)	Ref.
Glass/FTO/NiO_x/SAM/Al_2O_3/Perovskite/C60/BCP/Cu	16.13	1.593	78.4	20.14	113	14	[1]
Glass/FTO/NiO_x/Perovskite/PCBM/BCP/Ag	20.74	1.143	78.4	18.6	174	20	[2]
Glass/FTO/NiO_x(Urea)/Perovskite/BzMIMBr/C60/BCP/Cu	19.84	1.16	74.7	17.18	178.4	19	[3]
Glass/ITO//NiO_x/SAM/Perovskite/NH_4F/$PC_{61}BM$/BCP/Ag	21.9	1.02	76.0	18.1	228	20	[4]
Glass/FTO/SAM/Perovskite/Y7-BO/PCBM/BCP/Ag	47.12	6.17	76.8	22.32	400	-	[5]
Glass/FTO/NiO_x/SAM/Perovskite/LiF/C_{60}/BCP/Ag	-	-	-	19.25	641.4	-	[6]
Glass/ITO/NiO_x/SAM/Al_2O_3/Perovskite/PCBM/BCP/Ag	38.25	0.623	77.8	18.54	642	38	[7]
Glass/FTA/NiO_x/Perovskite/PHDFA/PCBM/SnO_2/ITO/Cu/ITO	38.84	0.626	74.8	18.20	642	-	[8]
Glass/ITO/NiO_x/SAM/Perovskite/LiF/C_{60}/SnO_2/Cu	45.02	0.56	79.5	20.06	651.42	-	[9]
Glass/ITO/NiO_x/PTAA/AlO_X/Perovskite/PCBM/BCP/Ag	37.06	0.679	71.1	17.05	669	-	[10]
Glass/ITO/NiO_x/SAM/Perovskite/PCBM/C_{60}/SnO_2/Cr/Ag	50.1	0.547	77.0	21.1	730	46	[11]
**Glass/ITO/NiO_x/SAM/Perovskite/C_{60}/BCP/Ag**	**47.56**	**0.589**	**80.7**	**22.60**	**655.2**	**42**	**This work**

Table S14. Statistics of photovoltaic parameters for 10 individual 30 × 30 cm^2 submodules.

Sample	V_{OC} (V)	J_{SC} (mA/cm^2)	FF (%)	PCE (%)
Target	46.78±0.34	0.582±0.007	80.94±0.36	20.05±0.41

Supplementary Reference

1. Liu, W. *et al.* Buried Interface Engineering for Scalable Processing of High-Performance Inverted Perovskite Solar Modules. *Adv. Energy Mater.* **15***,* 2404374 (2024).
2. Du, M. *et al.* Surface redox engineering of vacuum-deposited NiOx for top-performance perovskite solar cells and modules. *Joule* **6**, 1931-1943 (2022).
3. Li, C. *et al.* Efficient Inverted Perovskite Solar Cells with a Fill Factor Over 86% via Surface Modification of the Nickel Oxide Hole Contact. *Adv. Funct. Mater.* **33**, 2214774 (2023).
4. Zhao, X. *et al.* Operationally stable perovskite solar modules enabled by vapor-phase fluoride treatment. *Science* **385**, 433–438 (2024).
5. Liu, Q. *et al.* Enhancing the Efficiency and Stability of Inverted Perovskite Solar Cells and Modules through Top Interface Modification with N-type Semiconductors. *Angew. Chem. Int. Ed.* **137**, e202416390 (2025).
6. Guo, H. *et al.* Intermolecular interactions triggered crystallization phase transition regulation for efficient and stable perovskite photovoltaics. *Energy Environ. Sci.* **18**, 7114-7123 (2025).
7. Wang, K. *et al.* Amidino-based ligand enables oriented crystallization and boosted carrier extraction for inverted perovskite solar cells and modules. *Joule* **9**, 102044 (2025).
8. Xu, Z. *et al.* Double amidino-mediated multiple hydrogen-bonded Dion–Jacobson perovskites enable oriented crystallization for efficient inverted $FAPbI_3$ solar cells and modules (642 cm^2). *Energy Environ. Sci.* **18**, 8268-8279 (2025).
9. Yang, H. *et al.* Molecular Extrusion Drives Polymer Dynamic Soft Encapsulation to Inhibit Lead Leakage for Efficient Inverted Perovskite Solar Cells and Modules. *Adv. Mater.*, e11855 (2025) doi.org/10.1002/adma.202511855.
10. Gong, C. *et al.* Efficient and stable inverted perovskite solar cells enabled by homogenized PCBM with enhanced electron transport. *Nat. Commun.* **15**, 9154 (2024).
11. Sun, X. *et al.* Vapor-assisted surface reconstruction enables outdoor-stable perovskite solar modules. *Science*, **388**, 957-963 (2025).
