# Supplementary material for: Stepwise polymerization enables tailored perovskite ink for scalable flexible perovskite solar cells
Source: Nat Commun. 2026 Sep 15;17:10858. doi: 10.1038/s41467-026-77703-w (PMC13672371; doi:10.1038/s41467-026-77703-w)
Supplement: Supplementary file 2 [file 41467_2026_77703_MOESM2_ESM.pdf]

nature portfolio

Corresponding author(s): Ziyi Ge

Solar Cells Reporting Summary

Nature Portfolio wishes to improve the reproducibility of the work that we publish. This form is intended for publication with all accepted papers reporting the characterization of photovoltaic devices and provides structure for consistency and transparency in reporting. Some list items might not apply to an individual manuscript, but all fields must be completed for clarity.

For further information on Nature Research policies, including our data availability policy, see Authors & Referees.

• Experimental design

Please check the following details are reported in the manuscript, and provide a brief description or explanation where applicable.

1. Dimensions

Item	Yes/No	Response
Area of the tested solar cells	[x] Yes	0.06 cm2
	[ ] No	*Explain why this information is not reported/not relevant.*
Method used to determine the device area	[x] Yes	The active area was determined by the aperture shade mask (0.06 cm2) placed infront of the solar cell
	[ ] No	*Explain why this information is not reported/not relevant.*

2. Current-voltage characterization

Item	Yes/No	Response
Current density-voltage (J-V) plots in both forward and backward direction	[x] Yes	Shown in the Figures 5b and 5c in the manuscript
	[ ] No	
Voltage scan conditions	[x] Yes	Bias voltage for J-V measurements for PSCs were scanned from 1.2V to -0.2 V and from -0.2V to 1.2V with a scanning rate of 100 mV/s
	[ ] No	*Explain why this information is not reported/not relevant.*
Test environment	[x] Yes	The J-V curves were tested in a N2-filled glovebox and room temperature, no humidity
	[ ] No	*Explain why this information is not reported/not relevant.*
Protocol for preconditioning of the device before its characterization	[ ] Yes	*Provide a description of the protocol.*
	[x] No	No preconditioning is needed
Stability of the J-V characteristic	[x] Yes	Shown in the Figure S24
	[ ] No	*Explain why this information is not reported/not relevant.*

3. Hysteresis or any other unusual behaviour

Item	Yes/No	Response
Description of the unusual behaviour observed during the characterization	[ ] Yes	*Provide a description of hysteresis or any other unusual behaviour observed during the characterization.*
	[x] No	Very minor hysteresis was observed in the single-junction device
Related experimental data	[x] Yes	Shown in Figure S23
	[ ] No	*Explain why this information is not reported/not relevant.*

4. Efficiency

Item	Yes/No	Response
External quantum efficiency (EQE) or incident photons to current efficiency (IPCE)	[x] Yes	Figure S27
	[ ] No	*Explain why this information is not reported/not relevant.*
A comparison between the integrated response under the standard reference spectrum and the response measure under the simulator	[x] Yes	The deviations were less than 5%
	[ ] No	*Explain why this information is not reported/not relevant.*

For tandem solar cells, the bias illumination and bias voltage used for each subcell

- [ ] Yes — *Provide a description of the measurement conditions.*
- [x] No — *Explain why this information is not reported/not relevant.*

5. Calibration

Light source and reference cell or sensor used for the characterization

- [x] Yes — PVM1121,mono-Si, WPVS
- [ ] No — *Explain why this information is not reported/not relevant.*

Confirmation that the reference cell was calibrated and certified

- [x] Yes — Calibrated by NREL (Certificate No.ISO 2075)
- [ ] No — *Explain why this information is not reported/not relevant.*

Calculation of spectral mismatch between the reference cell and the devices under test

- [ ] Yes — *Provide a value of the spectral mismatch and/or a description of how it has been taken into account in the measurements.*
- [ ] No — *Explain why this information is not reported/not relevant.*

6. Mask/aperture

Size of the mask/aperture used during testing

- [x] Yes — 0.06 cm2
- [ ] No — *Explain why this information is not reported/not relevant.*

Variation of the measured short-circuit current density with the mask/aperture area

- [ ] Yes — *Report the difference in the short-circuit current density values measured with the mask and aperture area.*
- [x] No — All J-V were measured with a black metal aperture mask

7. Performance certification

Identity of the independent certification laboratory that confirmed the photovoltaic performance

- [ ] Yes — *Identify the independent certification laboratory.*
- [x] No — *Explain why this information is not reported/not relevant.*

A copy of any certificate(s)

- [ ] Yes — *Certificate copies should be provided in the Supplementary information. Please state the supplementary item number.*
- [x] No — *Explain why this information is not reported/not relevant.*

8. Statistics

Number of solar cells tested

- [x] Yes — 30
- [ ] No — *Explain why this information is not reported/not relevant.*

Statistical analysis of the device performance

- [x] Yes — Figures S25, S26
- [ ] No — *Explain why this information is not reported/not relevant.*

9. Long-term stability analysis

Type of analysis, bias conditions and environmental conditions

- [x] Yes — Figures 5g and 5h
- [ ] No — *Explain why this information is not reported/not relevant.*
