# Supplementary material for: Stepwise polymerization enables tailored perovskite ink for scalable flexible perovskite solar cells
Source: Nat Commun. 2026 Sep 15;17:10858. doi: 10.1038/s41467-026-77703-w (PMC13672371; doi:10.1038/s41467-026-77703-w)
Supplement: Supplementary file 3 [file 41467_2026_77703_MOESM3_ESM.pdf]

nature portfolio

Peer Review File

Stepwise Polymerization Enables Tailored Perovskite Ink for Scalable Flexible Perovskite Solar Cells

Corresponding Author: Professor Ziyi Ge

This file contains all reviewer reports in order by version, followed by all author rebuttals in order by version.

Version 0:

Reviewer comments:

Reviewer #1

(Remarks to the Author)
The manuscript by Zhang et al, present a study on the stepwise polymerization between bisphenol F diglycidyl ether (BFDGE) and isophorone diamine (IPDA) for enhanced wettability of perovskite ink on hydrophobic self-assembling molecules. The tailored perovskite precursor exhibits improved surface coverage and assists perovskite crystal growth during the annealing process. In addition, the resulting crosslinked scaffold could serve as a bulk encapsulation medium (BEM), enabling enhanced operational stability and bending stability of flexible perovskite solar cells. Although comprehensive characterizations are provided to explain the enhancement of performance and stability, there are still some aspects to be investigated with deeper depth. Herein, I recommend this manuscript to be accepted after addressing the following issues:

(1) Given that the focus of the manuscript is the enhanced interfacial contact through tailored perovskite ink achieved by programmable polymerization, I suggest reinforcing this conceptual advance with additional experimental evidence. Specifically, contact angle and surface energy analysis should be performed to distinguish the effects of the monomer (BFDGE), the curing agent (IPDA), and the final polymer. This is fundamental to a mechanistic understanding.

(2) The authors demonstrate improved film coverage through optical photographs (e.g., on 5×5 cm2 substrates) and provide surface-level characterizations, however, a direct investigation of the perovskite/SAMs interface is crucial to elucidate the fundamental mechanism. I suggest probing the buried interface using techniques like PL Mapping, TRPL, SEM, etc. (see, e.g., Science, 390, 638-642 (2025); Science，391, eadz7969 (2026)) to provide direct mechanistic insight into how the programmable polymerization promotes scalable fabrication.

(3) The manuscript claims that “the resulting polymer with hydrophobic nature can serve as a bulk encapsulation medium (BEM), which will enhance the moisture resistance and overall stability of the devices.” However, the sole MPPT tracking test over 1000 hours primarily assesses operational stability under continuous illumination and electrical bias, which is insufficient to substantiate specific claims regarding moisture resistance or the encapsulation function. We suggest that the authors reconsider this part of their statement, or provide targeted evidence (e.g., damp-heat testing) for the specific moisture-resistance and encapsulation properties.

(4) The shifts in XPS peaks (Pb 4f, N 1s, I 3d) to lower binding energy in Figures 1C-E provide compelling evidence for surface chemical passivation by the BEM. However, as XPS is a surface-sensitive technique, it cannot assess the bulk or depth distribution of the BEM. Therefore, time of light secondary ion mass spectroscopy (TOF-SIMS) measurements are recommended to monitor the spatial distribution of BEM, which is beneficial for investigating the detailed influence of the additive on the perovskite film.

(5) It would be beneficial if the authors could provide more details on the fabrication process of perovskite solar submodules to enhance reproducibility for other research groups.

(6) Despite the impressive device performance achieved by the authors, I would like to raise a question regarding the significant performance gap between the rigid and flexible devices. Could the authors provide further explanation for this discrepancy?

Reviewer #2

(Remarks to the Author)
This manuscript presents a programmable polymerization strategy using BFDGE and IPDA to tailor perovskite ink for scalable flexible perovskite solar cells (f-PSCs). The authors report a good large-area module performance. While the work is technically sound, several critical concerns preclude its publication in its current form. However, the novelty of the present work is limited which does not meet the publication requirement of this journal.
Comments:
1.The concept of using cross-linkable monomers or polymerizable additives to improve perovskite film quality, stability, and scalability has been extensively reported in recent literature (e.g., Refs. 19–21, 29, 41). The authors' approach, while well-executed, does not introduce a fundamentally new mechanism or material paradigm. The use of epoxy-amine chemistry, though effective, has been applied in other contexts. The incremental advance does not meet the threshold of transformative or groundbreaking research required for publication in a high-impact journal.
2.The proposed "programmable" nature of the polymerization is not clearly demonstrated or quantified. The term "programmable" suggests tunable or sequential control, but the presented data only shows a standard stepwise epoxy-amine reaction synchronized with annealing.
3.The distribution and role of the BEM (bulk encapsulation medium) at grain boundaries, while shown via EDX and HRTEM, lacks direct evidence of its dynamic interaction with perovskite crystallization in real time.
4.Claims regarding strain relaxation and ion migration suppression are supported indirectly (e.g., GIXRD, ToF-SIMS), but a more direct, in-situ or operando study linking BEM distribution to mechanical and ionic behavior is missing.
5.The study lacks a thorough analysis of potential drawbacks, such as the effect of BEM on charge transport across grain boundaries, long-term chemical stability of the polymer under UV exposure, or possible interfacial degradation during thermal cycling.
6.The use of "record-breaking" and "new benchmark" for the 655.2 cm^2 module is not sufficiently contextualized within the broader landscape of large-area perovskite modules, especially considering recent reports of similar or higher efficiencies on comparable areas.

Reviewer #3

(Remarks to the Author)
In this manuscript, Zhang et al. report a programmable polymerization strategy that precisely controls the wettability of perovskite precursor inks on hydrophobic self-assembled monolayers (SAMs). This enables reliable, large-area deposition of high-quality perovskite films while significantly reducing substrate dependence. The strategy not only enhances the performance and operational stability of small-area devices, but also facilitates remarkable progress in scalable fabrication. The reported power conversion efficiencies are at the forefront of the field: 27.16% (certified 26.84%) for small-area devices and 22.60% (certified 21.07%) for 655.2 cm^2 submodules. This work offers valuable fundamental mechanistic insights alongside compelling technological advances. I recommend its publication in Nature Communications after the following points are addressed:
1.The authors discuss the impact of programmable polymerization on the surface morphology of perovskite films. To further support the claim of reliable ink deposition on hydrophobic SAM-based substrates, I recommend providing additional cross-sectional SEM images of the buried interface under various treatment conditions. This would offer more direct insight into how programmable polymerization governs the overall film formation process.
2.The manuscript demonstrates the effectiveness of the programmable polymerization strategy on 4PADCB SAMs. To better assess its broader applicability, it would be valuable to test whether the tailored perovskite ink can also yield high-quality, uniform films on other types of SAMs.
3.The authors state that programmable polymerization enables tailored perovskite ink. In the context of scalable fabrication, the adhesion between the perovskite ink and the underlying hydrophobic SAM layer is of great importance. Please provide supporting evidence on this aspect.
4.The mechanism underlying the N-type doping effect induced by the BEM, mentioned on Page 15, should be clarified.
5.The authors attribute the enhanced VOC primarily to the suppression of non-radiative recombination at the perovskite/ETL interface via the programmable polymerization strategy. However, drawing this specific conclusion directly from the results in Figure 5F may not be fully rigorous. The authors should reconsider this interpretation and provide additional explanatory evidence.

Version 1:

Reviewer comments:

Reviewer #1

(Remarks to the Author)
The authors have adequately addressed all reviewer comments, and the manuscript is now suitable for acceptance.

Reviewer #2

(Remarks to the Author)

This manuscript reports an in-situ stepwise polymerization strategy using BFDGE and IPDA to improve perovskite film quality, device efficiency, stability, and scalability. Although the work presents high device efficiencies and large-area module results, fundamental flaws in mechanism clarity, data consistency, control experiments, characterization logic, and novelty remain unresolved. The manuscript does not meet the rigorous standards for publication. It can be considered in other journals below the standards of NC.

1. I think that the authors failed to give a clear and proven “Stepwise Polymerization” mechanism. The authors claim a stepwise ring opening polymerization synchronized with perovskite crystallization, but provide no real time kinetic evidence. No in situ monitoring of polymerization rate, molecular weight evolution, or cross linking degree during annealing.The reaction between BFDGE and IPDA at 90–100 °C is too fast to be “stepwise”; the claimed “stepwise” control is speculative and unsupported. The authors cannot distinguish whether performance improvements come from polymer cross-linking, small molecule passivation, or solvent effects.
2. The critical control experiments are missing. No devices using pre polymerized BEM instead of in situ polymerization. No tests with BFDGE only or IPDA only at matched concentrations. No comparison with common cross-linkers to prove uniqueness of the BFDGE–IPDA system. No blank devices with equivalent DMF addition to rule out solvent effects.
3. Severe data Inconsistency and unreliability. Integrated J_{SC} from EQE does not closely match J_{SC} from J–V curves. Trap density decreases but carrier lifetime trends are over interpreted.YOY values and thermal stability data lack error bars and statistical repeatability.
4. Overclaimed Scalability and Module Fabrication. No uniformity mapping, no yield statistics, and no reproducibility data for 30×30 cm^2 modules.
5. HRTEM and EDX line scans do not convincingly prove BEM exists only at grain boundaries.
6. The novelty of this work is limited. As in situ polymerization, cross linking, and epoxy–amine chemistry for perovskites have been extensively reported. The authors do not highlight new scientific insights beyond prior work.

Reviewer #3

(Remarks to the Author)
All concerns have been addressed in the revised version，I recommend its publication in Nature Communications.

Version 2:

Reviewer comments:

Reviewer #2

(Remarks to the Author)
Zhang et al. proposed a stepwise polymerization strategy to notably improve the scalable reliability of perovskite solar cells (PSCs), which is a critical issue for the manufacturing of solar modules. The authors incorporated an epoxy-terminated monomer (BFDGE) and a curing agent (isophorone diamine, IPDA). The stepwise reaction between BFDGE and IPDA enabled a tailored perovskite ink at room temperature, which ensures the uniform and reliable fabrication of perovskite films across large-area hydrophobic substrates; moreover, thermal annealing synchronizes the polymerization with perovskite crystallization, dynamically modulating the crystallization process. Overall, this work successfully addresses a key challenge and provides a convincing story that will attract a broad interest in this field.

The previous reviewer’s critique of the ‘stepwise polymerization’ mechanism was well-founded, as claims of this nature in the literature often lack direct, fundamental data to substantiate them. The authors have executed a textbook revision. They did not merely offer verbal refutations; they performed extensive, well-designed experiments that directly transformed previous speculative claims into quantitative, verifiable physical insights. In-situ FTIR spectroscopy clearly validates the two-step reaction kinetics, while comprehensive control experiments confirm that the resulting polymer network, rather than individual additives, is uniquely responsible for the observed efficiency enhancement. This revised manuscript has been substantially improved, and the corrections requested by the reviewers have been exhaustively addressed. Hence, I believe that the present version is suitable for acceptance.

Open Access This Peer Review File is licensed under a Creative Commons Attribution 4.0 International License, which permits use, sharing, adaptation, distribution and reproduction in any medium or format, as long as you give appropriate credit to the original author(s) and the source, provide a link to the Creative Commons license, and indicate if changes were made.
In cases where reviewers are anonymous, credit should be given to 'Anonymous Referee' and the source.
The images or other third party material in this Peer Review File are included in the article's Creative Commons license, unless indicated otherwise in a credit line to the material. If material is not included in the article's Creative Commons license and your intended use is not permitted by statutory regulation or exceeds the permitted use, you will need to obtain permission directly from the copyright holder.
To view a copy of this license, visit https://creativecommons.org/licenses/by/4.0/

Dear Reviewers,

Thank you for your attention in handling our manuscript entitled "*Programmable Polymerization Enables Tailored Perovskite Ink for Scalable Flexible Perovskite Solar Cells*" (**Manuscript ID: NCOMMS-25-105245A**). We greatly appreciate your help in improving the paper. We have carefully revised the manuscript according to your comments, and the corresponding changes in the manuscript are presented in **blue font**. Our responses to your comments along with the revisions are included herewith. We hope that our manuscript now meets the requirements of **"*Nature Communications*"** as one of the field's leading journals.

If there are any other modifications you would recommend, we would like very much to further modify the manuscript, and we truly appreciate your help. We hope that our manuscript is considered favorably for prompt publication. Thank you very much once again for your help.

COMMENTS TO AUTHOR:

Reviewer: 1

Comments:

The manuscript by Zhang et al, present a study on the stepwise polymerization between bisphenol F diglycidyl ether (BFDGE) and isophorone diamine (IPDA) for enhanced wettability of perovskite ink on hydrophobic self-assembling molecules. The tailored perovskite precursor exhibits improved surface coverage and assists perovskite crystal growth during the annealing process. In addition, the resulting crosslinked scaffold could serve as a bulk encapsulation medium (BEM), enabling enhanced operational stability and bending stability of flexible perovskite solar cells. Although comprehensive characterizations are provided to explain the enhancement of performance and stability, there are still some aspects to be investigated with deeper depth. Herein, I recommend this manuscript to be accepted after addressing the following issues:

Author reply: Thank you for your positive evaluation and constructive feedback on our work. We will further supplement the manuscript with detailed experimental data and conduct a comprehensive revision to more clearly present our research findings. Your suggestions are invaluable in helping us improve the quality of the manuscript. Below, we provide point-by-point responses to each of your comments and suggestions.

1. Given that the focus of the manuscript is the enhanced interfacial contact through tailored perovskite ink achieved by programmable polymerization, I suggest reinforcing this conceptual advance with additional experimental evidence. Specifically, contact angle and surface energy

analysis should be performed to distinguish the effects of the monomer (BFDGE), the curing agent (IPDA), and the final polymer. This is fundamental to a mechanistic understanding.

Author reply: Thank you for your insightful suggestion regarding the quantitative understanding of enhanced interfacial contact achieved by stepwise polymerization strategy. As suggested, we conducted systematic contact angle measurements and calculated the surface tension of the perovskite precursor inks containing different components (control, BFDGE-only, IPDA-only, and BFDGE+IPDA).

As shown in **Figure S3** and **Table S1**, the surface tension of the precursor ink was notably reduced from 35.0 mN/m (control) to 28.5 mN/m (BFDGE+IPDA, target). In addition, it is observed that the stepwise polymerization system exhibited a more pronounced improvement in wettability compared to individual components, indicating that the formation of hydroxyl groups during the stepwise ring-opening reaction effectively reduces liquid-vapor interfacial tension, thereby optimizing the spreading and coverage of the perovskite ink on hydrophobic SAM-based HTLs. This finding provides direct experimental evidence for the critical role of our strategy in enhancing interfacial contact and demonstrates its potential for scalable fabrication of large-area PSMs.

Your input has been invaluable in helping us strengthen the rigor of our work. We have included this in the revised manuscript, and the changes are also highlighted.

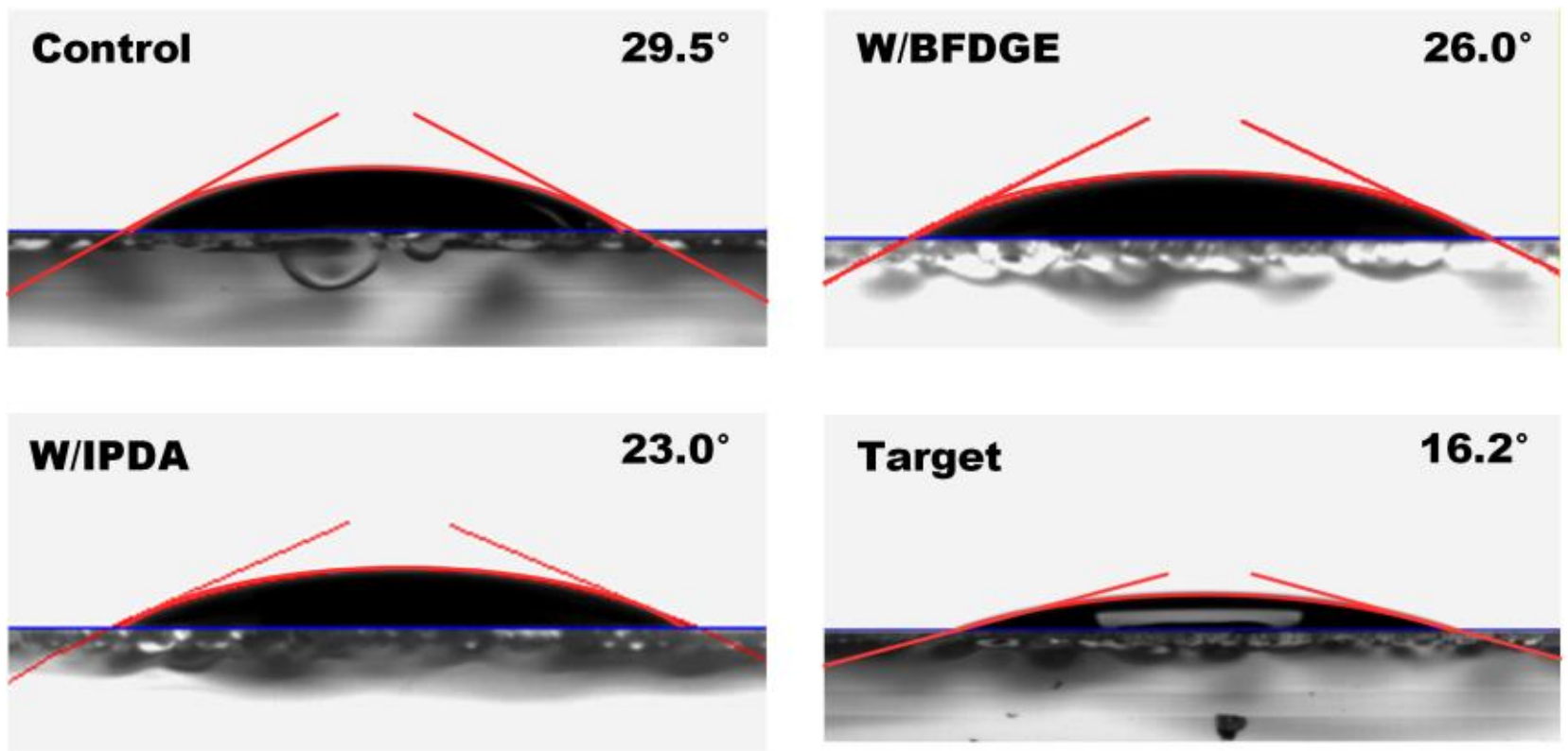

Figure S3. Contact angles of the perovskite precursor on the glass/ITO/SAM substrates.

Table S1. Contact angles and surface tension of control and perovskite precursor with BFDGE, IPDA, and BFDGE + IPDA.

	Control	W/BFDGE	W/IPDA	Target
θ (°)	29.5	26.0	23.0	16.2
γ (mN/m)	35.0	33.3	31.9	28.5

2. The authors demonstrate improved film coverage through optical photographs (e.g., on 5×5 cm^2 substrates) and provide surface-level characterizations, however, a direct investigation of the perovskite/SAMs interface is crucial to elucidate the fundamental mechanism. I suggest probing the buried interface using techniques like PL Mapping, TRPL, SEM, etc. (see, e.g., Science, 390, 638-642 (2025); Science, 391, eadz7969 (2026)) to provide direct mechanistic insight into how the programmable polymerization promotes scalable fabrication.

Author reply: Many thanks for your valuable feedback. To assess the influence of stepwise polymerization strategy on the perovskite growth at the buried interface, we peeled the perovskite films using UV-curable adhesive following the procedures illustrated in **Figure R1**. Photographs of the peeled films visually confirmed the formation of robust ITO/SAMs/perovskite interface in the target samples.

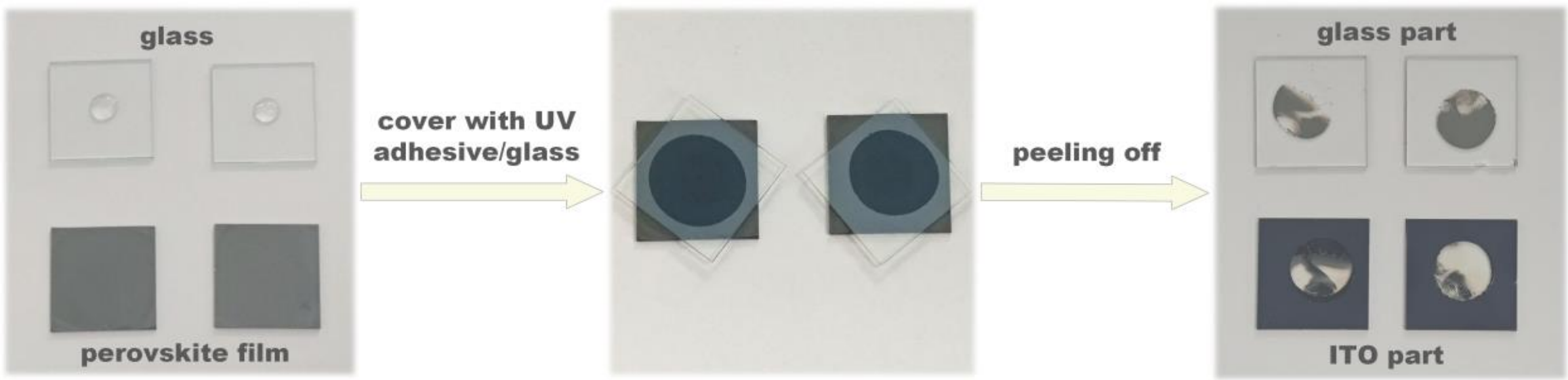

Figure R1. Schematic and photographs demonstrate the mechanical peeling test used to evaluate interfacial adhesion with a glass/ITO/SAMs/Perovskite architecture. Firstly, the perovskite film was covered with UV adhesive/glass. Following 2 minutes of curing, a tensile peeling force was applied, separating the structure into two distinct sections: an "ITO part" (including the ITO/SAMs substrates and possible perovskite remnants) and a "glass part" (consisting of the cover glass, UV adhesive, and the upper functional layers). Samples were chosen such that sufficient perovskite remained on the glass side after peeling, ensuring that the signal predominantly reflects the bottom region of perovskite layer near the buried interface.

We further imaged the bottom interface morphology with scanning electron microscopy (SEM). As illustrated in **Figure S7a**, the pristine perovskite film deposited on the hydrophobic substrate comprises numerous small grains with an average size of 309.12 nm, alongside distinct pinholes and PbI_2 fragments distributed throughout the surface. In contrast, the perovskite film subjected to the stepwise polymerization strategy, which enhances crystallization and wettability, exhibited significantly enlarged and more uniform grain sizes (average size: 551.71 nm), accompanied by pinhole-free morphology and nearly eliminated PbI_2 **(Figure S7b**) relative to the control sample.

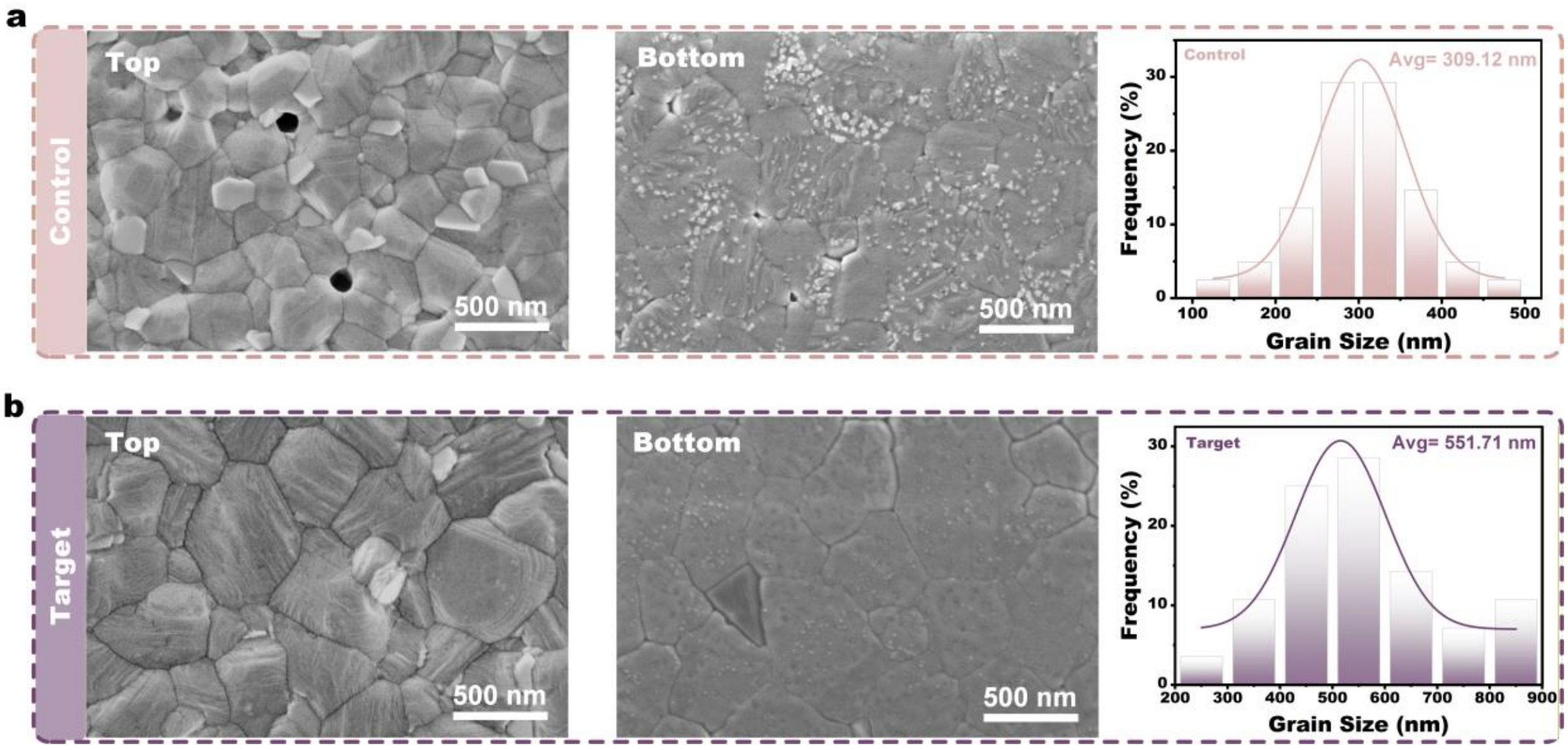

Figure S7. SEM images and the corresponding grain size distribution of the control (a), target (b) perovskite films.

Additionally, PL mapping reveals that the target sample exhibits subsequently enhanced and more uniform emission intensity compared to the control, which shows distinct weakly emissive regions, indicating improved perovskite growth on the hydrophobic SAMs' surface (**Figures 4d, e**). TRPL measurements show that the target achieves a carrier lifetime of 1.9 μs, which is 1.58 times longer than that of the control, confirming reduced defect density at the buried interface (**Figure 4c**).

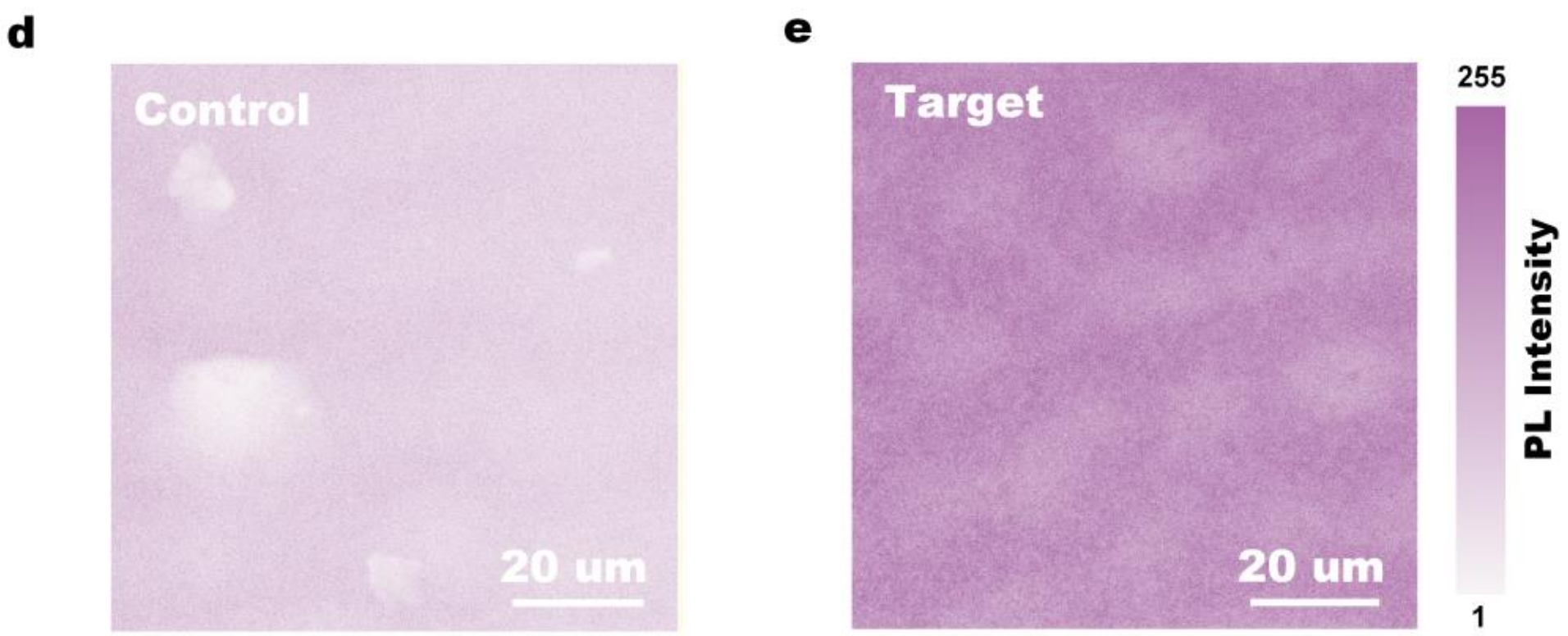

Figures 4d and 4e. PL mapping patterns collected from the buried interface for the control (d) and target (e) perovskite films.

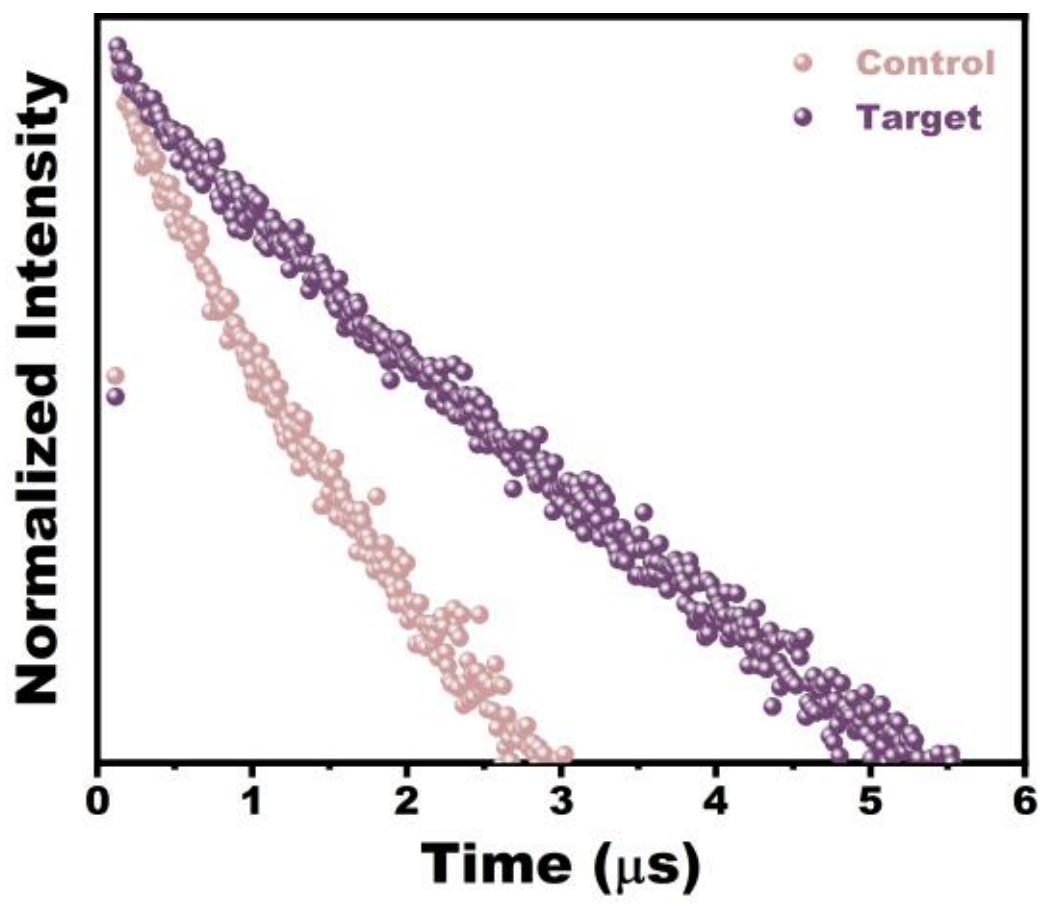

Figure 4c. TRPL decay profiles measured from perovskite bottom side.

We hope this additional testing adequately addresses your concerns and provides a more comprehensive evaluation of the stepwise polymerization strategy in promoting scalable fabrication.We have included this in the revised manuscript, and the changes are also highlighted.

3. The manuscript claims that "the resulting polymer with hydrophobic nature can serve as a bulk encapsulation medium (BEM), which will enhance the moisture resistance and overall stability of the devices." However, the sole MPPT tracking test over 1000 hours primarily assesses operational stability under continuous illumination and electrical bias, which is insufficient to substantiate specific claims regarding moisture resistance or the encapsulation function. We suggest that the authors reconsider this part of their statement, or provide targeted evidence (e.g., damp-heat testing) for the specific moisture-resistance and encapsulation properties.

Author reply: Thank you for your constructive suggestion. We fully agree that while MPPT tracking evaluates operational stability, it may not fully represent the moisture-resistance or encapsulation capabilities of the device. In response, we have conducted a rigorous stability assessment following the ISOS-T-3 protocol (which includes damp-heat and thermal cycling) on the encapsulated modules.

As depicted in **Figure R2**, the devices were subjected to simultaneous fluctuations of temperature (ranging from -40 °C to +85 °C) and relative humidity (10-80%). As expected, the target module retained 94.3% of its initial PCE after 200 cycles, notably outperforming the control sample, which retained only 77.3% of its PCE (**Figure 6c**). This superior performance substantiates our claim that the formation of the in-situ crosslinked polymer scaffold can serve as an effective bulk encapsulation medium (BEM). BEM not only passivates the grain

boundaries to mitigate ion migration but also acts as a robust physical barrier, significantly increasing the diffusion path for moisture and oxygen molecules. We have included this in the revised manuscript, and the changes are also highlighted.

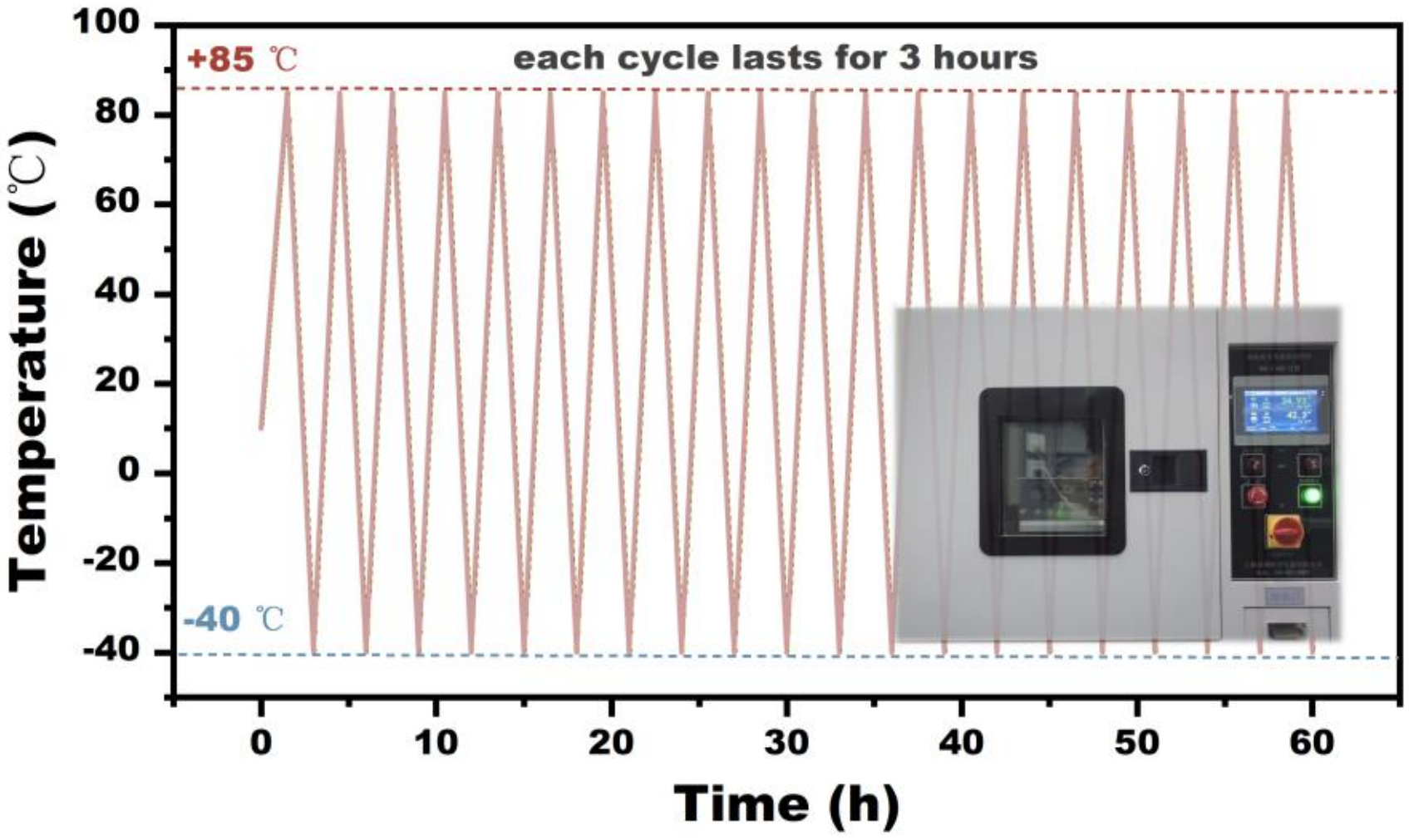

Figure R2. Temperature profile over time for thermal cycling (each cycle lasting ~3 hours).

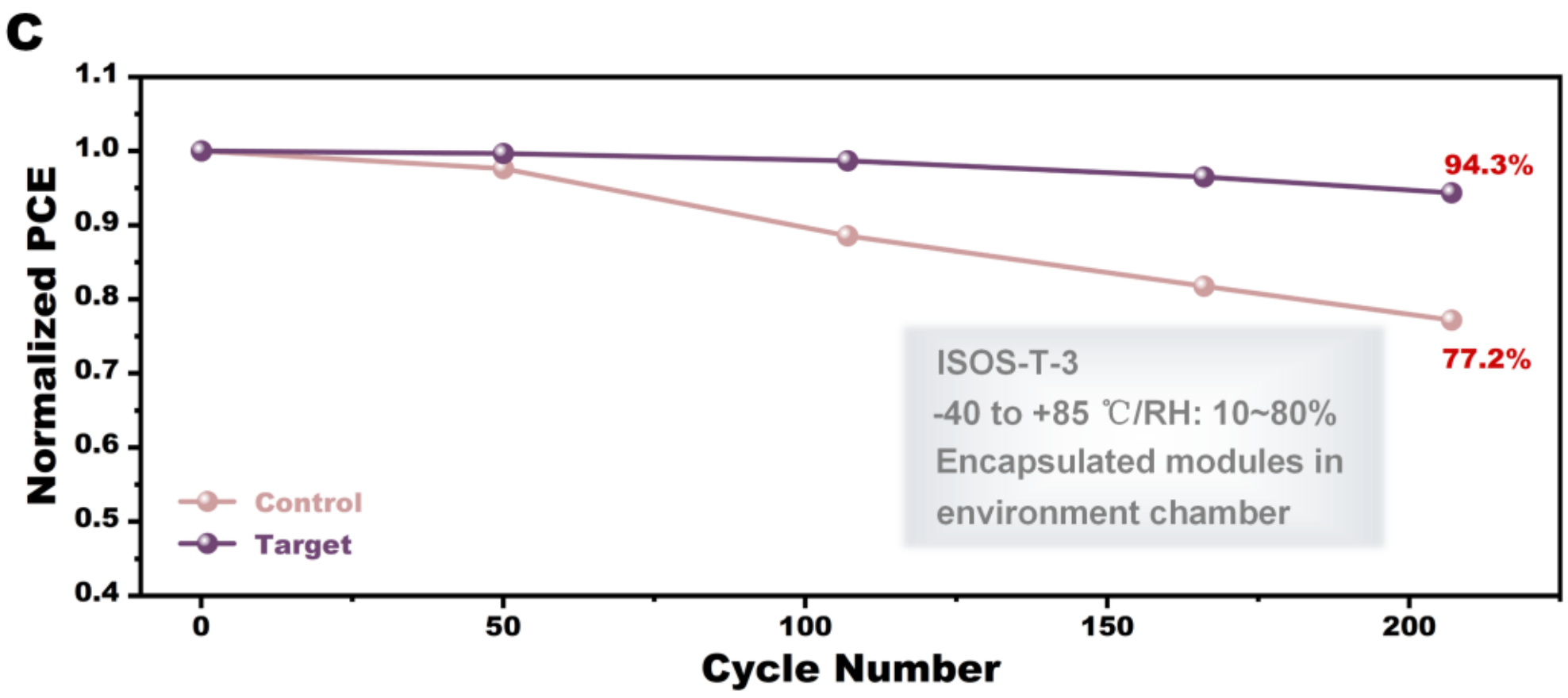

Figure 6c. Thermal cycling stability of encapsulated modules aged between -40 °C to +85 °C (ISOS-T-3).

4. The shifts in XPS peaks (Pb 4f, N 1s, I 3d) to lower binding energy in Figures 1C-E provide compelling evidence for surface chemical passivation by the BEM. However, as XPS is a surface-sensitive technique, it cannot assess the bulk or depth distribution of the BEM. Therefore, time of light secondary ion mass spectroscopy (TOF-SIMS) measurements are recommended to monitor the spatial distribution of BEM, which is beneficial for investigating the detailed influence of the additive on the perovskite film.

Author reply: We thank the reviewer for the helpful feedback. To complement the surface-sensitive XPS analysis, we have performed time-of-flight secondary ion mass spectrometry (ToF-SIMS) to investigate the spatial and depth distribution of the BEM within the perovskite

film. In the ToF-SIMS analysis, PbI^- was selected as the representative signal of the perovskite phase, while O^- was used to represent the BEM. As shown in **Figure S27**, no significant O^- signal was detected in the control sample; however, the BEM-related signal (O^-) was detected throughout the entire thickness of the perovskite film. This confirms that BEM is uniformly distributed within the bulk and is likely localized at the grain boundaries as a 3D scaffold. Do hope these clarifications and additional experimental results address the reviewer's concerns. We have included this in the revised manuscript, and the changes are also highlighted.

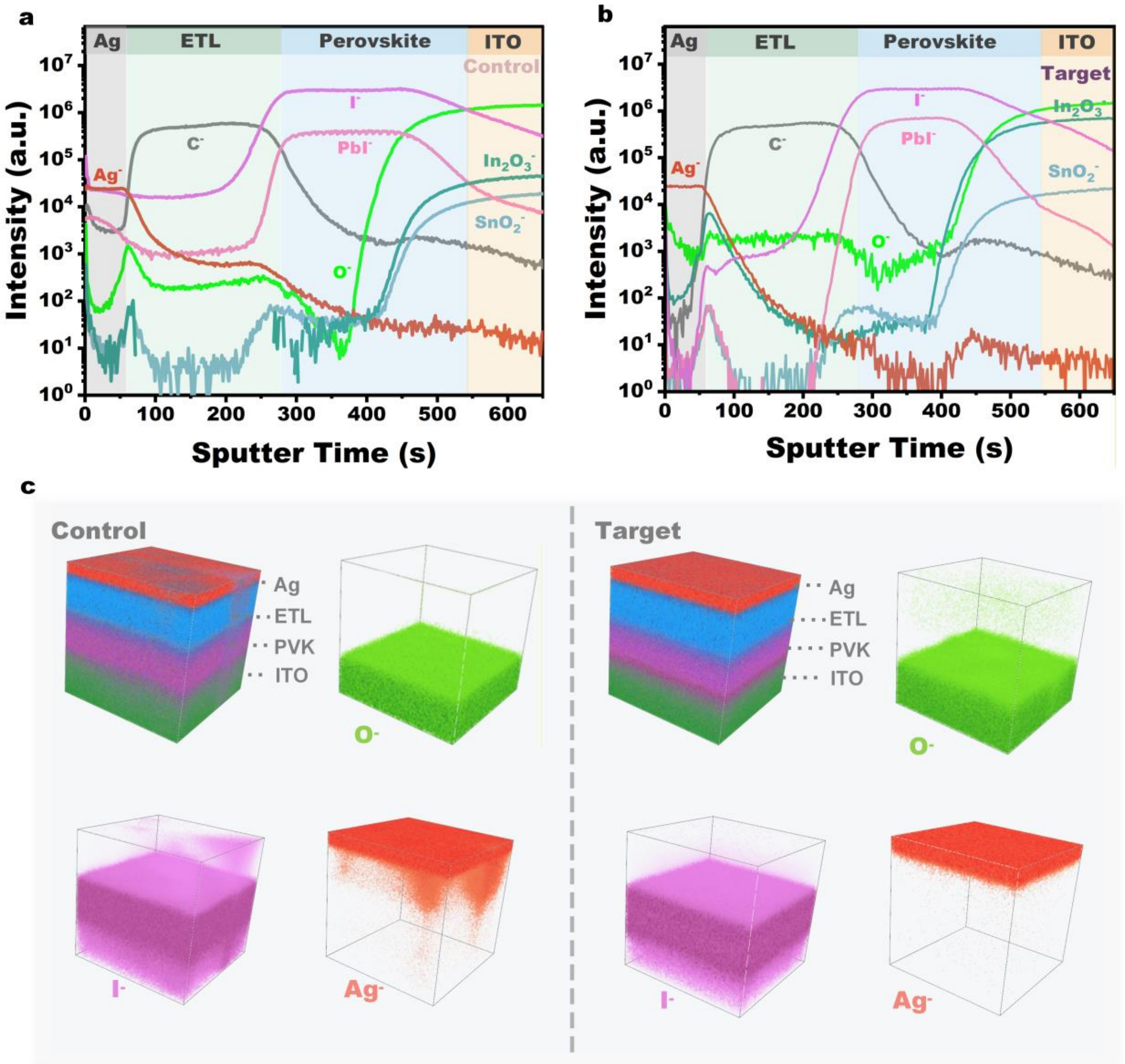

Figure S27. Time-of-light secondary-ion mass spectrometry (TOF-SIMS) depth profiles after 100h MPP tracking. (a) Control device. (b) Target device. (c) Corresponding TOF-SIMS 3D tomography results for control and target devices.

5. It would be beneficial if the authors could provide more details on the fabrication process of

perovskite solar sub-modules to enhance reproducibility for other research groups.

Author reply: We appreciate the reviewer's insightful comment. In response, we have added detailed information regarding the sub-modules' fabrication process in the experimental section of the revised supporting information. The content is as follows:

Sub-modules fabrication process

The 1.2 M perovskite precursor solution with the nominal composition of $Cs_{0.05}(FA_{0.98}MA_{0.02})_{0.95}(I_{0.98}Br_{0.02})_3$ was prepared by dissolving FAI, MABr, CsI, PbI_2, and $PbBr_2$ in a mixed solvent of DMF and NMP (6:1 volume ratio). To accommodate the slot-die coating process conducted under ambient conditions (T: 23 °C, RH: 20%), an additional 10% MACl, 3% $PbCl_2$, and 5% PbI_2 were introduced into the precursor to enhance the crystallization quality of the perovskite film.

For the 300 × 300 mm^2 ITO substrates, the P1, P2, and P3 scribing were conducted using the same femtosecond laser system and power/speed parameters described for the mini-modules.The specific process for the sub-module includes:

Transport layer: A NiO_x hole transport layer doped with 1% Mg was deposited by magnetron sputtering to a thickness of 8 nm. Subsequently, a 4PADCB layer was coated by slot-die coating using an ethanol solution with a concentration of 0.5 mg/mL, at a coating speed of 100 mm/s and a gap height of 80 μm. The film was directly annealed at 100°C for 10 min without vacuum flash.

Perovskite layer: The perovskite layer was then deposited by slot-die coating at a coating speed of 80 mm/s with a gap height of 100 μm. Immediately after coating, the substrate was subjected to vacuum flash evaporation at a minimum pressure of 10 Pa for 65 s. Pre-annealing was carried out at 70 °C for 3 min, followed by a final annealing at 100 °C for 30 min.

6. Despite the impressive device performance achieved by the authors, I would like to raise a question regarding the significant performance gap between the rigid and flexible devices. Could the authors provide further explanation for this discrepancy?

Author reply: Thank you for your thoughtful question regarding the performance gap between rigid and flexible devices. We believe this discrepancy primarily arises from two factors: poorer crystallization and optical losses.

Firstly, the rougher surface and lower thermal tolerance of flexible plastic substrates lead to uncontrolled nucleation and inferior perovskite crystallization compared to rigid glass

substrates, resulting in higher defect density and reduced device performance.

Secondly, there exists an inherent trade-off between high transparency and good conductivity in flexible substrates. As shown in **Figure R3**, we measured the transmittance of glass/ITO and PEN/ITO substrates. The flexible PEN/ITO substrate exhibits significantly lower transmittance across the visible spectrum compared to rigid glass/ITO, leading to reduced light harvesting and consequently lower photocurrent in flexible devices.

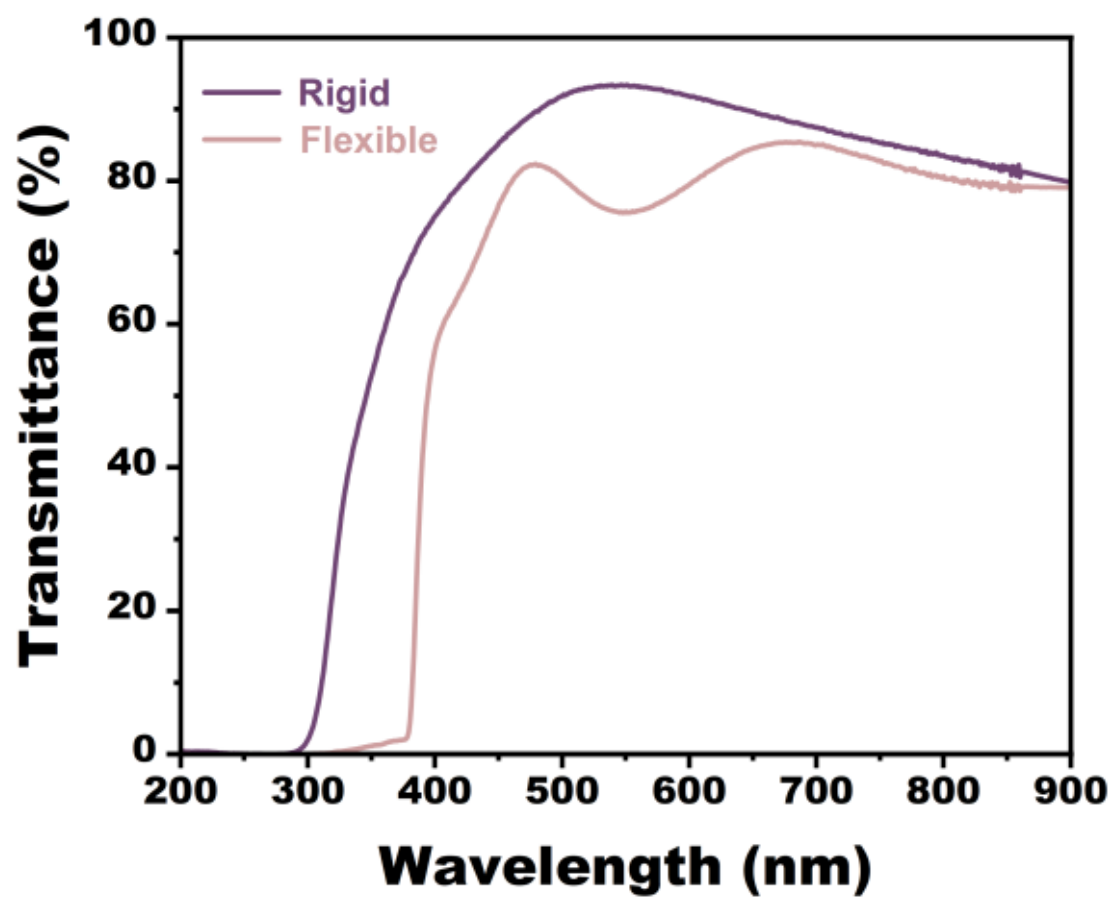

Figure R3. Transmittance spectra of rigid glass/ITO and flexible PEN/ITO substrates.

Referee: 2

Comments:

This manuscript presents a programmable polymerization strategy using BFDGE and IPDA to tailor perovskite ink for scalable flexible perovskite solar cells (f-PSCs). The authors report a good large-area module performance. While the work is technically sound, several critical concerns preclude its publication in its current form. However, the novelty of the present work is limited which does not meet the publication requirement of this journal.

Author reply: We thank you for the thoughtful comments and the time you have taken to review our manuscript. Regarding the concern about novelty, we have carefully reflected on the reviewer's feedback and have now further articulated the unique contributions of our strategy in the revised manuscript.

We would like to respectfully clarify the unique aspects of our study that distinguish it from previously reported work regarding the cross-linkable monomers or polymerizable additives. While prior works have indeed demonstrated the use of polymerizable materials to enhance

perovskite film quality, our study introduces a conceptually distinct approach, stepwise polymerization, that dynamically solves the universal "substrate-dependence" bottleneck in scalable perovskite fabrication. To further substantiate the unique scientific and technological impact of our work, we have performed comprehensive new experiments and refined the manuscript as follows:

1. Deepened Mechanistic Insights at the Buried Interface: We have measured the surface tension of the tailored inks and performed high-resolution characterizations of the buried interfaces. The results confirm that our strategy not only reduces the ink's surface tension but also promotes the formation of a highly uniform, void-free buried interface. Furthermore, newly added data on carrier recombination dynamics (e.g., TRPL and PL mapping) further demonstrate that the improved crystallinity and the resultant scaffold effectively passivate interfacial defects and suppress nonradiative recombination losses.

2. Demonstrated Universal Applicability: To highlight the practical significance, we have extended our study to a wide range of mainstream commercial SAMs (e.g., Ph-PACz, 4PABCz, MeO-4PACz, etc.). The results show a consistent and notable improvement in ink spreading and wetting across all tested hydrophobic surfaces, establishing our strategy as a substrate-agnostic platform for scalable PSCs.

3. Validation of Industrial-Level Stability: We have supplemented the stability data with rigorous testing following the ISOS-T-3 protocol (simultaneous damp-heat and thermal cycling) and UV aging tests. The encapsulated modules maintained over 94% of their initial efficiency under these harsh conditions, underscoring the role of the crosslinked polymer scaffold as a robust physical barrier and mechanical buffer.

We believe that these contributions collectively represent a significant and robust advancement in the field of perovskite solar cells, particularly in terms of achieving both high efficiency and stability in flexible devices. We hope the revised manuscript more clearly conveys the potential impact of our work on the community, and we welcome any further suggestions on how to further strengthen it.

Our point-by-point responses to the comments/suggestions made by Reviewer 2 appear below.

1. The concept of using cross-linkable monomers or polymerizable additives to improve perovskite film quality, stability, and scalability has been extensively reported in recent literature (e.g., Refs. 19-21, 29, 41). The authors' approach, while well-executed, does not introduce a fundamentally new mechanism or material paradigm. The use of epoxy-amine chemistry, though effective, has been applied in other contexts. The incremental advance does

not meet the threshold of transformative or groundbreaking research required for publication in a high-impact journal.

Author reply: We appreciate your insightful perspective regarding the existing literature on cross-linkable additives. While we fully acknowledge the significant contributions of prior works (Refs. 19-21, 29, 41) in enhancing film quality and stability, we respectfully emphasize that our work addresses a distinct and critical challenge in the field of scalable fabrication that remains unresolved by conventional static additive engineering.

The core innovation of our work lies not merely in the use of cross-linkable additives, but in kinetic regulation enabled by stepwise polymerization, which dynamically controls the ink-substrate interface during the critical early stages of film formation. The uniqueness contributions of our strategy are substantiated through the following three dimensions:

1. A Distinct Mechanism, Stepwise Polymerization for Interfacial Engagement: Most reported cross-linkable monomers or polymerizable additives are designed as passivators for crystallization. Our approach leverages the intermediate stages of the epoxy-amine reaction to regulate the ink/substrate engagement. As shown in **Figure R3,** the intermediate oligomers formed during the stepwise reaction temporarily reduced the ink's surface tension, creating a critical kinetic window that enables complete spreading and conformal contact on hydrophobic SAM-modified substrates before the formation of a rigid polymer network. Crucially, this regulation is kinetic in nature, as it leverages the reaction pathway itself to control wetting behavior, rather than relying solely on static chemical modification. This addresses the long-standing reliability bottleneck in the scalable fabrication of p-i-n devices, which is typically plagued by de-wetting and poor interfacial contact. Experimental validation at the buried interface (new **Figure S7**) further confirms that this strategy yields a compact, void-free morphology.

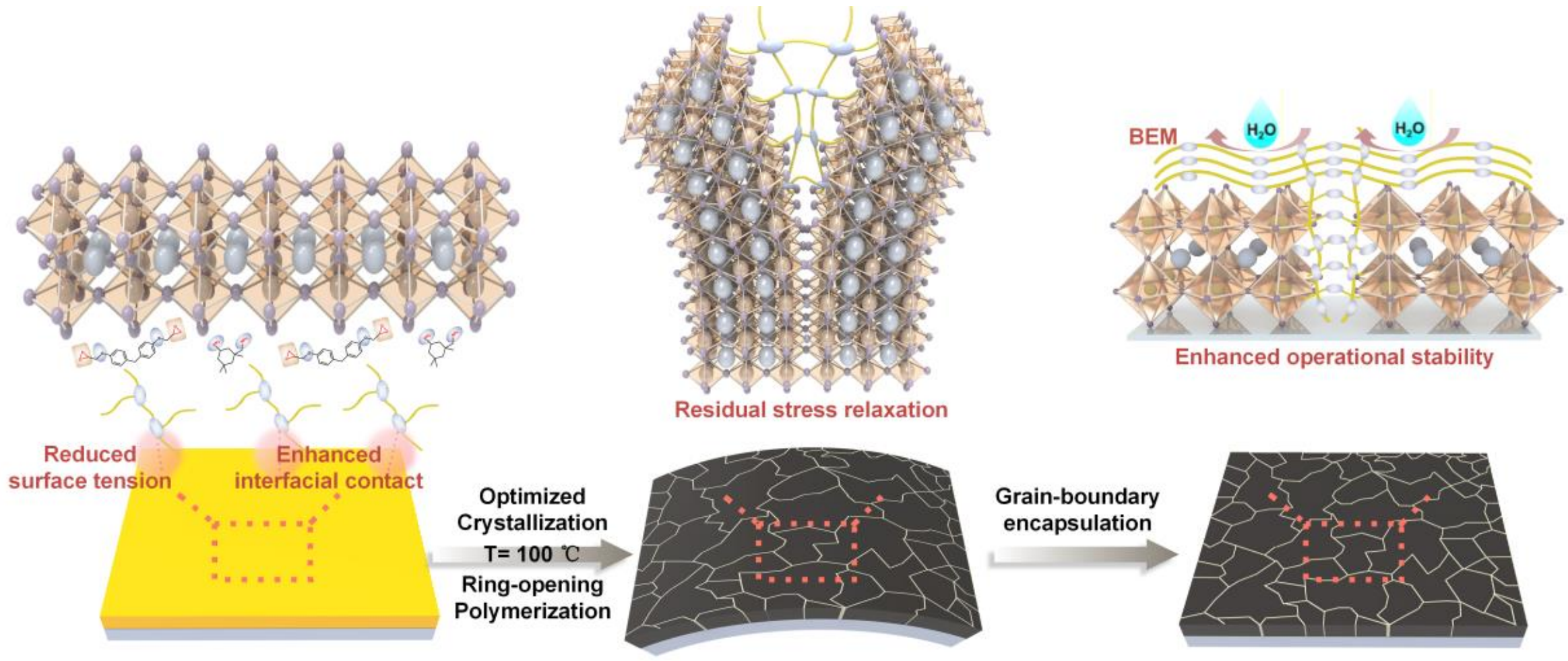

Figure R4. Orchestrating the interface and crystallization for stable perovskite solar cells via a programmable stepwise polymerization strategy.

2. Substrate-Agnostic Versatility: To address the concern of "incremental advance", we have supplemented the manuscript with a universal applicability test (**Figure S4**). We demonstrate that our tailored ink consistently achieves improved wetting across multiple representative commercial SAMs (e.g., Ph-PACz, 4PABCz, MeO-4PACz, etc.). This proves that our strategy is not a case-specific fix but rather a robust and versatile engineering platform for resolving the universal "substrate-dependence" issue in inverted-structured PSCs.

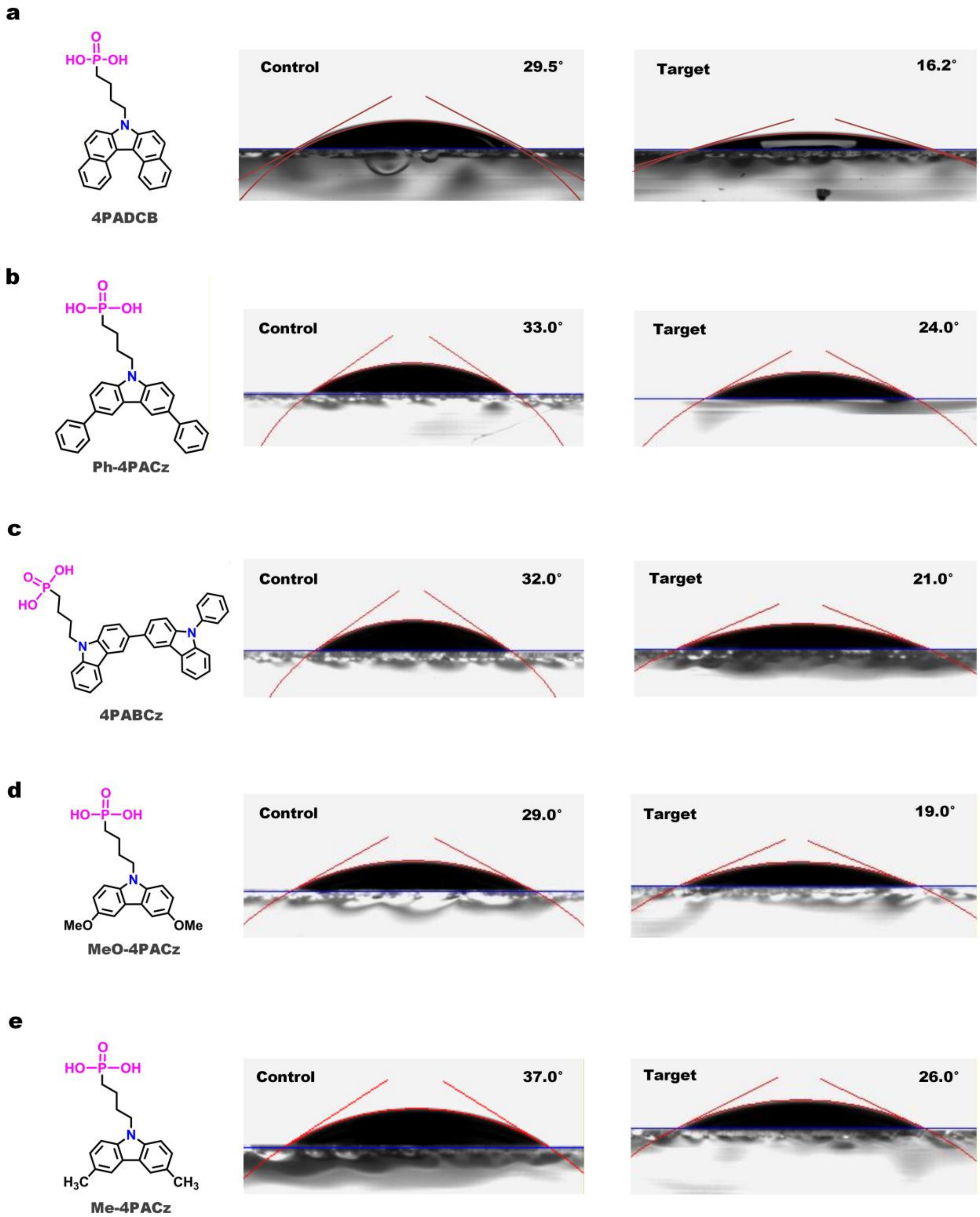

Figure S4. Comparison of contact angles for control and target perovskite inks on diverse SAM-based hole transport layers.

3. Scalability Verification: The practical relevance of this strategy is substantiated by the reliable transition from lab-scale cells to large-area modules. As summarized in **Figure R5**, we have successfully fabricated 10.24 cm^2 mini-modules and achieved a notable efficiency of

22.60% for 655.2 cm^2 sub-modules, which to the best of our knowledge represents one of the highest reported performances for inverted perovskite devices of this class size. This scalability is a crucial step toward the commercial application of SAM-based inverted PSCs, addressing a significant challenge in the field. These results underscore the practical relevance and the transformative impact of our work.

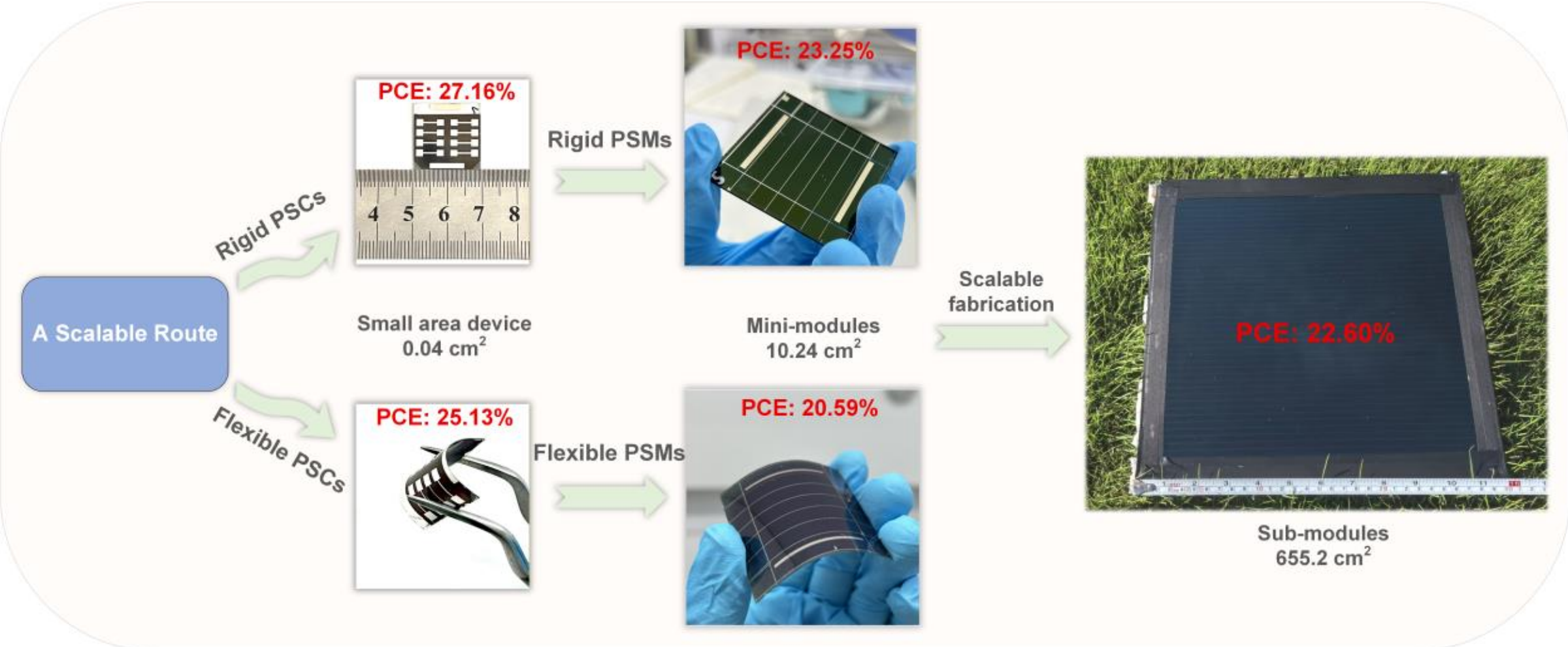

Figure R5. Presentation of the proposed stepwise polymerization strategy is a scalable route.

In summary, we believe that repurposing epoxy-amine chemistry as a stepwise regulation system to manipulate the ink-substrate interface represents a significant conceptual leap beyond existing additive-based approaches. We hope this addresses your concerns, and we are open to further suggestions for additional experiments. All corresponding changes have been incorporated into the revised manuscript and are clearly highlighted for clarity.

2. The proposed "programmable" nature of the polymerization is not clearly demonstrated or quantified. The term "programmable" suggests tunable or sequential control, but the presented data only shows a standard stepwise epoxy-amine reaction synchronized with annealing.

Author reply: We thank the reviewer for this insightful correction and agree that the term "programmable" may be inappropriate, as it implies a degree of external control. Accordingly, we have replaced it with "stepwise" throughout the revised manuscript to more accurately describe the sequential kinetic evolution of the epoxy-amine reaction-from the formation of intermediate oligomers to final cross-linking. We believe this terminology better reflects intrinsic nature of the polymerization process and more precisely conveys practical advantages of our strategy.

3. The distribution and role of the BEM (bulk encapsulation medium) at grain boundaries, while shown via EDX and HRTEM, lacks direct evidence of its dynamic interaction with perovskite crystallization in real time.

Author reply: Thanks for your insightful comment. We agree that while HRTEM and EDX provide valuable spatial evidence of BEM localization at grain boundaries, capturing dynamic interaction during crystallization is crucial for a complete mechanistic understanding.

In the original manuscript, we have employed in-situ photoluminescence (PL) and in-situ UV-Vis spectroscopy to monitor the dynamic crystallization process (**Figures 2a-f**). These optical characterizations provided initial evidence that the stepwise polymerization strategy effectively modulates the crystallization kinetics and extends the phase transition window, ensuring a more controlled film formation process.

To directly monitor this dynamic process and provide real-time structural evidence, we have performed in-situ X-ray diffraction (XRD) measurements during thermal annealing to directly monitor the crystallization evolution of perovskite films. As shown in the revised **Figures 2g-i**, the control film exhibits a distinct diffraction peak at approximately 6.55°, corresponding to a solvent-containing intermediate phase (e.g., DMSO-PbI_2 complex), consistent with previous reports intermediate-mediated crystallization pathways (*Nature Materials* 13, 897-903 (2014); *Advanced Science* 10, 2300879 (2023)). This intermediate phase persists for an extended duration, and the characteristic (100) perovskite peak at ~14° emerges slowly and with relatively weak intensity. In contrast, the target film shows a markedly reduced intermediate phase signal, accompanied by a more direct and robust emergence of the (100) perovskite peak. These observations indicate that the ring-opening reaction between BFDGE and IPDA actively modulates the crystallization pathway by suppressing the formation of stable intermediate phases.

By integrating the original in-situ optical analysis (PL/UV-Vis) with the newly added in-situ structural evidence (XRD), we have established a complete real-time characterization chain. These results collectively demonstrate that the stepwise polymerization strategy dynamically modulates the crystallization pathway. We have also incorporated these results into the revised manuscript, with the changes highlighted for clarity. We thank you again for guiding us toward this more complete analysis.

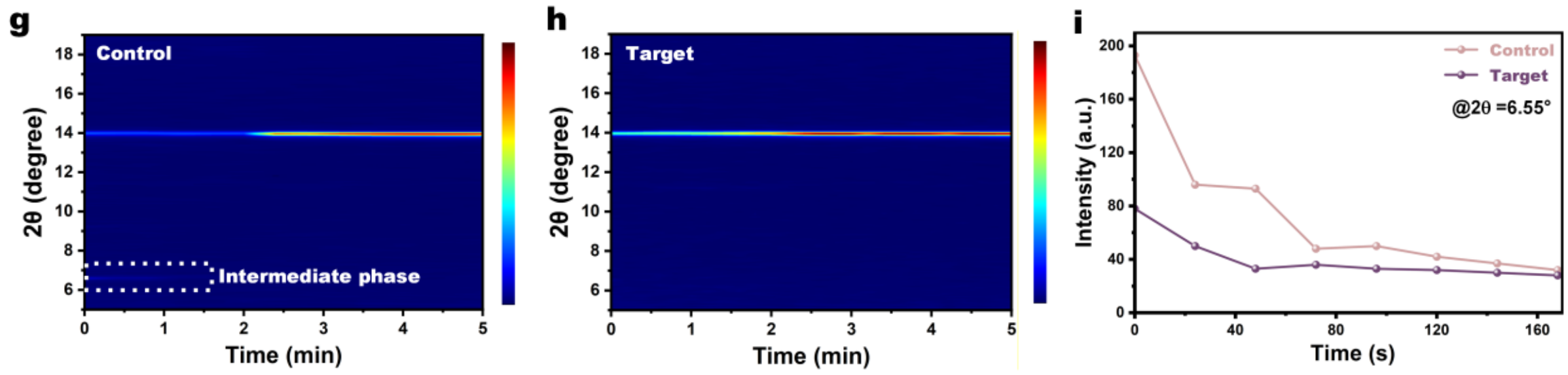

Figures 2g-i. In situ XRD spectra of (g) control and (h) target perovskite films during the thermal annealing. (i) The extracted XRD peak intensity at 2θ=6.55° of the corresponding films during the annealing procedure.

4. Claims regarding strain relaxation and ion migration suppression are supported indirectly (e.g., GIXRD, ToF-SIMS), but a more direct, in-situ or operando study linking BEM distribution to mechanical and ionic behavior is missing.

Author reply: We sincerely thank you for this insightful suggestion. We agree that establishing a more direct correlation between the BEM distribution and the resulting mechanical/ionic behavior is essential for a deeper understanding of the stabilization mechanism. In response, we have supplemented the manuscript with additional characterizations, including KPFM and peak force quantitative nanomechanical AFM (PFQNM-AFM) for the surface potential distribution and Young's modulus of perovskite films, to complement the existing activation energy (E_a) analysis:

1. Ion Migration Barrier: In the original manuscript, **Figure 5f** shows that the ion migration activation energy (E_a) increased from 0.16 (control) to 0.24 (target) eV. This elevated energy barrier provides kinetic evidence that the BEM network at grain boundaries effectively obstructs ion migration.

2. Surface Potential Mapping: We have re-conducted high-resolution KPFM measurements (**Figure S10**) to quantify the effect of BEM on the surface potential distribution of perovskite films. The results demonstrate that the control perovskite film has a substantial potential difference between the grain boundaries and the grains, which is notably reduced in the BEM-containing perovskite films. Normally, the presence of charged defects such as undercoordinated Pb^{2+} at grain boundaries would hinder intergranular charge transfer due to electrostatic forces and charge localization, leading to charge accumulation and recombination at the interface. For the perovskite film with BEM distributed along grain boundaries, the smaller surface potential difference implies reduced defect density and a weaker energy barrier between grains, which will facilitate more efficient charge transport across grain boundaries.

3. Mechanical Reinforcement: To further study the mechanical properties of the modified

films, peak force quantitative nanomechanical AFM (PFQNM-AFM) was conducted to characterize the Young's modulus (**Figure S13**). As expected, the target film displayed a Young's modulus of 2.11 GPa, which was notably smaller than that of control (6.77 GPa). This proves that the cross-linked BEM serves as a mechanical "scaffold" at the grain boundaries to buffer strain and relax stress, which in consistent with GIXRD observations.

In summary, these results establish a direct correlation between the spatial distribution of BEM and the suppression of ion migration and strain relaxation. We have incorporated these findings in the revised manuscript, and the corresponding changes are also highlighted for clarity. We thank you again for guiding us toward this more comprehensive analysis.

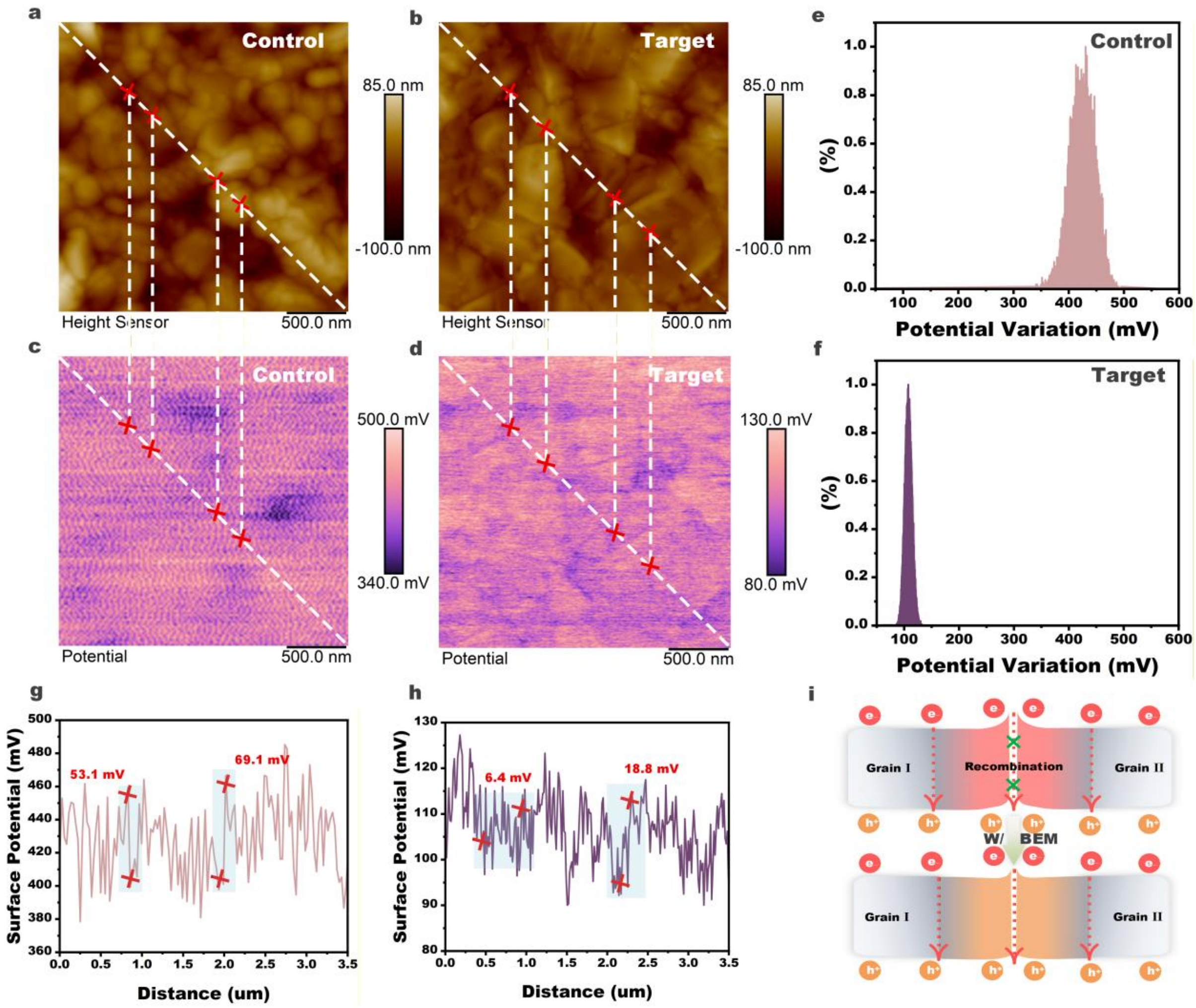

Figure S10. Surface morphology and potential distribution of perovskite films. (a, b) AFM images of control (RMS= 23.6 nm) and target (RMS= 14.3 nm) perovskite films. (c-f) KPFM images and the surface potential variation of perovskite films. (g, h) The corresponding surface potential variation between grains and grain boundaries along the diagonal line for control and BEM-modified perovskite films. (i) Schematic diagram of the intergranular carrier transfer process.

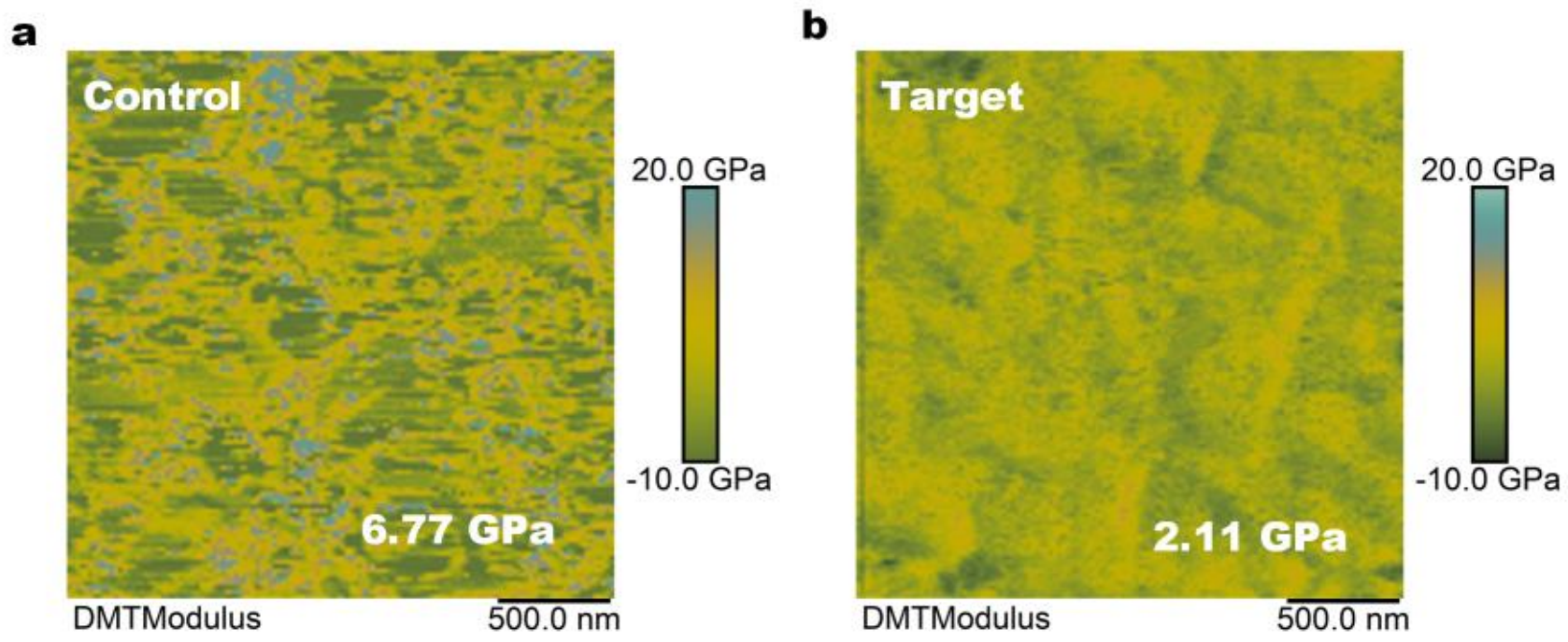

Figure S13. Young's modules of perovskite films.

5. The study lacks a thorough analysis of potential drawbacks, such as the effect of BEM on charge transport across grain boundaries, long-term chemical stability of the polymer under UV exposure, or possible interfacial degradation during thermal cycling.

Author reply: We appreciate your constructive suggestion to evaluate the potential drawbacks of the stepwise polymerization strategy. We have performed additional rigorous aging tests according to international standards to address concerns regarding charge transport, UV stability and thermal-mechanical reliability.

Charge Transport Across Grain Boundaries: To assess whether the presence of BEM at grain boundaries impedes charge transport, we have re-conducted high-resolution KPFM measurements (**Figure S10**) to quantify the effect of BEM on the surface potential distribution of perovskite films. The results demonstrate that the control perovskite film has a substantial potential difference between the grain boundaries and the grains. In contrast, the BEM-modified film displays a markedly reduced potential variation, implying effective defect passivation and a weakened energy barrier between grains. This homogenization directly correlates with the presence of BEM at grain boundaries and facilitates more efficient charge transport.

Moreover, these KPFM observations are fully consistent with our previously reported time-resolved photoluminescence (TRPL) and space-charge-limited current (SCLC) measurements (**Figures 4c** and **4i** in the revised manuscript). The BEM-modified films exhibit prolonged carrier lifetimes and significantly reduced trap densities compared to control films, further confirming that the cross-linked polymer scaffold not only avoids impeding charge transport but actively enhances it through effective chemical passivation of defect states at grain boundaries. Collectively, these results demonstrate that the stepwise polymerization strategy improves rather than compromises charge transport properties.

Thermal Cycling Stability: To investigate potential interfacial degradation during thermal stress, we subjected the encapsulated modules to a combined damp heat and thermal cycling test (following ISOS-T-3 protocol). As shown in **Figure R2** and **Figure 6c**, the BEM-containing module retained 94.3% of its initial PCE after 200 cycles, notably outperforming the control module, which retained only 57% of its PCE. This confirms that the cross-linked BEM scaffold provides a robust mechanical buffer and a physical barrier against moisture and thermal stress, rather than causing degradation.

UV Stability: To evaluate long-term chemical stability under UV exposure, we subjected encapsulated devices to continuous UV irradiation (365 nm, 180 μW/cm^2) for 630 hours. As shown in new **Figure 5h**, the encapsulated devices treated with BEM retained an impressive 96.0% of their initial efficiency after 630 hours irradiation, while the control samples plummeted to 67.3%. This demonstrates that the encapsulated polymer network effectively suppresses UV-induced perovskite decomposition and is chemically stable under harsh radiation.

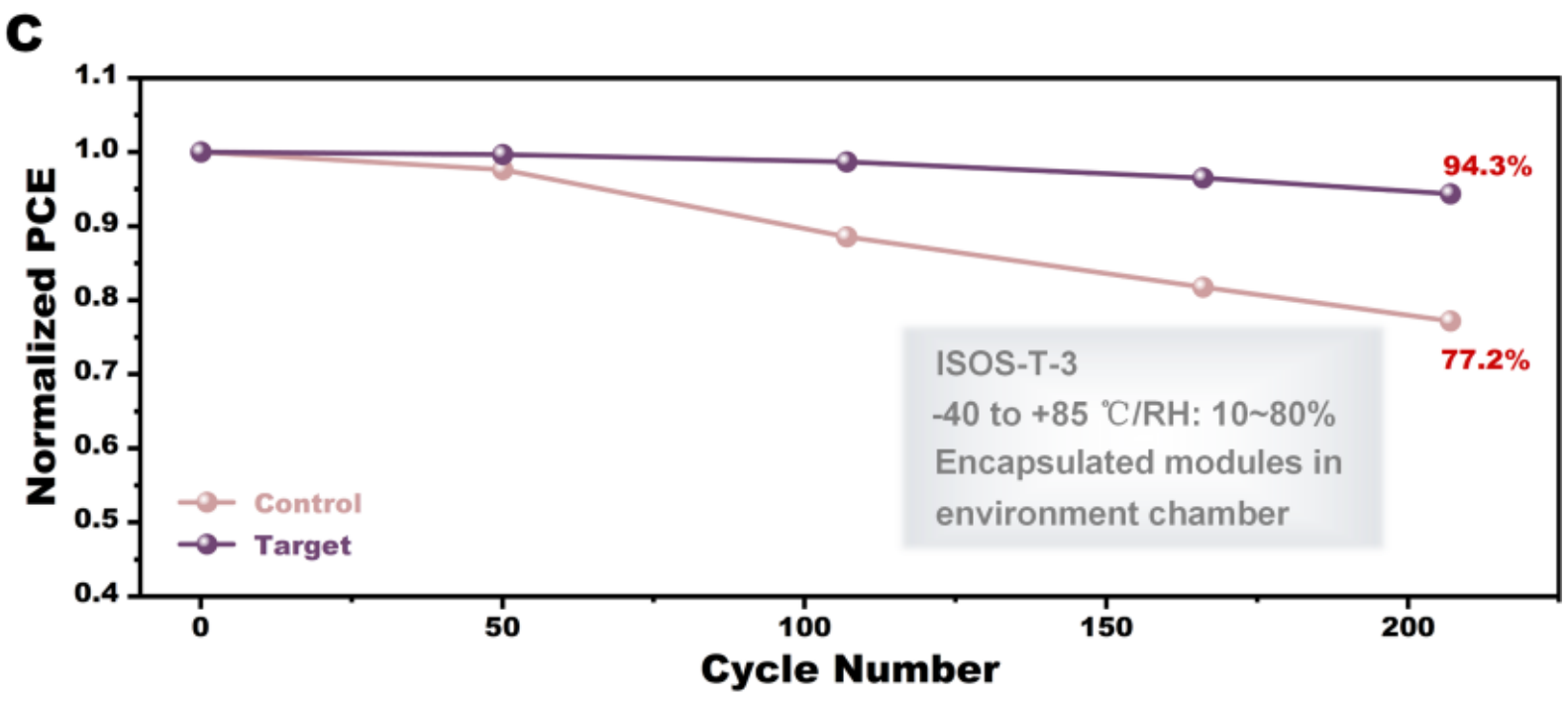

Figure 6c. Thermal cycling stability of encapsulated modules aged between -40 °C to +85 °C (ISOS-T-3).

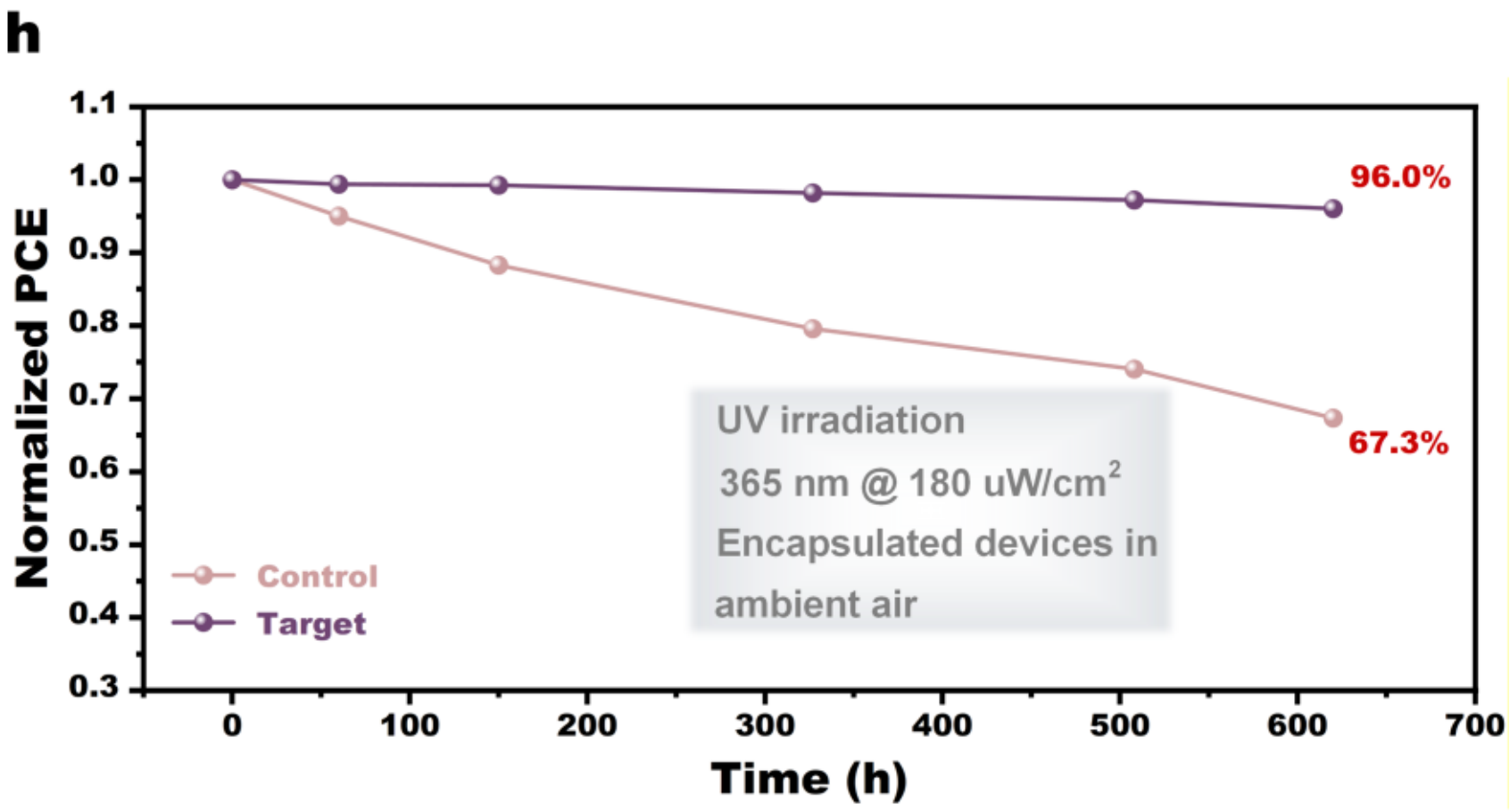

Figure 5h. UV light irradiation stability measurements of encapsulated PSCs under 365 nm UV light (180 uW/cm^2) in air from the glass side.

These results collectively confirm that the stepwise polymerization strategy not only avoids

introducing performance drawbacks but actively enhances device reliability under multiple stress conditions. We have also incorporated these results into the revised manuscript.

6. The use of "record-breaking" and "new benchmark" for the 655.2 cm^2 module is not sufficiently contextualized within the broader landscape of large-area perovskite modules, especially considering recent reports of similar or higher efficiencies on comparable areas.

Author reply: We sincerely appreciate your insightful observation and agree that the rapid progress in the perovskite field is encouraging, and it is essential to contextualize our achievements within the current state-of-the-art landscape. To address this, we have carefully refined our claims in the revised manuscript to provide a more accurate and contextualized description of our large-area module achievements, based on the following two considerations:

1. Structural Distinction (p-i-n vs. n-i-p): While recent high-profile reports have demonstrated impressive efficiencies on large-area modules (e.g., *Nature* 634, 1091-1095, (2024)), the majority of these record-high values are based on n-i-p (formal) structures. In contrast, the development of p-i-n (inverted) structured modules, which offer distinct advantages in terms of operational stability and compatibility (e.g., for tandems), has historically lagged behind. Our work specifically addresses the scalability challenges inherent to the p-i-n architecture, which remains underexplored for large-area fabrication.

2. Standardized Size Classification: According to established conventions in the photovoltaic community, devices are categorized by area as follows: mini-modules (<200 cm^2), sub-modules (200-800 cm^2), and large area modules (>800 cm^2). While recent breakthroughs have achieved meter-scale modules (e.g., *Nature* 648, 91-96, (2025)). Our 655.2 cm^2 device falls within the sub-module category, and to the best of our knowledge, its performance ranks among the highest reported to data for inverted perovskite devices of this size class.

Accordingly, we have revised the manuscript to state that our results represent a notable advancement for inverted perovskite sub-modules, rather than making an unqualified claim. We have also included a comparative summary in **Figure 6f** and **Table S10** to transparently position our work within the broader literature. We thank the reviewer again for helping us improve the precision and humility of our presentation, and we remain open to further suggestions. We appreciate you again for reviewing our manuscript.

Referee: 3

Comments:

In this manuscript, Zhang et al. report a programmable polymerization strategy that precisely controls the wettability of perovskite precursor inks on hydrophobic self-assembled monolayers (SAMs). This enables reliable, large-area deposition of high-quality perovskite films while significantly reducing substrate dependence. The strategy not only enhances the performance and operational stability of small-area devices, but also facilitates remarkable progress in scalable fabrication. The reported power conversion efficiencies are at the forefront of the field: 27.16% (certified 26.84%) for small-area devices and 22.60% (certified 21.07%) for 655.2 cm^2 submodules. This work offers valuable fundamental mechanistic insights alongside compelling technological advances. I recommend its publication in Nature Communications after the following points are addressed:

Author reply: Thank you for your positive feedback and recognition of our work. We are pleased that you appreciate our innovative stepwise polymerization strategy to achieve precise crystallization control and record-breaking efficiencies in large-area sub-modules. We will carefully consider and address each of the revision points you have outlined to further improve our manuscript.

1. The authors discuss the impact of programmable polymerization on the surface morphology of perovskite films. To further support the claim of reliable ink deposition on hydrophobic SAM-based substrates, I recommend providing additional cross-sectional SEM images of the buried interface under various treatment conditions. This would offer more direct insight into how programmable polymerization governs the overall film formation process.

Author reply: Thank you for your constructive suggestion. Visualizing the buried interface is indeed crucial for understanding how the stepwise polymerization strategy governs the film formation. In response to your suggestion, we first peeled off the perovskite films with an UV adhesive and examined the morphology of the exposed bottom surface (**Figure S7**). There were noticeable nanovoids and discontinuous contact at the bottom surface of the pristine perovskite film that likely formed because hydrophobicity led to insufficient wetting. By contrast, with the assistance of stepwise polymerization between BFDGE and IPDA, the tailored precursor enabled compact and void-free morphology. Furthermore, the target film exhibits improved crystallinity with large, continuous grains both the bottom and the top surface, implying that the programmable polymerization plays an important role in optimizing the overall film formation process. We have included this in the revised manuscript, and the changes are also highlighted.

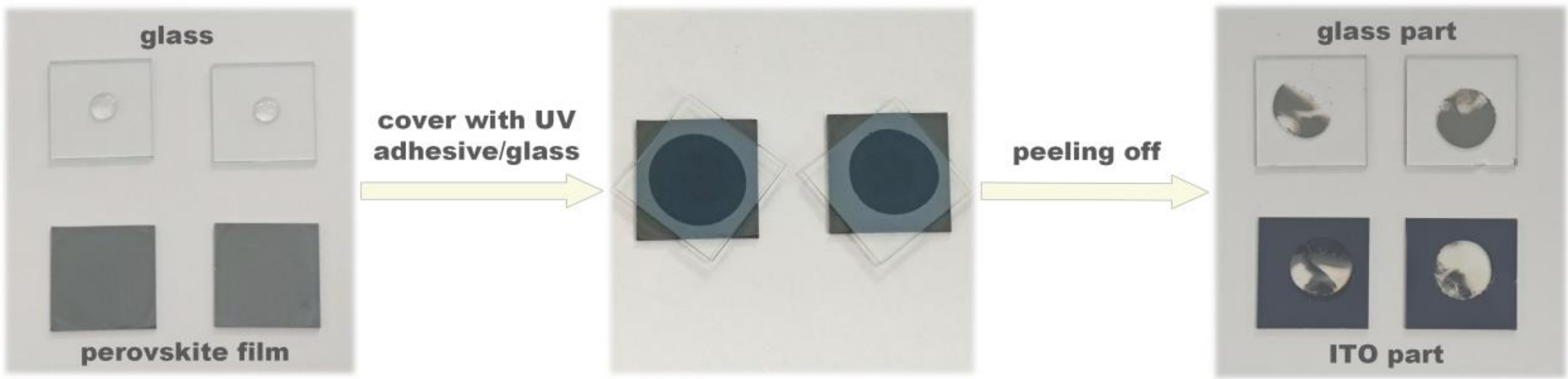

Figure R1. Schematic and photographs demonstrate the mechanical peeling test used to evaluate interfacial adhesion with a glass/ITO/SAMs/Perovskite architecture. Firstly, the perovskite film was covered with UV adhesive/glass. Following 2 minutes of curing, a tensile peeling force was applied, separating the structure into two distinct sections: an "ITO part" (including the ITO/SAMs substrates and possible perovskite remnants) and a "glass part" (consisting of the cover glass, UV adhesive, and the upper functional layers). Samples were chosen such that sufficient perovskite remained on the glass side after peeling, ensuring that the signal predominantly reflects the bottom region of perovskite layer near the buried interface.

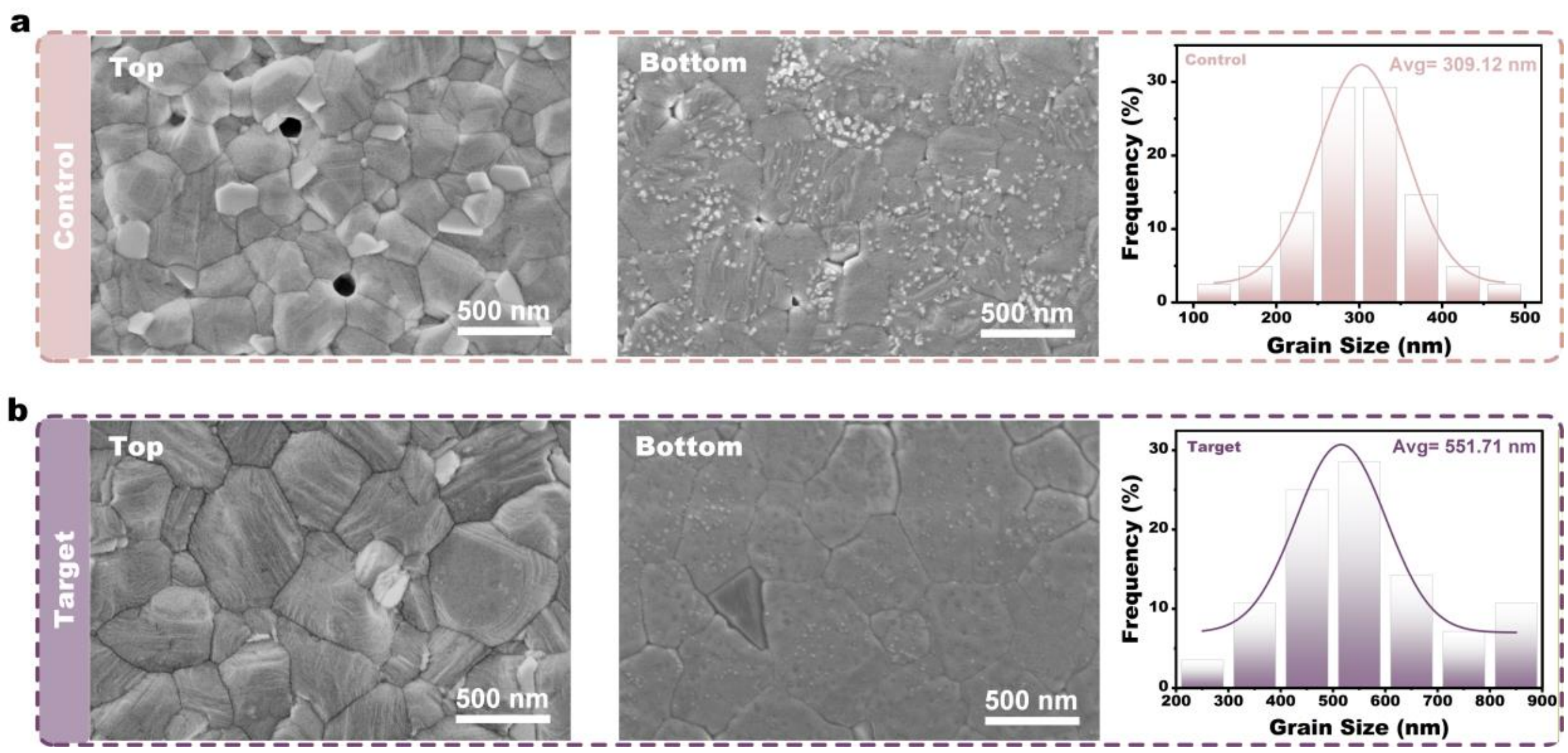

Figure S7. SEM images and the corresponding grain size distribution of the control (a), target (b) perovskite films.

2. The manuscript demonstrates the effectiveness of the programmable polymerization strategy on 4PADCB SAMs. To better assess its broader applicability, it would be valuable to test whether the tailored perovskite ink can also yield high-quality, uniform films on other types of SAMs.

Author reply: We thank the reviewer for this insightful comment. Demonstrating the broader applicability of our stepwise polymerization strategy is indeed vital for its potential in various device architectures.

To address this, we extended our contact angle measurements to a wide range of SAMs, including 4PADCB, Ph-PACz, 4PABCz, MeO-4PACz, and Me-4PACz. As summarized in **Figure S4**, the tailored perovskite ink demonstrated a consistent and notable reduction in contact angles across all tested SAMs compared to the control ink.This uniform improvement underscores the substrate-agnostic nature of our strategy. It confirms that the enhancement in wettability is driven by the intrinsic modulation of the precursor's liquid-vapor interfacial tension and the dynamic formation of polar groups, rather than being limited to a specific substrate interaction. We believe these results firmly establish our approach as a versatile and robust platform for high-performance large-area perovskite photovoltaics. We have included this in the revised manuscript and the changes are also highlighted.

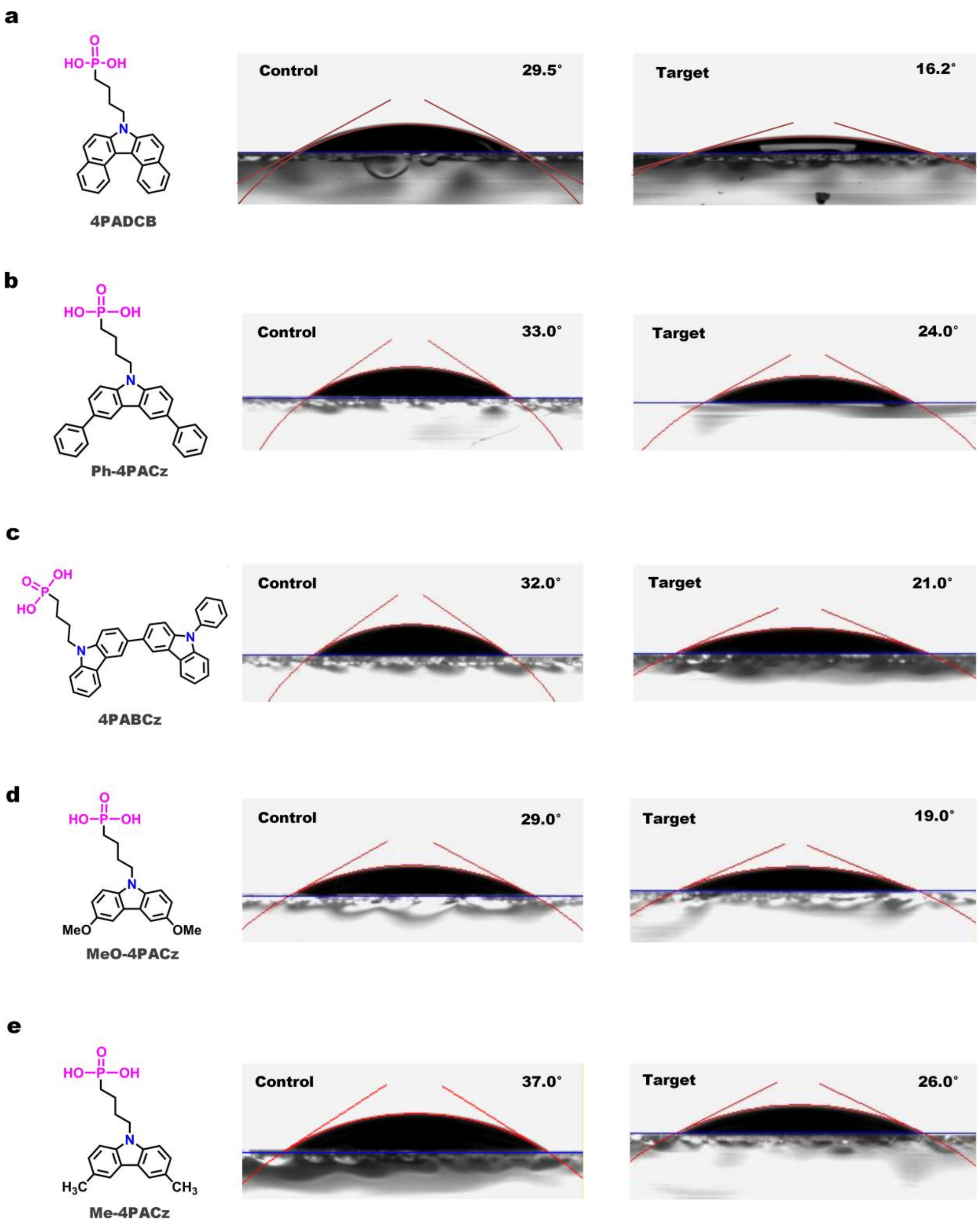

Figure S4. Comparison of contact angles for control and target perovskite inks on diverse SAM-based hole transport layers.

3. The authors state that programmable polymerization enables tailored perovskite ink. In the context of scalable fabrication, the adhesion between the perovskite ink and the underlying hydrophobic SAM layer is of great importance. Please provide supporting evidence on this aspect.

Author reply: We appreciate the reviewer's insightful comment regarding the importance of adhesion between the perovskite ink and the hydrophobic SAM layer for scalable fabrication. To provide direct evidence of enhanced interfacial affinity and wetting stability, we monitored the dynamic drying process of perovskite ink droplets on hydrophobic 4PADCB-modified substrates under ambient conditions.

As illustrated in **Figure S33a** and **Figure S33b**, the droplet volume decreased during solvent evaporation, causing the contact line (the edge of the droplet) to move, with the coverage defined as the ratio of the droplet's projected area on the substrate during drying to its initial area. As a consequence, the coverage of pristine ink reduced to 89.3%. In contrast, the BFDGE + IPDA system enabled tailored perovskite ink, which maintained 96.8% coverage throughout the entire drying period (**Figure S33c**). The contact line behaved as if fixed to the hydrophobic substrate, a phenomenon typically observed on hydrophilic surfaces but rarely on hydrophobic ones. This indicates that the programmable polymerization effectively tailors the ink/substrate interface. The stepwise formation of hydroxyl-functionalized oligomers acts as a molecular "anchor", notably enhancing the interfacial affinity and preventing film retraction during large-area deposition (**Figure S33d**). We have included this in the revised manuscript, and the changes are also highlighted.

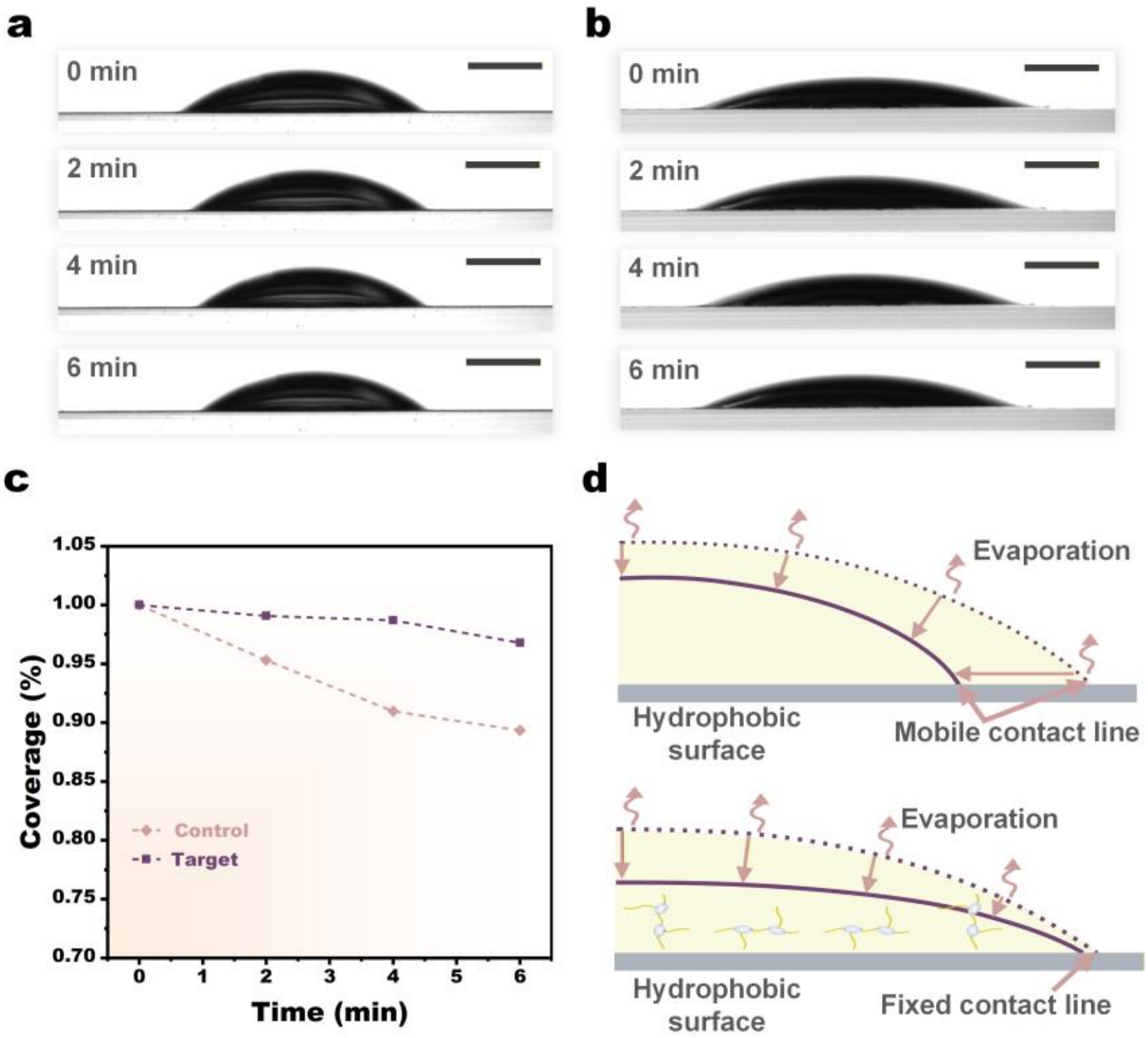

Figure S33. Comparison of droplet drying dynamics and surface coverage on hydrophobic SAM-based substrates. (a, b) Time-lapse optical images capturing the drying process of perovskite ink droplets on hydrophobic 4PADCB-covered substrates under ambient conditions: (a) control, (b) target. (c) Evolution of the normalized surface coverage of perovskite droplets as a function of drying time. (d) Schematic illustration for the shrinkage of the ink droplet drying on a hydrophobic substrate.

4. The mechanism underlying the N-type doping effect induced by the BEM, mentioned on Page 15, should be clarified.

Author reply: We thank the reviewer for this insightful question. The N-type doping effect induced by the BEM is primarily attributed to the Lewis base character of the functional groups within the resultant polymer network. Specifically, the nitrogen (N) atoms in the amines and the oxygen (O) atoms in the newly formed hydroxyl groups possess lone pair electrons. These electron-rich species can coordinate with uncoordinated Pb^{2+} or interact with iodine vacancies (V_I) at the perovskite grain boundaries and interfaces. The interactions involve the donation of electron density from the polymer scaffold to the perovskite lattice, thereby increasing the steady-state electron concentration and shifting the Fermi level (E_F) toward the conduction band (*Nat. Photon*. 20, 55-62 (2026); *Angew. Chem. Int. Ed.* 64, e202418606 (2025)). We will include this explanation and the relevant references in the revised manuscript to clarify this point.

5. The authors attribute the enhanced V_{OC} primarily to the suppression of non-radiative recombination at the perovskite/ETL interface via the programmable polymerization strategy. However, drawing this specific conclusion directly from the results in Figure 5F may not be fully rigorous. The authors should reconsider this interpretation and provide additional explanatory evidence.

Author reply: We appreciate the reviewer's meticulous comment regarding the rigorous interpretation of the V_{OC} enhancement. We fully agree that attributing the V_{OC} loss primarily to nonradiative recombination at the perovskite/ETL interface based solely on the film PLQY results was not sufficiently rigorous. In response to your comment, we have carefully revised the manuscript to provide a more balanced and nuanced interpretation. We now state that: "the target perovskite film exhibited a slight increase of PLQY value of 0.72%, versus the control (0.52%) sample. However, the average V_{OC} measured from *J-V* scans was 1.163 V for the target device, exceeding the control's 1.109 V by 54 mV. Therefore, the nonradiative recombination at the interface may be a limiting factor in the performance of control devices, implying the proposed programmable polymerization strategy plays a significant role in manipulating interfacial recombination."

We thank you again for reviewing our manuscript.

We sincerely thank all the reviewers for their thoughtful comments and constructive feedback, which have greatly helped us improve our manuscript. Do hope that the revised manuscript is now suitable for publication in ***Nature Communications***.

Best regards,

Prof. Ziyi Ge
Ningbo Institute of Materials Technology and Engineering
Chinese Academy of Sciences
Email: geziyi@nimte.ac.cn

Dear Reviewers,

We would like to express our sincere gratitude to the Reviewers for their time, expertise, and highly constructive feedback. We have carefully revised the manuscript according to their comments, and the corresponding changes in the manuscript are presented in **blue font**. Our responses to their comments along with the revisions are included here with. We hope that our manuscript now meets the requirements of**"*Nature Communications*"** as one of the field's leading journals.

If there are any other modifications you would recommend, we would like very much to further modify the manuscript, and we truly appreciate your help. We hope that our manuscript is considered favorably for prompt publication. Thank you very much once again for your help.

Point-by-Point Response to Reviewers

General Comment from Reviewer #2:

This manuscript reports an in-situ stepwise polymerization strategy using BFDGE and IPDA to improve perovskite film quality, device efficiency, stability, and scalability. Although the work presents high device efficiencies and large-area module results, fundamental flaws in mechanism clarity, data consistency, control experiments, characterization logic, and novelty remain unresolved. The manuscript does not meet the rigorous standards for publication. It can be considered in other journals below the standards of NC.

Author reply: We sincerely thank the Reviewer for dedicating their time to evaluate our manuscript. We fully respect the Reviewer's highly rigorous standards for *Nature Communications*. We acknowledge that our initial manuscript lacked certain real-time kinetic data and critical control experiments, which led to reasonable doubts regarding the mechanism and data consistency.

As suggested, we have addressed your overarching concerns by conducting a series of new, targeted experiments. As detailed in our point-by-point responses below, we have added real-time in situ kinetic monitoring, comprehensive individual-monomer control devices, and extensive statistical data to validate the reproducibility of our scalable fabrication. Furthermore, we have thoroughly distilled and highlighted the fundamental novelty of our stepwise polymerization strategy. We hope that this extensively revised manuscript, now supported by these robust experimental foundations and refined scientific insights, addresses your critical concerns and meets your stringent standards.

1. I think that the authors failed to give a clear and proven “Stepwise Polymerization” mechanism. The authors claim a stepwise ring opening polymerization synchronized with perovskite crystallization, but provide no real time kinetic evidence. No in situ monitoring of polymerization rate, molecular weight evolution, or cross linking degree during annealing.The reaction between BFDGE and IPDA at 90-100 °C is too fast to be “stepwise”; the claimed “stepwise” control is speculative and unsupported. The authors cannot distinguish whether performance improvements come from polymer cross-linking, small molecule passivation, or solvent effects.

Author reply: Thank you for your critical and insightful comments. We completely agree that a rigorous mechanistic explanation and comprehensive control experiments are essential. Here, we clarify the "stepwise" nature of our strategy and address the concerns regarding the reaction kinetics, solvent effects, and the distinction from small-molecule passivation.

1. Clarification of “Stepwise Polymerization” Mechanism and Kinetic Evidence

We apologize if the term “stepwise” caused any ambiguity in our initial manuscript. In this work, “stepwise” specially refers to the two distinct physical and chemical stages during the perovskite film fabrication process, which we have now validated through in-situ Fourier-transform (FTIR) spectroscopy:

(1) Stage 1: Precursor Ink Preparation at Room Temperature. To strictly maintain the exact solvent ratio, we formulated the modified ink by initially withholding 100 uL of DMF per 1 mL of the precursor solution. Subsequently, we introduced 50 uL of BFDGE-DMF solution (8 mg/ml) and 50 uL of IPDA-DMF solution (0.5 mg/ml, 5 wt% of BFDGE). As shown in **Figure 1c**, in situ FTIR monitoring at room temperature reveals that the characteristic stretching vibration of the epoxy group at ~914 cm^{-1} gradually weakens but persistently remains. This kinetic evidence indicates that BFDGE and IPDA undergo a preliminary, yet incomplete, reaction upon mixing. This partial reaction generates hydroxyl-containing intermediate oligomers, which notably alters the surface tension of the tailored precursor ink (from 35 to 28.5 mN/m), effectively improving its wettability and spreadability on the hydrophobic SAM substrate (As illustrated in the revised **Figure 1b**, left).

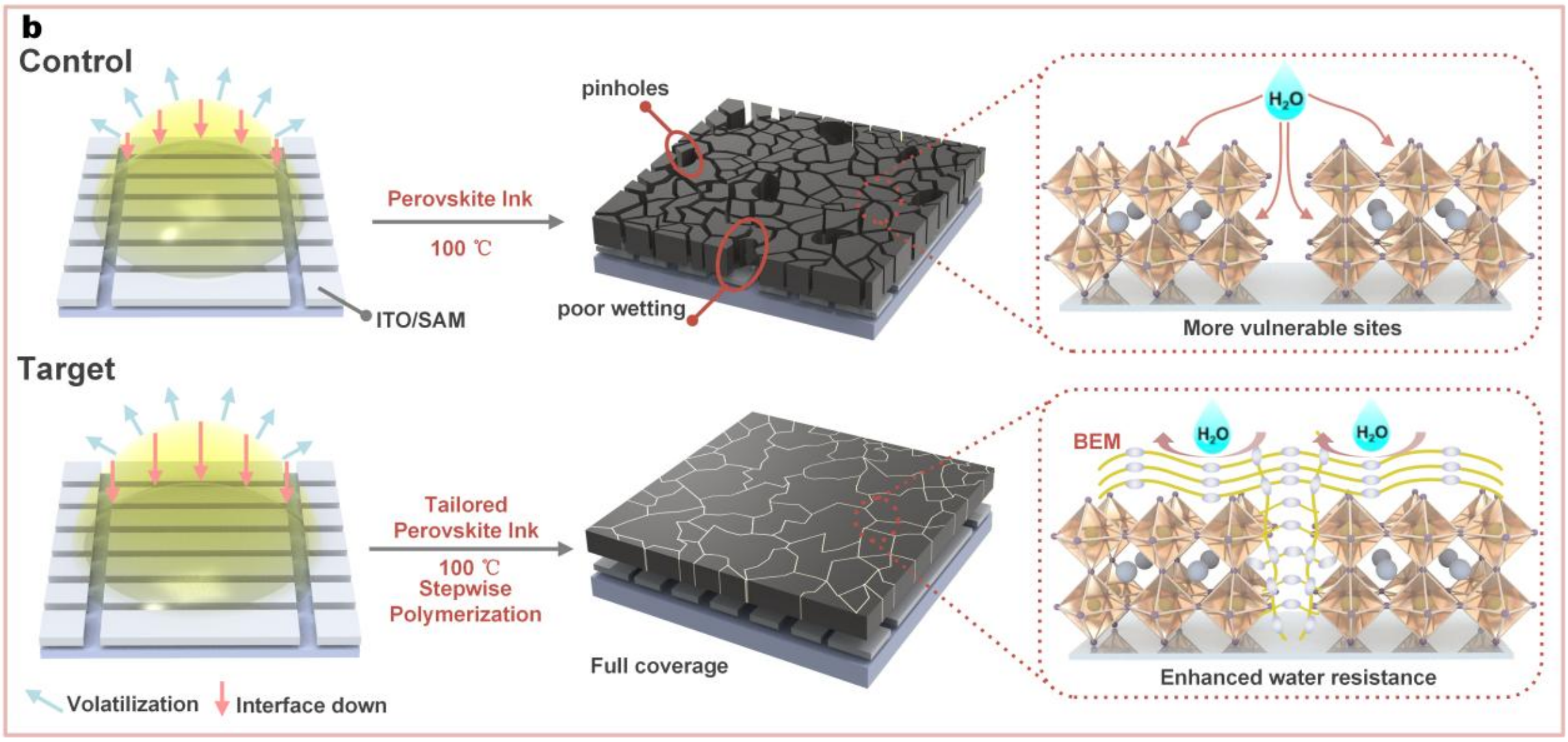

Figure 1b. Schematic illustration of the proposed stepwise polymerization strategy that enables scalable fabrication and stability enhancement.

(2) Stage 2: Thermal Annealing at 100 °C. During the wet film annealing, the ring-opening polymerization between BFDGE and IPDA is accelerated and synchronizes with perovskite crystallization. To directly address your concern regarding the reaction rate at 100 °C, we continuously monitored the FTIR spectra during the heating process. Upon continuous heating at 100 °C, the residual epoxy peak at 914 cm^{-1} progressively diminishes and completely disappears after approximately 10 minutes. (**Figure 1d**). The real-time kinetic results demonstrate that the polymerization reaction is not an instantaneous flash reaction. Instead, it proceeds at a moderate, measurable rate, which perfectly overlaps with the critical period of perovskite nucleation and crystal growth. When combined with our in situ absorption, in situ PL, and in situ XRD results, this confirms that the concurrent polymerization process modulates and optimizes perovskite crystallization dynamics. These two stages synergistically enable the reliable and uniform fabrication of large-area perovskite films.

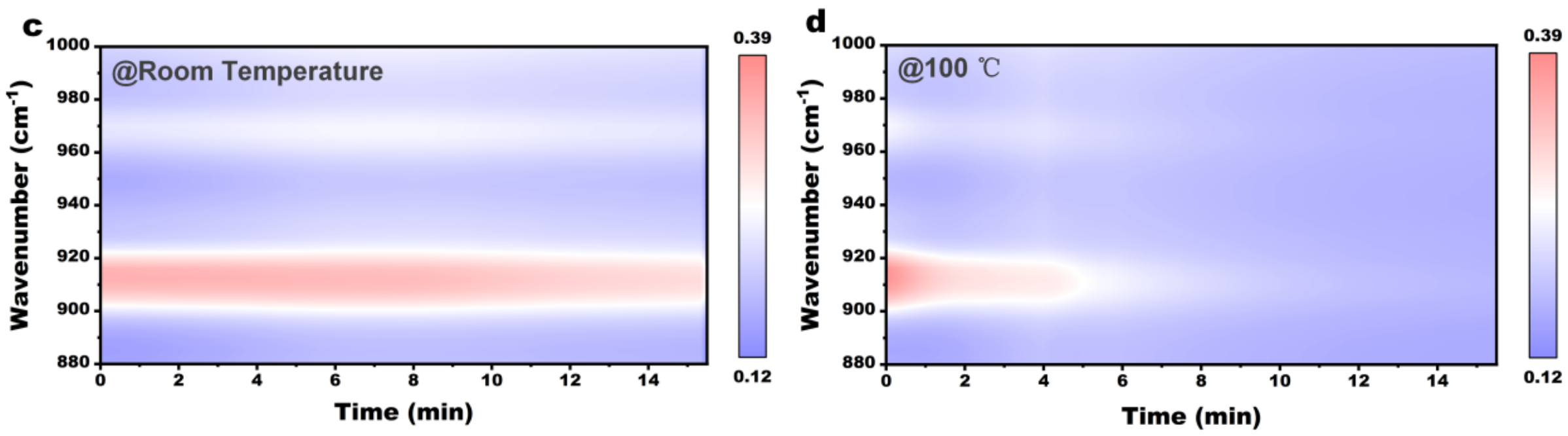

Figure 1c, d. In situ FTIR spectroscopy of BFDGE and IPDA mixture at room temperature (c) and 100 °C (d).

2. Ruling out Solvent Effects and Small-Molecule Passivation

We thank the Reviewer for suggesting these critical control experiments to distinguish the origins of the performance improvements. We have addressed this from two aspects:

(1) Ruling out Solvent Effects: As detailed in Stage 1, the total volume of DMF in the target ink was strictly kept identical to that of the pristine precursor ink (by substituting 100 uL of pure DMF with 100 uL of additive-containing DMF). Therefore, the overall solvent ratio remains entirely unchanged, unequivocally ruling out any performance variation stemming from solvent volatilization effects.

(2) Ruling out Small-Molecule Passivation: To distinguish the effects of the polymer cross-linking scaffold from small-molecule passivation, we fabricated and tested new control devices modified solely with BFDGE and solely with IPDA at matched concentrations. As shown in **Figure S21**, while the individual BFDGE- and IPDA-modified devices exhibited slight performance improvements over the pristine control (likely due to localized defect passivation from functional groups), their photovoltaic efficiencies notably lag behind those of the devices modified with the in situ stepwise polymerization strategy. This pronounced disparity in performance is primarily attributed to the profound optimization of the buried interface enabled by our stepwise polymerization strategy, coupled with the highly effective passivation of bulk defects by the robust 3D cross-linked network.

Your input has been invaluable in helping us strengthen the rigor of our work. We have included this in the revised manuscript, and the changes are also highlighted.

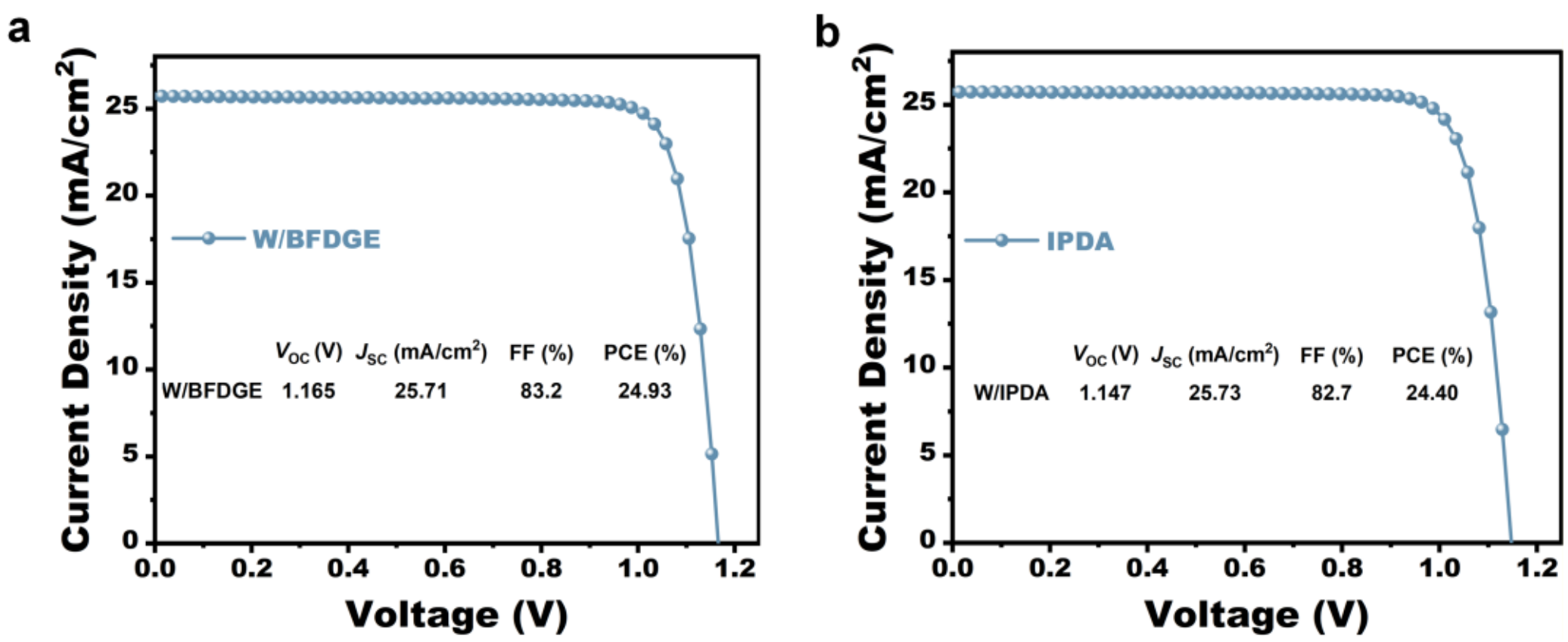

Figure S21. *J-V* curves of devices modified solely with BFDGE and solely with IPDA.

2. The critical control experiments are missing. No devices using pre polymerized BEM instead of in situ polymerization. No tests with BFDGE only or IPDA only at matched concentrations. No comparison with common cross-linkers to prove uniqueness of the BFDGE–IPDA system. No blank devices with equivalent DMF addition to rule out solvent effects.

Author reply: We sincerely thank the Reviewer for pointing out the need for comprehensive control experiments. Establishing rigorous baseline comparisons is indeed paramount to

proving the uniqueness and necessity of our in situ BFDGE–IPDA stepwise polymerization strategy. We have fabricated multiple batches of new control devices to address each of your concerns:

1. Devices using Pre-polymerized BEM vs. In-situ stepwise Polymerization: To demonstrate the indispensability of the in-situ stepwise polymerization approach, we synthesized fully pre-polymerized BEM and attempted to dissolve it in the precursor ink. As shown in **Figure R1**, because the fully cured epoxy-amine system forms a highly cross-linked 3D network, the pre-polymerized BEM is completely insoluble in DMF. It prone to form macroscopic gel aggregates, leading to inhomogeneous perovskite films. The photovoltaic performance of device fabricated from this ink notably lag behind the control and target devices.

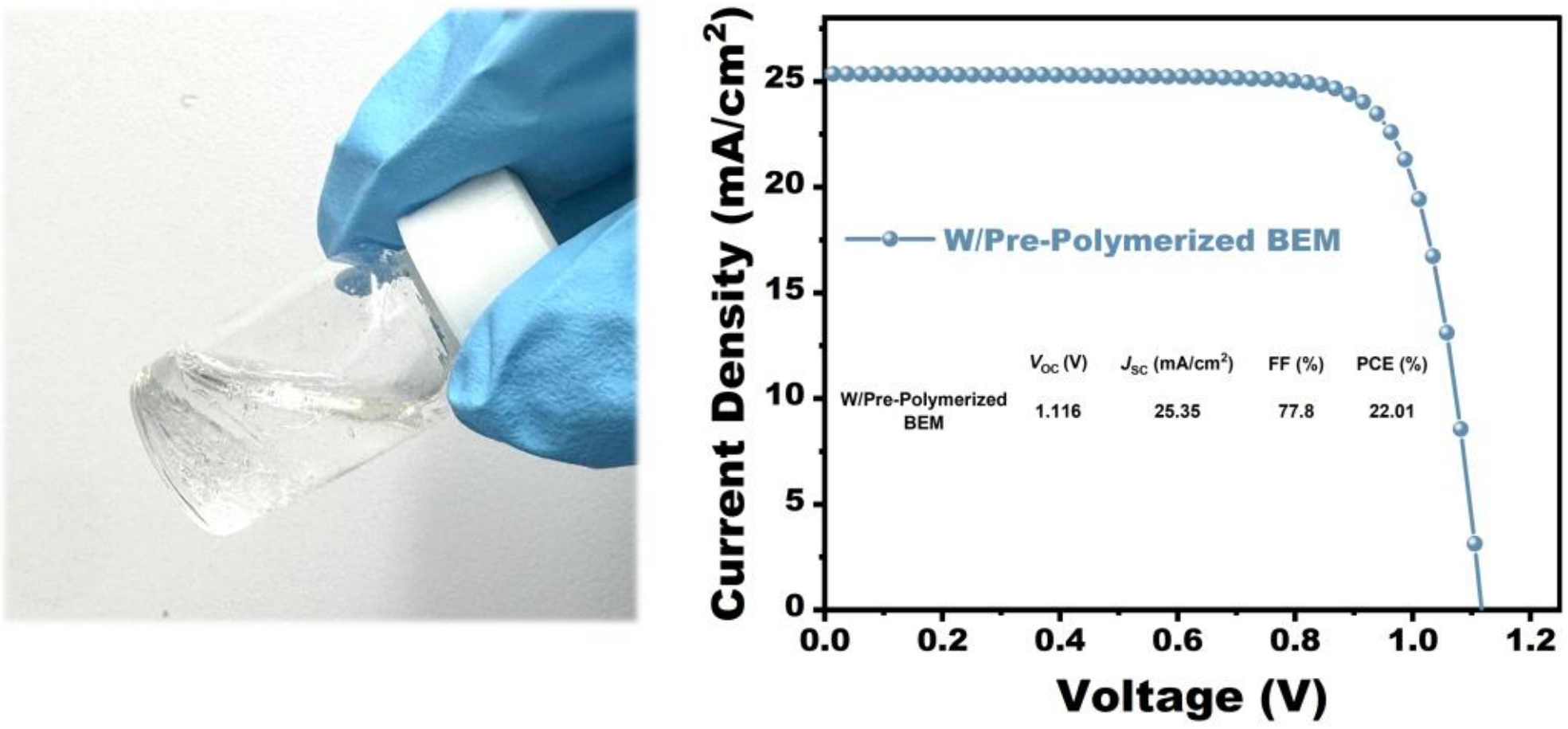

Figure R1. Photovoltaic performance of device with pre-polymerized BEM.

2. Comparison with Common Cross-linkers

To address the uniqueness of the BFDGE-IPDA system among common cross-linkers, we have summarized a comparison of state-of-the-art cross-linkable monomers in **Figure R2.** Benefiting from the buried interface manipulation and bulk passivation enabled by the stepwise polymerization strategy, the target devices exhibit competitive photovoltaic performance. Furthermore, the mild polymerization temperature of the BFDGE-IPDA system well matches the annealing requirements of inverted structure and flexible PSCs, while demonstrating outstanding potential for scalable fabrication. For a more detailed discussion on how this fundamental conceptual novelty differentiates our work from prior literature, please refer to our detailed response to Comment 6.

Crosslinkable Monomer	Polymerization T (℃)	PCE (%)	Operational stability	Scalability	Refs.
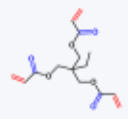 TMTA	140	19.26	78.5%/400h ISOS-L-1	no	*Nat. Commun.* 9, 3806 (2018)
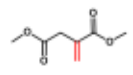 DI	90	23	85.7%/504h ISOS-L-1	no	*Adv. Mater.* 32, 1907769 (2020)
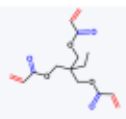 TMTA	150	22.39	80%/1248h 0.8 SUN	no	*Adv. Mater.* 33, 2008487 (2021)
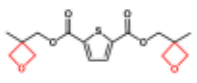 OETC	150	24.1	no	22.9%@1.004 cm^2	*Joule* 7, 398–415 (2023)
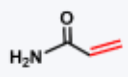 Am	100 & irradiated by sunlight for 10 min	24.45	80%/1008h ISOS-L-1	20.31%@ 33cm^2	*Adv. Energy Mater.* 14, 2302743 (2024)
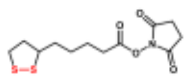 FTA	60	25.48	91.8%/1000h ISOS-L-1	19.53%@10.24 cm^2	*Sci. Adv.* 11, eadr2290 (2025)
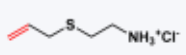 PYA	100	27.37	90.4%/2110h ISOS-L-2	23.03%@69 cm^2	*Nat. Photon.* (2026). https://doi.org/10.1038/s41566-026-01918-y
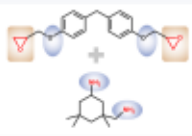 BFDGE & IPDA	90	27.16	93.3%/1000h ISOS-L-1	22.60%@655.2 cm^2	This work

Figure R2. Comparison with Common Cross-linkers

3. Individual Monomer and Solvent Ratio Controls: As detailed in our response to comment 1, we have strictly ruled out solvent effects via a precise volume-replacement protocol. Furthermore, as summarized in the **Table R1**, individual monomer controls (BFDGE-only and IPDA-only) and pre-polymerized BEM yield inferior efficiencies compared to the in situ stepwise polymerization strategy modified samples, confirming the effectiveness of this strategy.

Table R1. Summary of photovoltaic parameters for devices based on various control configurations.

Sample	V_{OC} (V)	J_{SC} (mA/cm^2)	FF (%)	PCE (%)
Control	1.136	25.67	82.6	24.10
W/BFDGE	1.165	25.71	83.2	24.93
W/IPDA	1.147	25.73	82.7	24.40
W/Pre-polymerized BEM	1.116	25.35	77.8	22.01
Target	1.190	26.48	86.2	27.16

3. Severe data Inconsistency and unreliability. Integrated J_{SC} from EQE does not closely match J_{SC} from *J-V* curves. Trap density decreases but carrier lifetime trends are over interpreted.YOY values and thermal stability data lack error bars and statistical repeatability.

Author reply: We sincerely appreciate the Reviewer's meticulous examination of our data. We have carefully re-evaluated our raw data, conducted new measurements where necessary, and revised our claims to ensure rigorous scientific accuracy.

1. Addressing the Mismatch between Integrated J_{SC} from EQE and J_{SC} from *J-V* Curves

We have thoroughly re-checked our *J-V* and EQE data and would like to clarify the discrepancies. For flexible devices, the *J-V* measured J_{SC} is 25.54 mA/cm^2, while the integrated J_{SC} from the EQE spectrum is 24.85 mA/cm^2. The deviation is 2.78%, which falls well within the widely accepted margin of error (<5%) for PSCs. For rigid devices, we noticed a relatively large mismatch in our initial submission (26.48 mA/cm^2 from *J-V* vs. 25.25 mA/cm^2, an error of 4.87%). Upon rigorous re-examination of our testing conditions, we indentified that the specific anti-reflective coating applied during that initial EQE measurement exhibited non-uniform spectral transmittance. This inadvertently altered the spectral shape of the EQE curve, causing an artificial underestimation of the photoresponse in certain wavelength bands. To rectify this, we replaced it with a broadband AR coating and re-measured the device. The newly integrated J_{SC} from the corrected EQE spectrum is 25.58 mA/cm^2, reducing the mismatch to a highly reliable 3.51%. We have updated the corresponding curve in **Figure S27b**.

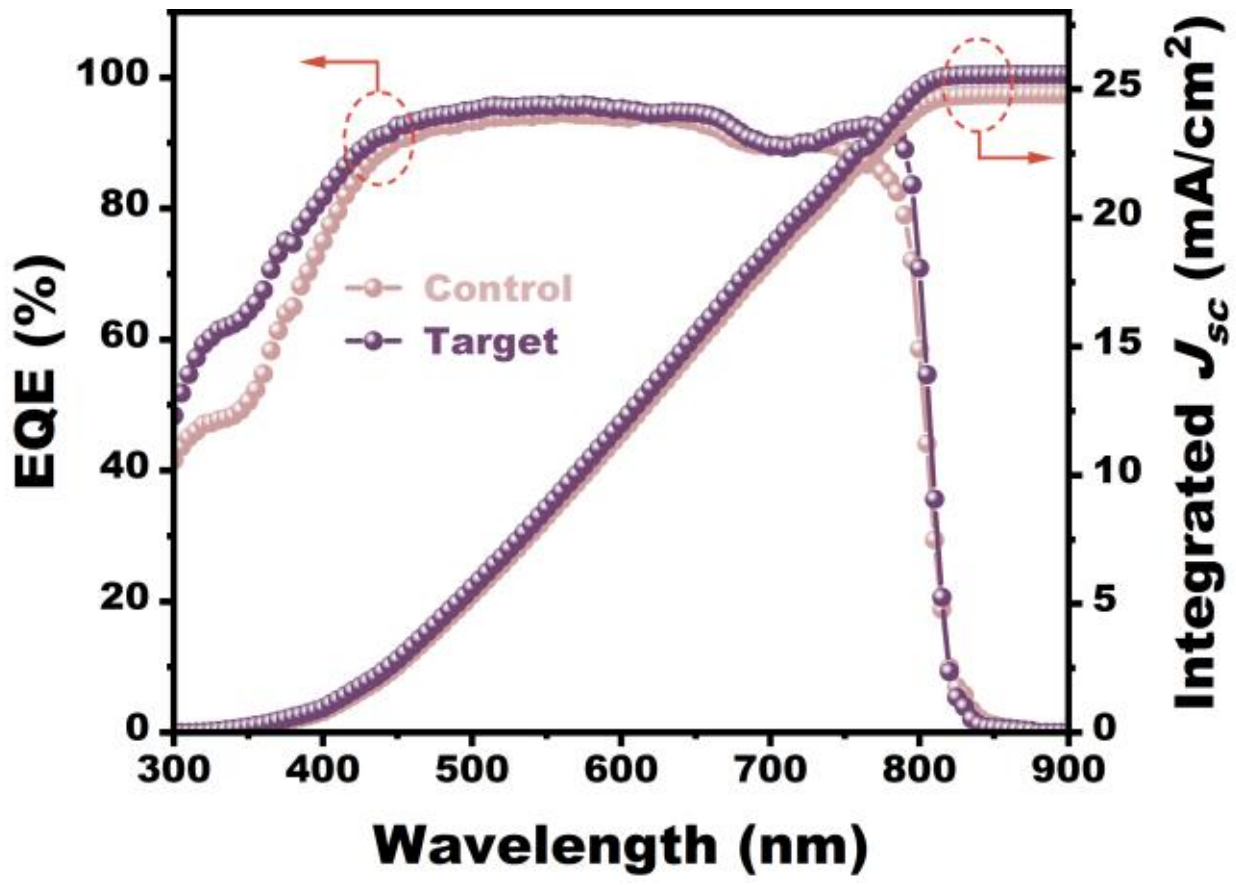

Figure S27b. EQE spectra and the corresponding integrated current density of rigid PSCs.

2. Clarifying the Interpretation of Trap Density and Carrier Lifetime

We agree with the Reviewer that the carrier lifetime and trap density trends should not be over-interpreted. Accordingly, we have revised the relevant statements in the manuscript: "TRPL decay curves measured from the perovskite bottom side show a carrier lifetime of 1.9 μs for the target sample, ~1.58 times longer than that of the control (fitted by **Note S2**, Supplementary Information), indicating suppressed non-radiative recombination in the perovskite layer."

3. Providing Error Bars and Statistical Repeatability for PLQY and Thermal Stability Data

We apologize for the omission of error bars in our previous PLQY and stability plots. As shown in the revised **Figure 5d**, we have added error bars to the PLQY experimental results. Furthermore, we have updated the thermal stability by plotting the average values with standard deviation error bars based on a batch of independent modules (**Figure 6c**).

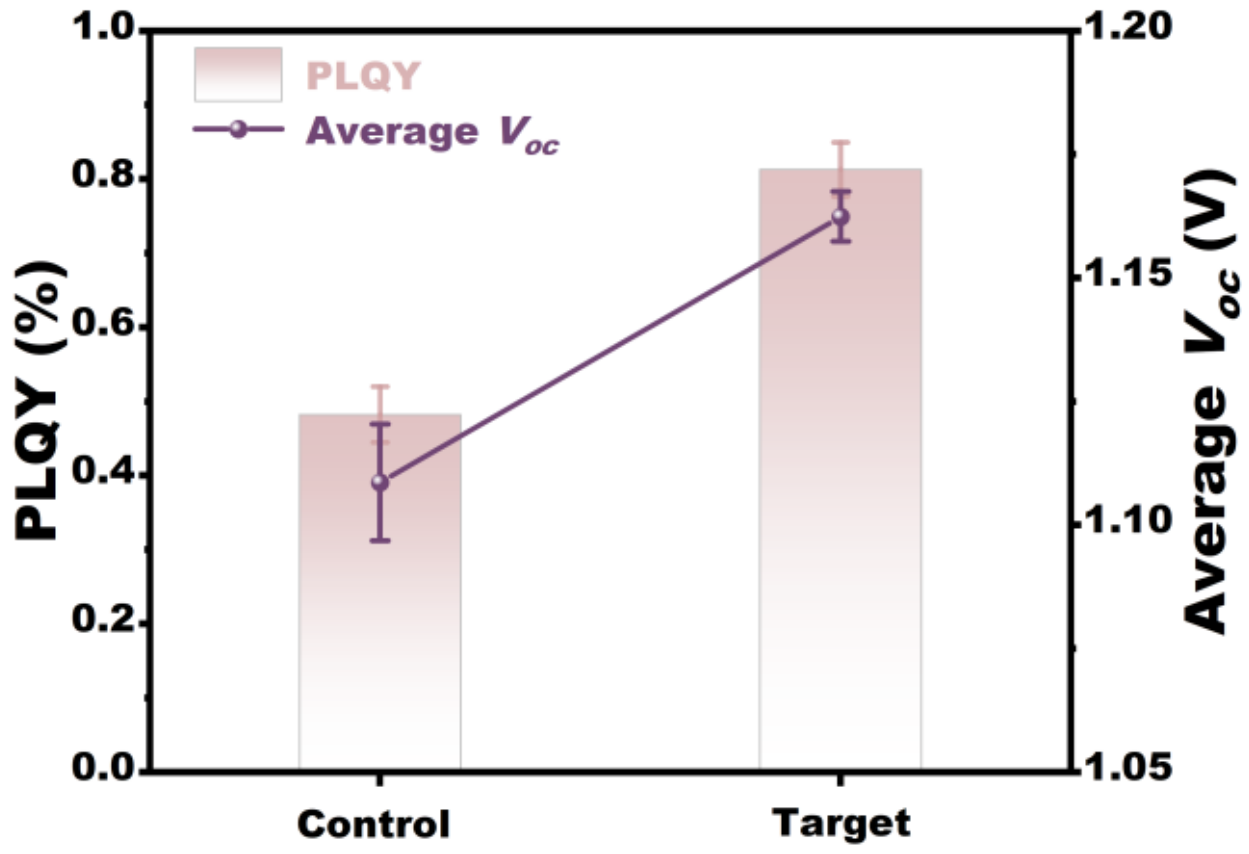

Figure 5d. Comparison of the PLQY values and the measured average V_{OC} of f-PSCs.

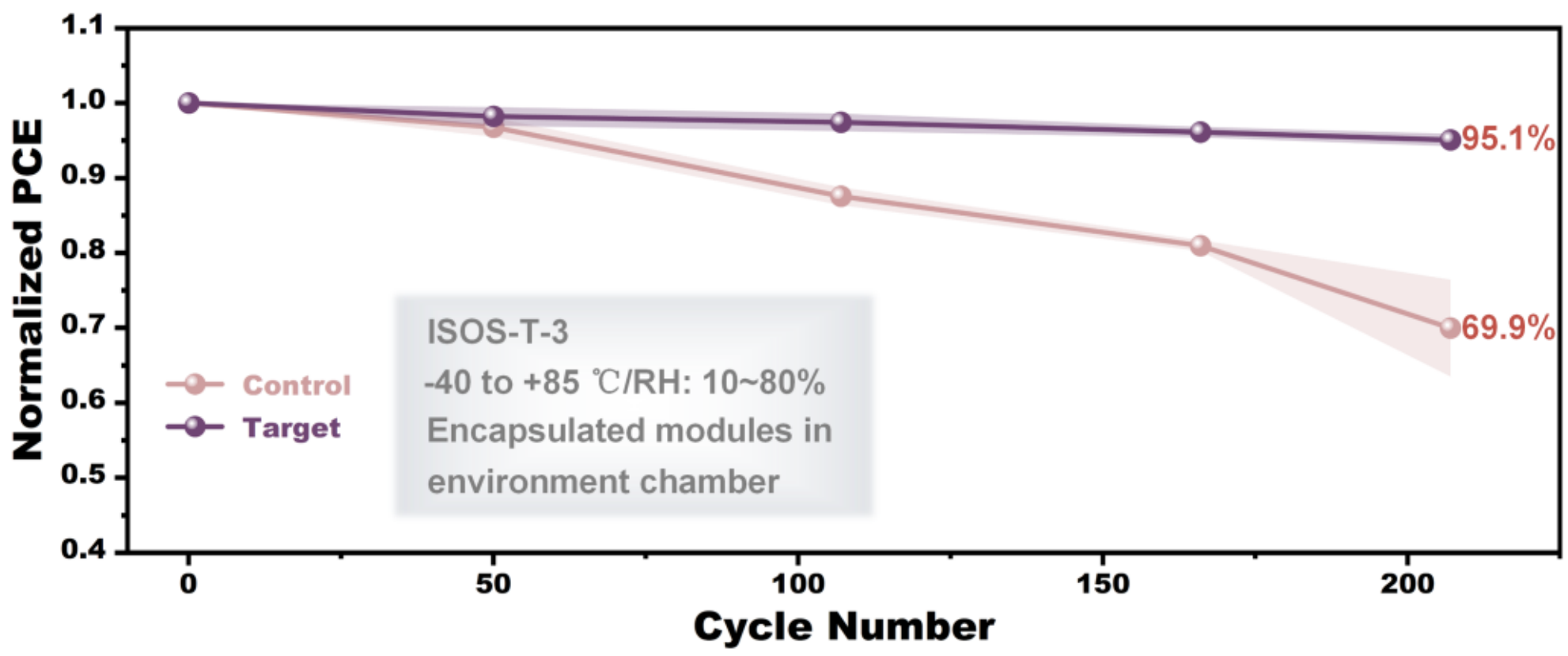

Figure 6c. Thermal cycling stability of encapsulated modules aged between -40 °C to +85 °C (ISOS-T-3).

4. Overclaimed Scalability and Module Fabrication. No uniformity mapping, no yield statistics, and no reproducibility data for 30 × 30 cm^2 modules.

Author reply: Many thanks to the reviewer for highlighting the importance of statistical reliability and uniformity validation for large-area module fabrication. Firstly, we sincerely apologize that we are unable to provide uniformity mapping for the 30 × 30 cm^2 modules. Currently, the sample chamber and scanning range of our available photoluminescence (PL) and electroluminescence (EL) mapping equipment cannot accommodate substrates of this massive dimension.

However, to establish the fundamental proof of concept regarding uniformity, we have already provided high-resolution PL mapping 5 × 5 cm^2 perovskite films (**Figure S36**) and EL mapping for 5 × 5 cm^2 minimodules (**Figure S37**) in the Supplementary Information. These results clearly and visually demonstrate that the stepwise polymerization strategy effectively mitigates localized non-emissive defects and ensures highly homogeneous coating over scaled-up areas compared to the control samples.

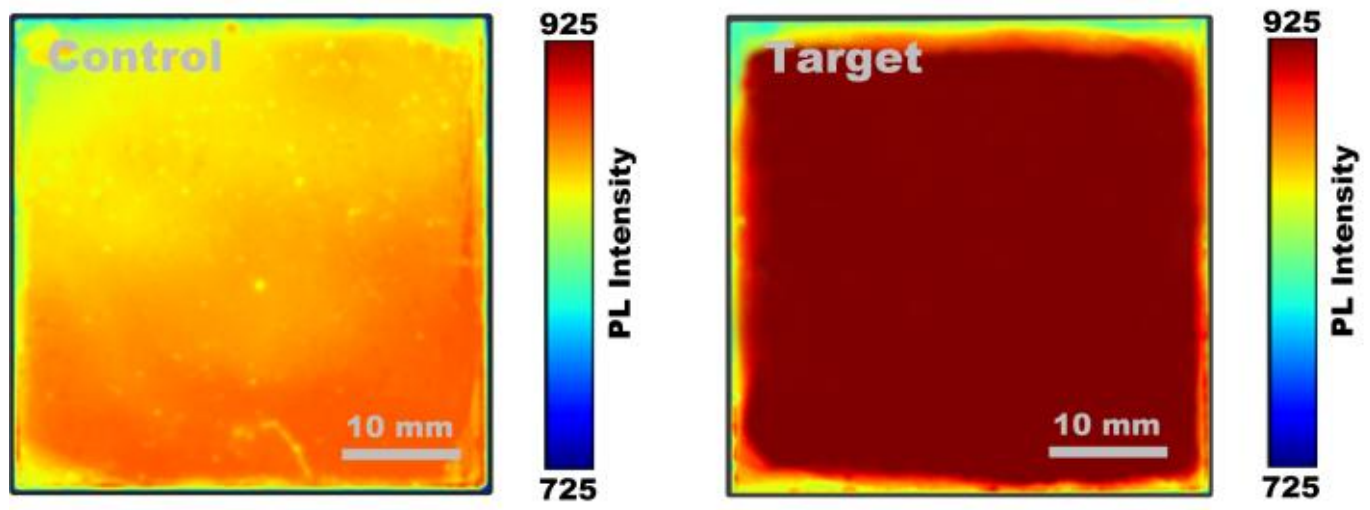

Figure S36. PL mapping images of perovskite films spin-coated on 5×5 cm^2 substrate.

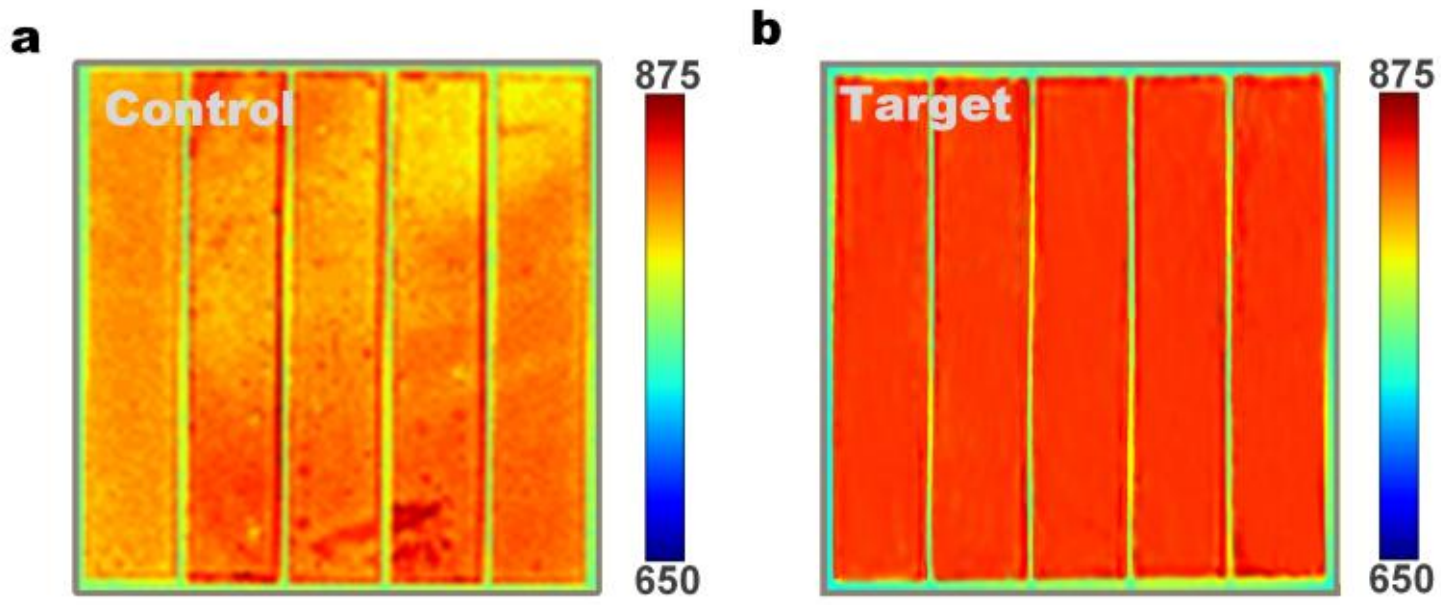

Figure S37. EL imaging of the (a) control and (b) target minimodules.

Additionally, to directly address your concern regarding the reproducibility at the sub-module scale, we have summarized the photovoltaic parameters of 10 independent 30 × 30 cm^2 submodules fabricated using our in situ stepwise polymerization strategy. As shown in the newly added **Figure S39** and **Table S10**, these submodules exhibit a remarkably narrow performance distribution, demonstrating excellent statistical repeatability. Do hope these clarifications and additional experimental results address the reviewer's concerns. We have included this in the revised manuscript, and the changes are also highlighted.

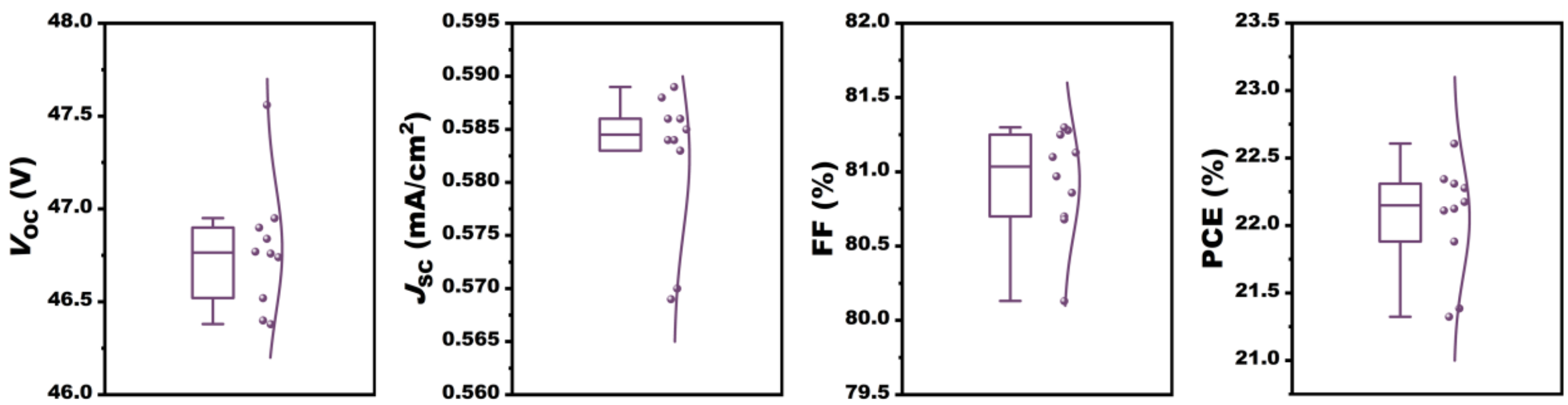

Figure S39. Statistical distribution of photovoltaic parameters for ten independent 30 × 30 cm^2 target submodules.

Table S10. Statistics of photovoltaic parameters for 10 individual 30 × 30 cm^2 submodules.

Sample	V_{OC} (V)	J_{SC} (mA/cm^2)	FF (%)	PCE (%)
Target	46.78±0.34	0.582±0.007	80.94±0.36	20.05±0.41

5. HRTEM and EDX line scans do not convincingly prove BEM exists only at grain boundaries.

Author reply: We appreciate the reviewer's rigorous and insightful comment. We completely agree that making an absolute claim that BEM exists only at grain boundaries is an overstatement, given the inherent limitations of localized micro-scale characterization techniques (such as HRTEM and EDX) and the low overall concentration of the BEM additive in the bulk film. In response, we have adjusted our claims and provide the following physical and macroscopic context to clarify our stance. Firstly, from the crystallographic perspective, owing to the molecular size and conformational flexibility of polymer additive, these molecules are expected to preferentially reside in structurally open regions rather than incorporate in the rigid perovskite lattice. This physical mechanism is widely accepted and corroborated by similar HRTEM/EDX characterizations in other state-of-the-art works utilizing cross-linked polymers (e.g., *Nat. Photon.* 2026, 10.1038/s41566-026-01943-x; *Nat. Commun.* 9, 3806, 2018; *Joule* 7, 398-415, 2023). Additionally, we conducted thermogravimetric analysis (TGA) of the BEM (as shown in **Figure R3**). The results demonstrate that the polymer remains completely stable up to 250 °C, proving that it survives the 100 °C perovskite annealing process intact. Furthermore, our Time-of-Flight Secondary Ion Mass Spectrometry (ToF-SIMS) depth profiling (**Figure S29**) provides macroscopic evidence for the distribution of the BEM scaffold throughout the bulk phase of the film.

Combined, these results demonstrate that BEM is well-suited to serve as a 3D structural scaffold that permeates the bulk film and grain boundaries to passivate defects and relieve residual stress. To avoid any misinterpretation, we have corrected the absolute phrasing in our revised manuscript.

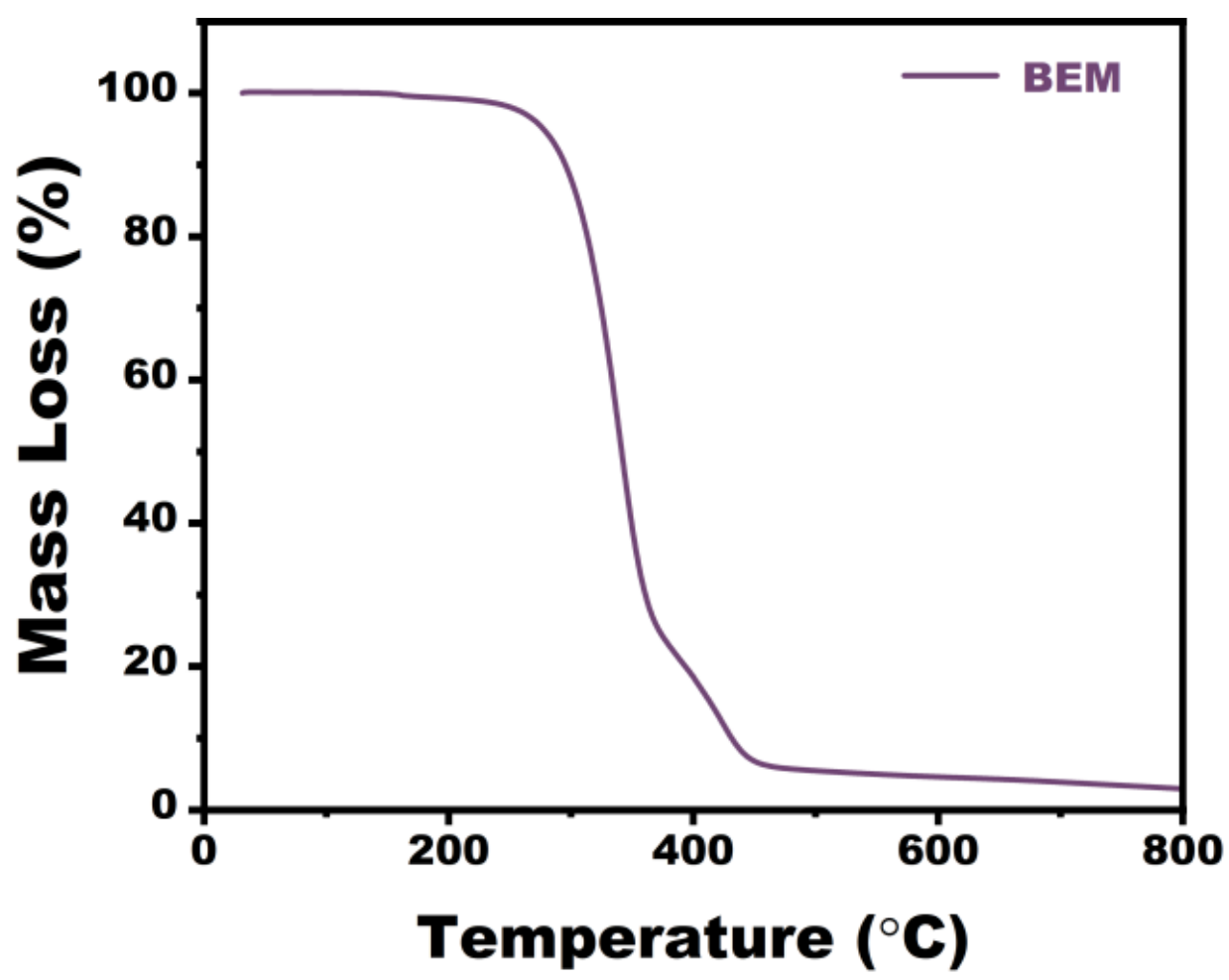

Figure R3. TGA curves of the BEM.

6. The novelty of this work is limited. As in situ polymerization, cross linking, and epoxy–amine chemistry for perovskites have been extensively reported. The authors do not highlight new scientific insights beyond prior work.

Author reply: We sincerely thank the Reviewer for urging us to further distill and articulate the core novelty of our work. In response, we would like to clarify the distilled innovative insights of our work, and we have made corresponding revisions in the relevant sections of the revised manuscript.

Firstly, while halide perovskite solar cells have achieved remarkable laboratory-scale efficiencies, the next critical hotspot for the research community is the technology transfer from laboratory to industrial applications. Although inverted (p-i-n) structures exhibit excellent compatibility for flexible and tandem applications, the inherently hydrophobic nature of self-assembled molecules (SAMs) substrates poses a formidable challenge for the uniform deposition of large-area perovskite films. As summarized in our new comparison table (**Figure R2**), although existing in situ polymerization strategies have played a positive role in improving film quality and device efficiency, their capability for large-area scalable fabrication remains insufficient.

Crosslinkable Monomer	Polymerization T (℃)	PCE (%)	Operational stability	Scalability	Refs.
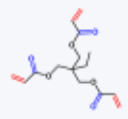 TMTA	140	19.26	78.5%/400h ISOS-L-1	no	*Nat. Commun.* 9, 3806 (2018)
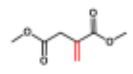 DI	90	23	85.7%/504h ISOS-L-1	no	*Adv. Mater.* 32, 1907769 (2020)
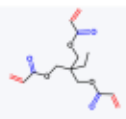 TMTA	150	22.39	80%/1248h 0.8 SUN	no	*Adv. Mater.* 33, 2008487 (2021)
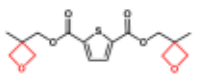 OETC	150	24.1	no	22.9%@1.004 cm^2	*Joule* 7, 398–415 (2023)
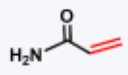 Am	100 & irradiated by sunlight for 10 min	24.45	80%/1008h ISOS-L-1	20.31%@ 33cm^2	*Adv. Energy Mater.* 14, 2302743 (2024)
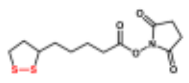 FTA	60	25.48	91.8%/1000h ISOS-L-1	19.53%@10.24 cm^2	*Sci. Adv.* 11, eadr2290 (2025)
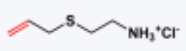 PYA	100	27.37	90.4%/2110h ISOS-L-2	23.03%@69 cm^2	*Nat. Photon.* (2026). https://doi.org/10.1038/s41566-026-01918-y
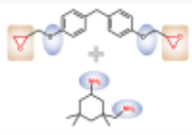 BFDGE & IPDA	90	27.16	93.3%/1000h ISOS-L-1	22.60%@655.2 cm^2	This work

Figure R2. Comparison with Common Cross-linkers

Secondly, recognizing that one of the key unique selling points for laboratory research on halide perovskites is the ability to utilize solution processing, we first introduce the concept of stepwise polymerization, shifting the focus from conventional crystallization regulation to upstream liquid-precursor ink engineering. Thus, providing a pioneering ink-design solution specifically tailored for the scalable fabrication of perovskite photovoltaics. As schematically illustrated in **Figure R4**, by utilizing the controllable stepwise kinetic nature of the BFDGE-IPDA system, we sequentially address the critical bottlenecks of scalable fabrication within a single ink system. Specifically, the pre-reaction at room temperature generates hydroxyl-functionalized oligomers that drastically reduce the interfacial tension, fundamentally solving the poor wetting issue of the ink on SAMs to ensure uniform large-area coating. Subsequently, during the 100 °C annealing process, the ring-opening polymerization synchronizes with perovskite crystallization, thereby regulating the crystal growth pathway. Ultimately, the resulting fully cross-linked 3D scaffold permeates the grain boundaries to buffer residual stress and passivate defects, translating to the exceptional long-term stability we observed under standard ISOS protocols (ISOS-L-1, ISOS-T-3, and UV stability). The ultimate proof of our conceptual novelty is the remarkably scalable result it unlocks. By compensating for the shortcomings of conventional in situ polymerization strategies in large-area expansion, our tailored ink-design strategy enabled the successful fabrication of a 655.2 cm^2 submodule with a certified PCE of 21.07%. We hope this comprehensive explanation clearly delineates how our work transcends conventional cross-linking reports.

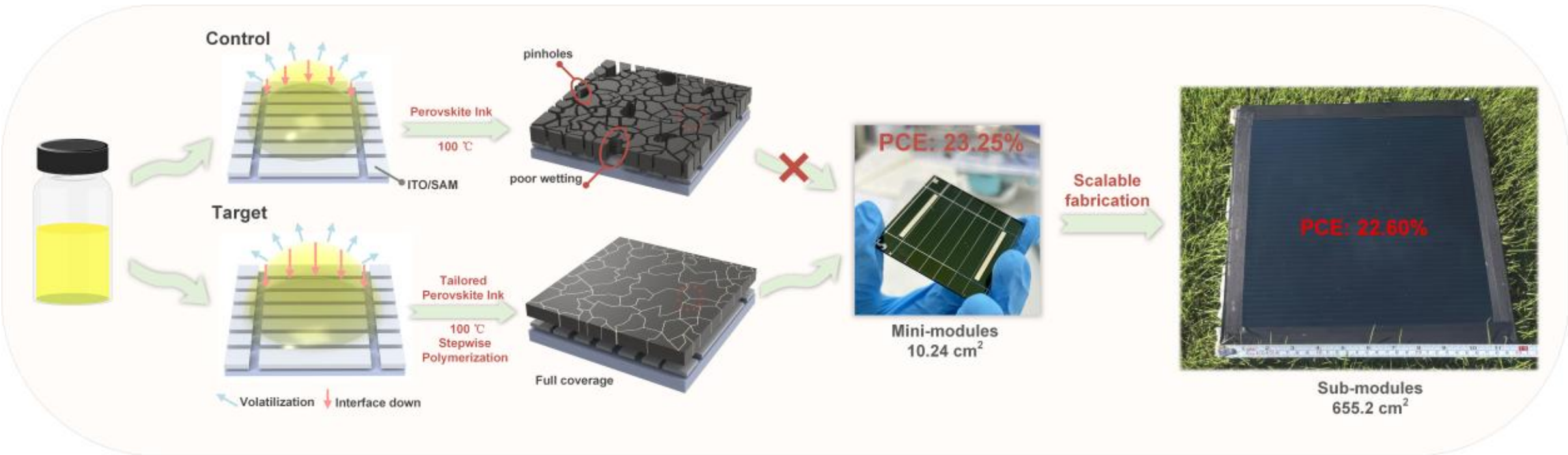

Figure R4. Schematic diagram of the scalable ink-design solution enabled by the in situ stepwise polymerization strategy.

We sincerely thank the reviewer again for your thoughtful comments and constructive feedback, which have greatly helped us improve our manuscript. Do hope that the revised manuscript is now suitable for publication in ***Nature Communications***.

Best regards,

Prof. Ziyi Ge
Ningbo Institute of Materials Technology and Engineering
Chinese Academy of Sciences
Email: geziyi@nimte.ac.cn

Dear Reviewer,

We would like to express our deepest gratitude to you for dedicating your time to evaluate our manuscript and for providing such a highly encouraging and positive assessment. Your recognition of our efforts and the thoroughness of our revisions is incredibly rewarding for our entire team.

Here is our responses to your comment.

Point-by-Point Response to Reviewers

General Comment from Reviewer #2:

Zhang et al. proposed a stepwise polymerization strategy to notably improve the scalable reliability of perovskite solar cells (PSCs), which is a critical issue for the manufacturing of solar modules. The authors incorporated an epoxy-terminated monomer (BFDGE) and a curing agent (isophorone diamine, IPDA). The stepwise reaction between BFDGE and IPDA enabled a tailored perovskite ink at room temperature, which ensures the uniform and reliable fabrication of perovskite films across large-area hydrophobic substrates; moreover, thermal annealing synchronizes the polymerization with perovskite crystallization, dynamically modulating the crystallization process. Overall, this work successfully addresses a key challenge and provides a convincing story that will attract a broad interest in this field.

The previous reviewer's critique of the 'stepwise polymerization' mechanism was well-founded, as claims of this nature in the literature often lack direct, fundamental data to substantiate them. The authors have executed a textbook revision. They did not merely offer verbal refutations; they performed extensive, well-designed experiments that directly transformed previous speculative claims into quantitative, verifiable physical insights. In-situ FTIR spectroscopy clearly validates the two-step reaction kinetics, while comprehensive control experiments confirm that the resulting polymer network, rather than individual additives, is uniquely responsible for the observed efficiency enhancement. This revised manuscript has been substantially improved, and the corrections requested by the reviewers have been exhaustively addressed. Hence, I believe that the present version is suitable for acceptance.

Author reply: We are deeply grateful for your profound recognition of our extensive experimental additions, particularly the in-situ FTIR kinetic tracking and the comprehensive individual-additive control evaluations. We are thrilled that these rigorous physical insights and data-driven validations have fully met your stringent standards.

Thank you once again for your invaluable support, your precise summary of our core scientific contributions, and your strong recommendation for acceptance.

Best regards,

Prof. Ziyi Ge
Ningbo Institute of Materials Technology and Engineering
Chinese Academy of Sciences
Email: geziyi@nimte.ac.cn
